# Giving the Green Light to Photochemical Uncaging of Large Biomolecules in High Vacuum

**DOI:** 10.1021/jacsau.3c00351

**Published:** 2023-10-16

**Authors:** Yong Hua, Marcel Strauss, Sergey Fisher, Martin F. X. Mauser, Pierre Manchet, Martina Smacchia, Philipp Geyer, Armin Shayeghi, Michael Pfeffer, Tim Henri Eggenweiler, Steven Daly, Jan Commandeur, Marcel Mayor, Markus Arndt, Tomáš Šolomek, Valentin Köhler

**Affiliations:** † Department of Chemistry, University of Basel, St. Johannsring 19, CH-4056 Basel, Switzerland; ‡ Vienna Faculty of Physics, University of Vienna, VDSP & VCQ, Boltzmanngasse 5, A-1090 Vienna, Austria; § Van’t Hoff Institute for Molecular Sciences (HIMS), University of Amsterdam, PO Box 94157, 1090 GD Amsterdam, The Netherlands; ∥ MS Vision, Televisieweg 40, 1322 AM Almere, The Netherlands; ⊥ Institute for Nanotechnology (INT), Karlsruhe Institute of Technology (KIT), P.O. Box 3640, DE-76021 Karlsruhe Eggenstein-Leopoldshafen, Germany; # Lehn Institute of Functional Materials, School of Chemistry, Sun Yat-Sen University, Guangzhou 510274, P. R. China

**Keywords:** photocages, molecular beams, gas-phase heterolysis, charge reduction, selective fragmentation, bioconjugation, bodipy chromophore, biomolecular mass spectrometry

## Abstract

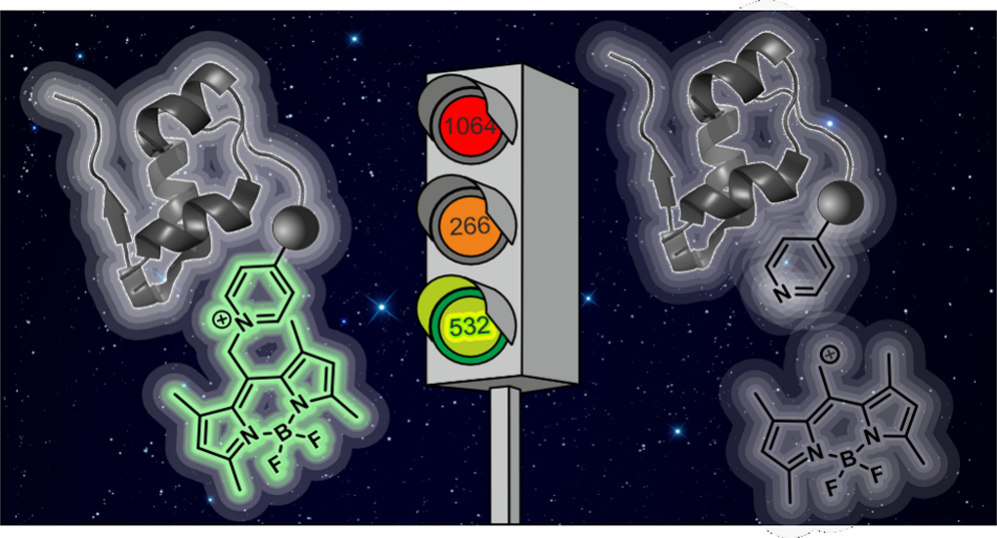

The isolation of biomolecules in a high vacuum enables experiments on fragile species in the absence of a perturbing environment. Since many molecular properties are influenced by local electric fields, here we seek to gain control over the number of charges on a biopolymer by photochemical uncaging. We present the design, modeling, and synthesis of photoactive molecular tags, their labeling to peptides and proteins as well as their photochemical validation in solution and in the gas phase. The tailored tags can be selectively cleaved off at a well-defined time and without the need for any external charge-transferring agents. The energy of a single or two green photons can already trigger the process, and it is soft enough to ensure the integrity of the released biomolecular cargo. We exploit differences in the cleavage pathways in solution and in vacuum and observe a surprising robustness in upscaling the approach from a model system to genuine proteins. The interaction wavelength of 532 nm is compatible with various biomolecular entities, such as oligonucleotides or oligosaccharides.

## Introduction

Peptides and proteins in the gas phase have attracted the interest of a growing research community^1−5^ because this environment allows accessing electrical, structural,^6,7^ and dynamical properties as a function of molecular charge,^8−11^ molecular adducts,^12^ or molecular orientation.^13,14^

The charge state of peptides and proteins in the gas phase determines not only their mass-to-charge ratio, the arguably most important read-out in mass spectrometry, but also plays a decisive role in their gas-phase structure,^8−11^ including protein–protein^15−17^ and protein–ligand (e.g., DNA)^18,19^ complexes. Fragmentation experiments with complementary methods^20,21^ such as collision-induced decomposition (CID),^22^ electron-transfer dissociation (ETD),^23^ electron capture dissociation (ECD),^24^ infrared multiphoton dissociation (IRMPD),^25,26^ and ultraviolet photodissociation (UVPD)^27−29^ that are employed to determine protein sequence and to probe structural integrity and dynamics often show a distinct charge-state dependence. Electrospray ionization^30^ (ESI) and matrix-assisted laser desorption ionization^31^ (MALDI) are the most common techniques to volatilize and ionize large biomolecules. While MALDI delivers short pulses of mainly singly charged ions in high vacuum, ESI can be used to prepare continuous beams of particles, typically with a mass-to-charge ratio of *m*/*z* < 3 kDa/e. In ESI-MS, the identification of ions can be hampered by signal overcrowding in the spectral region with low *m*/*z*-values, a frequently encountered problem in the analysis of complex mixtures.^32^ Gas-phase ion–ion reactions can serve to reduce the charge state and thereby deconvolute the spectra by shifting the signals to a higher and wider *m*/*z* range.^33,34^ Low charge states of massive proteins can be realized in ESI sources by reactions with an ionizing buffer gas^35−40^ or in ion traps by electron transfer or electron capture (ETnoD^41^ and ECnoD^42^). Further methods to manipulate the charge state include the addition of additives to the sprayed solution.^43^

Laser manipulation techniques have the potential to expand the wealth of methods even further by enabling time-dependent studies of charge-dependent processes.^44^ Such an approach may also lead to the optical neutralization of complex macromolecular ions in the gas phase, providing the means for interdisciplinary research, from quantum interferometry,^45^ electric and magnetic deflectometry^46,47^ to few-photon spectroscopy.^48^

UVPD^28^ has emerged as a powerful tool for protein fragmentation, enabling high sequence coverage in top-down approaches^49,50^ and also in de novo sequencing after protein digestion.^51^ UVPD mass spectrometry has been recently reviewed.^27−29^ In a separate field of research, short-wavelength UV light has been employed to ionize molecules,^52^ but this is limited to particles of low molecular mass and cannot be applied to intact proteins.^53,54^ A highly modified peptide with 25 tryptophan residues and a mass >20 kDa has, however, successfully been photoionized at 157 nm.^55^ Another application that relates to charge is the mapping of charge sites^56^ and salt bridges in proteins by UVPD fragmentation.^57^ UVPD is performed at various wavelengths, targeting either the intrinsic chromophores of proteins at short wavelength (157, 193, and 213 nm) or synthetic chromophores that are typically covalently linked to the analyte at higher wavelength (351, 355 nm) including visible light^58^ for selective fragmentation. While UVPD is often concerned with information-rich fragmentation, we aim to use the photocleavage of designed photocages to selectively remove charge from the labeled peptide or protein construct. Photocages consist of a chromophore adjacent to an intended breaking point, i.e., a bond that breaks preferentially upon photoexcitation of the chromophore.^59−70^ We employed nitrobenzylethers as photocages at 266 nm for charge reduction of peptides by 266 nm light in high vacuum and observed a strong dependency of the cleavage mechanism on peptide length.^71,72^ While heterolysis under charge reduction was observed for short peptides, charge-neutral H-transfer was dominant for peptides with six or more amino acids. Decorating the leaving groups with charges was necessary to allow for charge reduction, and it enabled the neutralization of insulin.

Here, we explore an even more accessible energy range for optical charge control with photocages that respond to 532 nm. This reduces the risk of competitive absorption by other naturally occurring aromatic units in large biopolymers.^73^ For proteins, the competitive absorption of tryptophan, tyrosine, and phenylalanine at short wavelength could otherwise lead to reduced efficiency of the uncaging and cause possibly undesired additional fragmentation. Photocages sensitive to visible light^61−64,67−70,74^ have recently been reviewed^59,65,66,75^ for applications in polar solutions. Here, we explore their behavior in the gas phase.

Selective charge control in high vacuum by photochemical uncaging is a significant challenge because (i) photocages in solution phase can behave differently than in the gas phase; (ii) their preferred mode of cleavage might depend on the size and the charge state of the peptide; (iii) suitable charge decoration can be synthetically demanding; and (iv) bioconjugation of the developed gas-phase photocages can present another hurdle due to the potentially limited stability of the constructs in solution.

## Results and Discussion

**Figure 1:**
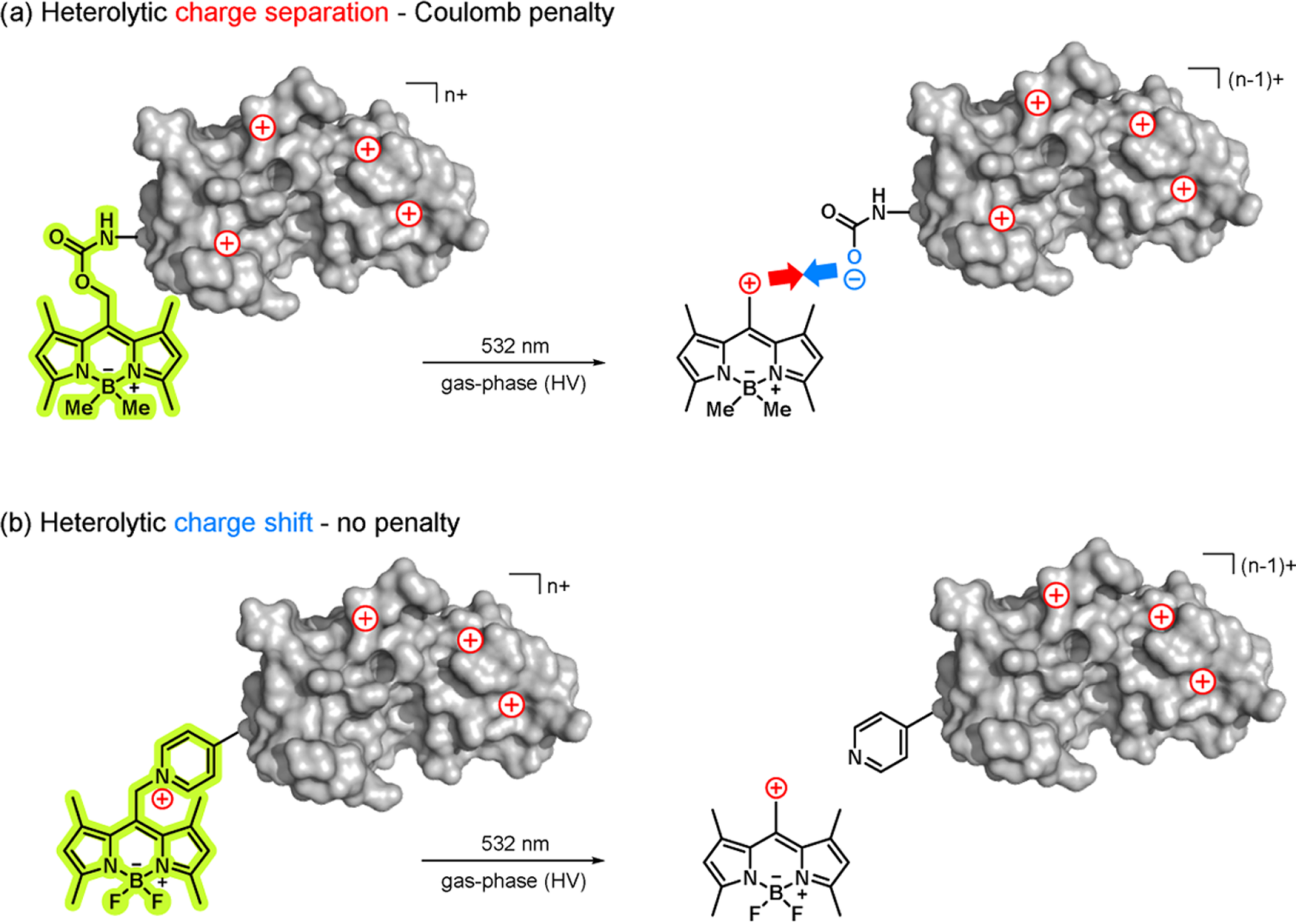
Concept of uncaging with green light in high vacuum via a charge shift. (a) The heterolytic cleavage of the carbamate linker leads to charge separation accompanied by a Coulomb penalty. (b) Heterolysis of an onium motif (here: pyridinium) as the leaving group shifts the charge from the cargo with no Coulomb penalty.

Here, we have selected the bodipy chromophore because of its strong absorption of green light at 532 nm, the high uncaging quantum yields of reported derivatives in solution, and because of the possibility to tune their absorption spectrum by chemical modification.^66−70^ In polar solvents, bodipy photocages undergo photoinduced heterolysis, which changes the charge state of the “cargo” by one negative unit as shown in Figure 1a.^69^ Bodipy photocages are, therefore, promising candidates to control the charge of a biomolecular cargo.

**Figure 2:**
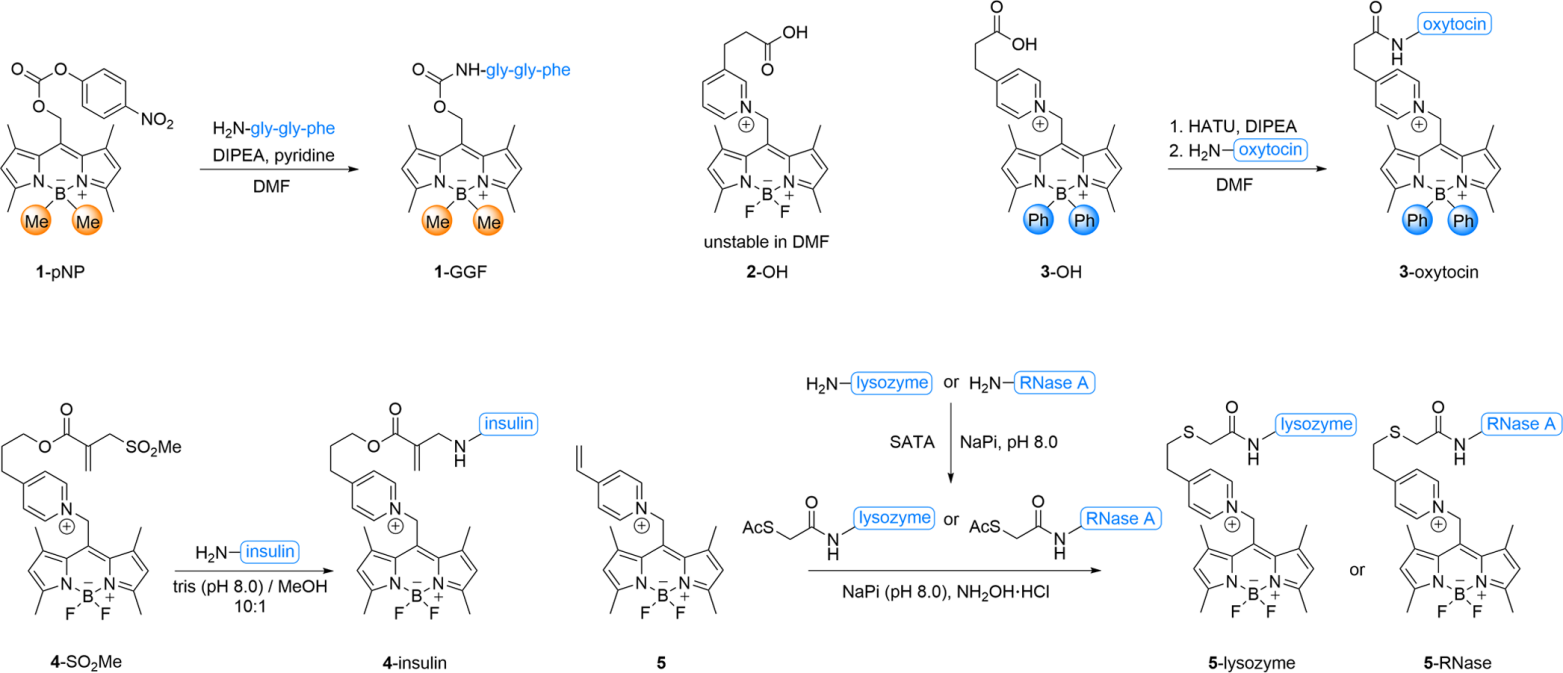
Compounds and synthetic routes of conjugates for gas-phase photocleavage experiments at 532 nm.

**Figure 3:**
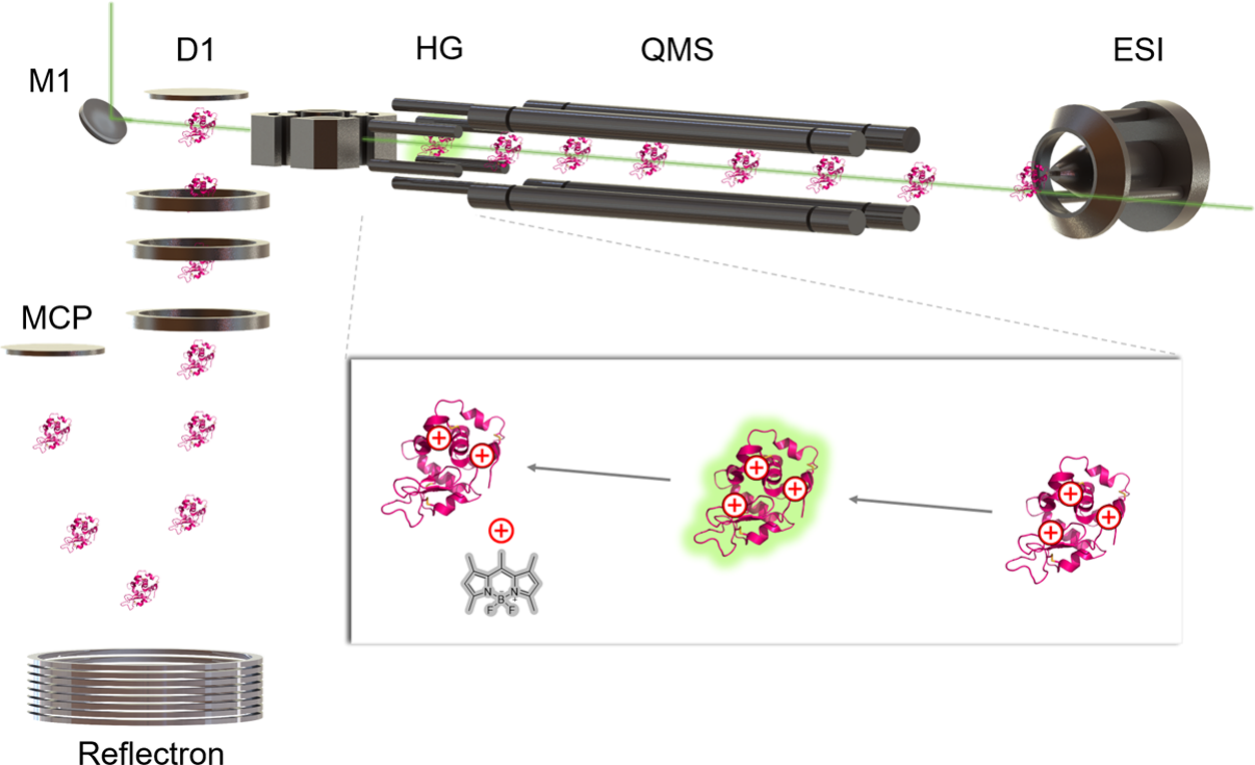
Photochemical charge reduction of proteins in the gas phase: ions are electro-sprayed (ESI) and mass-selected (QMS) before interacting with a counterpropagating intense green light pulse (10 ns, 532 nm, 60–300 mJ/cm^2^, guided into the setup by mirror M1) at the end of the hexapole guide (HG). The pulsed pusher (D1) sends the ions toward the reflectron of the TOF-mass spectrometer before detection by the multichannel plate detector (MCP). Laser pulse and pusher frequency are timed to reach a high signal intensity for photolyzed ions. Inset: a charge state of a labeled ion is mass-selected and interacts with a green laser pulse.

We synthesized functionalized tripeptide **1**-GGF (Figure 2) to test this hypothesis. The methyl substituents at boron are known to increase the uncaging quantum yield in solution.^67^ The photoactivation of **1**-GGF and of all subsequent molecules in high vacuum was then tested using a customized tandem mass spectrometer as shown in Figure 3. Electro-sprayed molecules are preselected in a quadrupole mass filter and analyzed in a time-of-flight mass spectrometry (TOF-MS) whose resolution suffices to identify the transfer of an individual H atom.^71^ Green pulsed laser light counterpropagating to the molecular beam induces photocleavage inside the hexapole guide (Figure 3). Parent and fragment ions are then analyzed by using the time-of-flight mass spectrometer.

**Figure 4:**
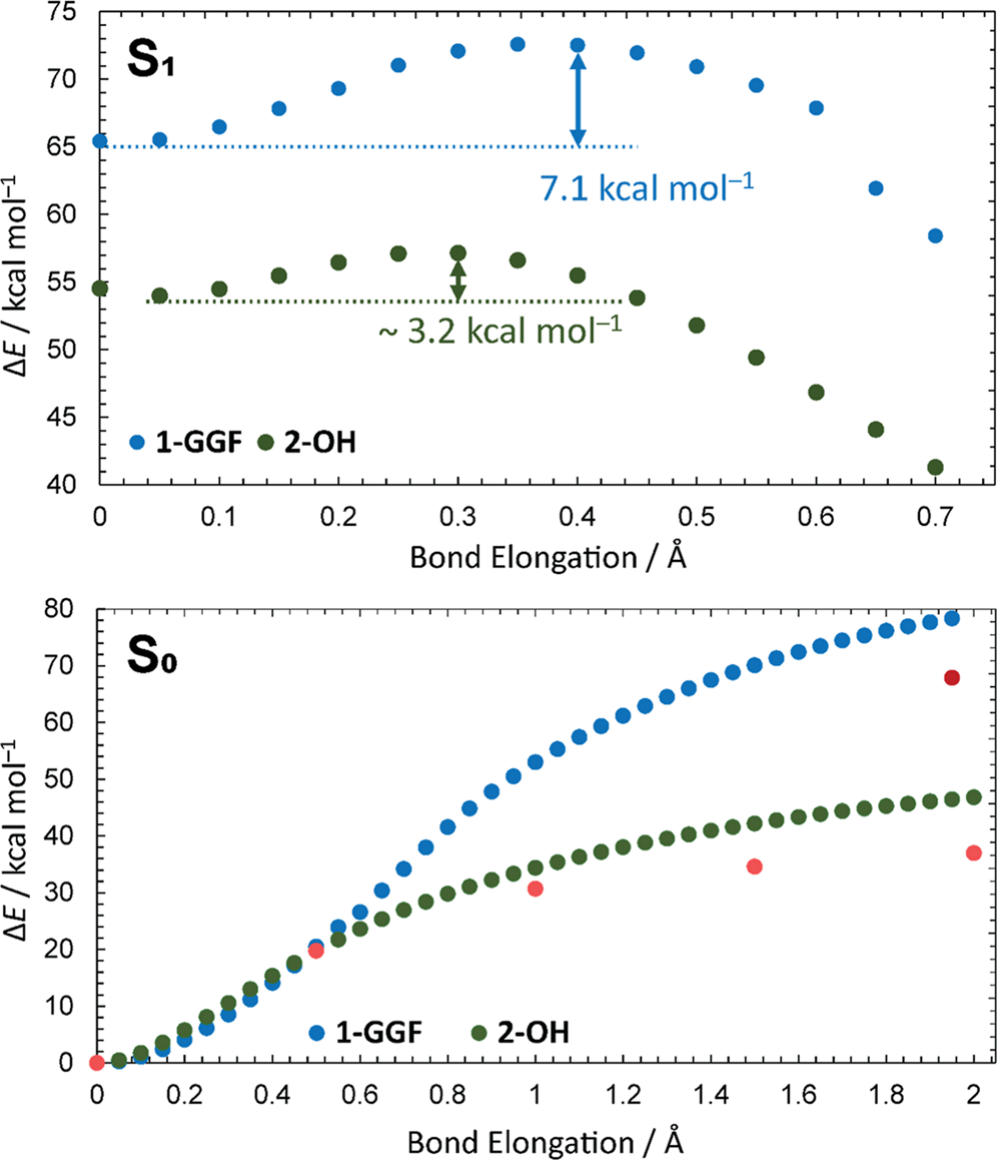
Calculated potential energy surface profiles along the bond dissociation coordinate in the S_1_ (top, TD-CAM-B3LYP) and S_0_ (bottom, B3LYP) states. The red and dark-red dots in the S_0_ profiles denote the energies obtained by broken-symmetry DFT (see the SI). See the Methods Section and SI for truncated structures used in the calculations.

The short lifetime of the excited singlet state (<5 ns), the small reorganization of the **1**-GGF chromophore (Stokes shift of ca. 150 meV), and the limited available excess energy in excited **1**-GGF in vacuum set a stringent limit on the barriers for photoprocesses in the S_1_ state. At the same time, the absence of heavy atoms limits the probability of intersystem crossing (ISC)^67−70^ to the longer-lived triplet excited state. Our quantum chemical calculations (Figures 4 and S27) showed that the carbamate dissociates from bodipy preferentially by homolysis with an energy barrier that appears insurmountable within the given excited-state lifetime.^76,77^ Consequently, **1**-GGF is expected to decay to the singlet ground state via fluorescence or internal conversion, redistributing the photon energy (2.33 eV) among its vibrational modes. This hot ground state could then dissociate (Tables S4–S11) into fragments during the flight time to the detector (<200 μs). However, we have observed no photocleavage of **1**-GGF in the negative mode, even though **1**-GGF displays only weak solvatochromism (Figure S24 and Tables S2 and S3) and should absorb well at 532 nm in high vacuum.

Collision-induced decomposition (CID) experiments with negative **1**-GGF ions and nitrogen gas showed a consistent set of fragments at >5 eV collision energies (Figure S13). The absence of fragments with two negative charges excludes heterolysis of the carbamate from bodipy, which we would have expected if the photoreaction in the gas phase paralleled that in a polar solvent. This is in accord with the calculated bond energies (Tables S6–S11). We observed three main peaks and assign the two most intense signals (Figures S11–S13) to secondary fragments formed in collisions with nitrogen already at a 5 eV collision energy but not after absorption of the green photon. Clearly, the threshold energies for dissociation in the S_0_ state must be higher than 2.33 eV (>54 kcal mol^–1^; see Figure 4 and the SI for further discussion).

The investigation of **1**-GGF provides a valuable design lesson for effective photocages in a high vacuum: contrary to the situation in polar solvents, heterolytic charge separation in high vacuum is unlikely due to a significant Coulomb energy penalty. However, photocages that allow for a shift of charges rather than their separation (Figure 1b) might change the charge of the “cargo” peptide because the heterolysis possesses lower activation barriers (Figure 4).

Based on this idea, we have synthesized photocage **2**–OH with a carboxylate-equipped pyridinium moiety. The onium motif (here: pyridinium; Figure 2) displays a high ion yield in the gas phase and improves the solubility of the construct in water. The fluorides at boron in **2**–OH provide a bathochromic shift of the absorption (λ_max_ = 525 nm) in nonpolar 1,4-dioxane, which is a good approximation of the value in vacuum (see the SI). The efficiency of heterolytic cleavage in photocages has been correlated with the p*K*_a_ of the conjugate acid of the released group.^59,60,67,78^ Since the p*K*_a_ of pyridinium is comparable to those of carboxylic acids, the dissociation of **2**–OH was expected to be feasible and to shift the charge from the pyridinium moiety to the bodipy fragment upon C–N bond heterolysis while avoiding charge separation.

However, the irradiation of **2**–OH in polar MeOH or acetonitrile with green light led only to minimal bleaching of the sample, even after 60 min of irradiation. We measured the excited-state lifetimes and fluorescence quantum yields of **2**–OH in MeOH and in less polar CH_2_Cl_2_ and compared them to pyrromethene 546 with a structurally identical chromophore as a reference compound (Table 1). Unlike pyrromethene 546, **2**–OH required two exponentials to fit its fluorescence decay data. The relative weight of the delayed fluorescence and the fluorescence quantum yield increased with a decreasing solvent polarity. The data thus point to a pseudoreversible formation of a nonemissive state (Scheme S1), which we assigned to a competing charge-transfer process that hampers efficient photolysis (Figures S18 and S19).^79−81^ Note that the absence of any photolysis of **2**–OH suggests that no triplet state is formed due to the nonorthogonal arrangement of the bodipy chromophore and the pyridinium moiety unlike in a related bodipy dyad.^79^

**Table 1:** Fluorescence Lifetimes and Fluorescence Quantum Yields of **2**–OH and Pyrromethene 546 as a Reference Compound

	**2-OH**^a^	reference
τ_f_ /ns in MeOH^b^	1.50 (82%), 5.58 (18%)^c^	6.38^d^
τ_f_ /ns in CH_2_Cl_2_^b^	1.86 (44%), 5.44 (56%)^c^	5.90^d^
Φ_f_ in MeOH	15.8%^e^	91.8%^f^
Φ_f_ in CH_2_Cl_2_	25.7%^e^	77.6%^f^

a With CF_3_CO_2_^–^ as the counteranion.

b Excitation wavelength of 472 nm.

c Detection wavelength of 550 nm.

d Detection wavelength of 515 nm.

e Excitation wavelength of 520 nm.

f Excitation wavelength of 480 nm.

**Figure 5:**
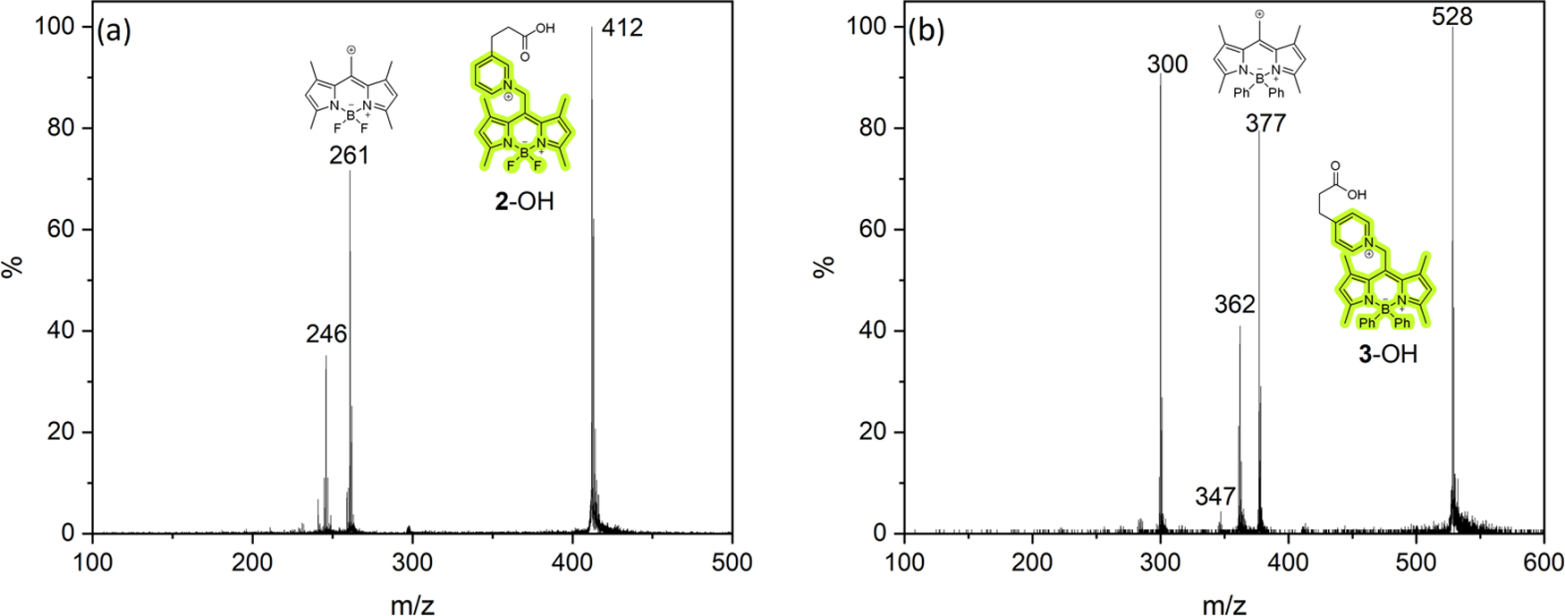
Gas-phase photolysis (532 nm) of bodipy-onium tags. Parent peaks were mass-selected before cleavage. Cleavage of (a) **2**–OH and (b) **3**–OH formed the corresponding bodipy cations and 3-(pyridyl)propionic acids.

Gratifyingly, the exposure of **2**–OH to pulsed green laser light in high vacuum resulted in clean heterolysis accompanied by the release of neutral pyridyl-propionic acid (Figure 5). This confirms our design strategy to replace a charge separation with a charge shift. These findings also underline that a photochemical channel that cannot compete with deactivation in a polar solvent can re-emerge in a high vacuum.

Construct **2**–OH shows good stability in protic solvents, which is desirable for protein couplings, but unstable in DMF or other solvents that are routinely used for peptide coupling (Figure S20). The stability in DMF could be improved by replacing the fluorine atoms at boron with phenyl groups to form **3**–OH, which was successfully coupled to the nonapeptide oxytocin (**3**-oxytocin). Alternative linkers (**4**-SO_2_Me,^82^**5**^83^) enabled convenient protein modification in aqueous solution to synthesize **4**-insulin, **5**-lysozyme, and **5**-RNase (Figure 2). For the modification of lysozyme and RNase A, a cysteine reactive linker was used. Since both proteins do not contain sulfhydryl groups at their surface, these were established by the reaction with commercially available N-succinimidyl S-acetylthioacetate (SATA). Substitution of fluorine in **2**–OH for methylgroups, which are known to improve the quantum cleavage yield in solution,^67^ led to a compound of low stability and this approach was not further pursued.

**Figure 6:**
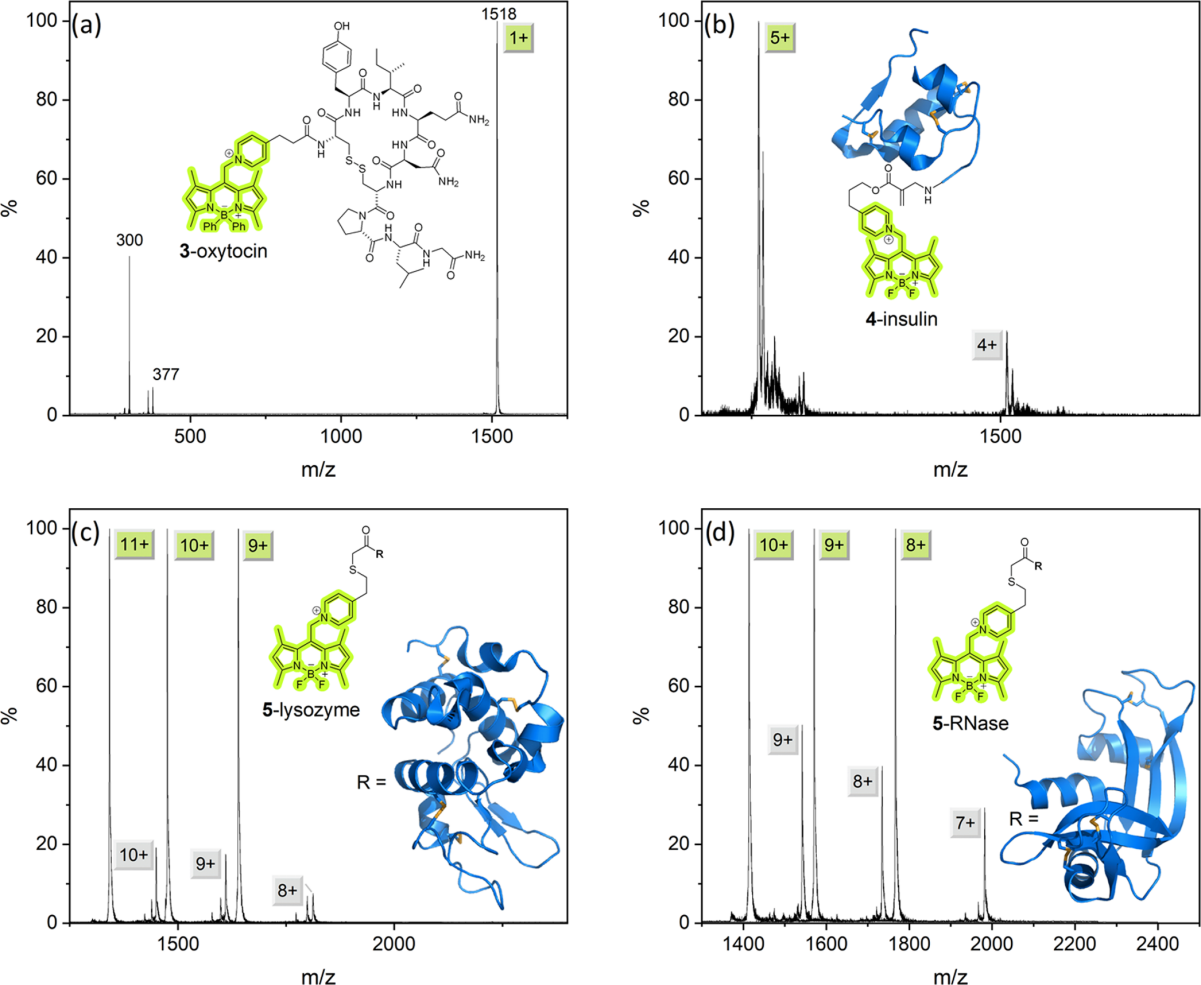
Photoactivated charge reduction of peptides and proteins in a high vacuum. In each experiment, a single parent ion peak (green label) is mass-selected before cleavage and charge-reduced by one unit. All spectra are normalized to their parent peak. (a) **3**-Oxytocin shows the same fragments as **3**–OH. (b) **4**-Insulin (5+), **(c) 5**-lysozyme (11+, 10+, 9+), and (d) **5**-RNase (10+, 9+, 8+). Panels (c, d) show the overlay of three independent and individually normalized experiments.

Figure 5b shows that the **3**–OH ion beam releases the bodipy fragment efficiently. Similarly, **3**-oxytocin releases the bodipy cation upon photoactivation (Figure 6a). In the cleavage of **2**–OH, **3**–OH, and **3**-oxytocin, additional fragments are observed, which can be formally derived from the bodipy cation after the loss of methyl or phenyl groups, respectively.^84,85^ Conveniently, these processes do not affect the quality of our design: the attached cargo always emerges cleanly from the reaction with a reduced charge state. This cannot be directly detected by mass spectrometry in the case of **2**–OH, **3**–OH, and **3**-oxytocin because the cargo leaves in a neutral state.

Comparing the photoactivation of **3**–OH and **3**-oxytocin indicates that the heterolytic cleavage efficiency decreases with an increasing number of amino acid residues in the cargo. This suggests that the absorbed photon energy is redistributed among the vibrational modes of the peptide, whose heat capacity increases with the number of amino acid residues.^86^

Nevertheless, tagged polypeptides and proteins as massive as insulin (51 AA residues), lysozyme (129 AA residues), or RNase A (124 AA residues) all show efficient cleavage under irradiation by 532 nm light (Figure 6b–d). Outside of photoelectron spectroscopy,^87^ this remarkable achievement represents the first reported photoinduced charge reduction processes of large polypeptides with visible light in high vacuum.

Our experiments show that **5**-RNase (124 AA residues; Figure 6d) cleaves with a yield comparable to that of **3**-oxytocin (9 AA residues; Figure 6a). This suggests that the presence of additional charge in the protein may affect the uncaging kinetics. Indeed, the cleavage efficiency decreases with a decreasing charge state of **5**-lysozyme and **5**-RNase (Figure 6c,d), an effect we observed previously with nitrobenzylether-tagged insulin.^71^ Two additional fragments are seen for **5**-lysozyme whose *m*/*z*-values correspond to the cleavage of the ethylene C–S bond in the linker or the tagged N-terminal lysine of lysozyme (Figure 6c).

## Conclusions

In conclusion, we have redesigned bodipy photocages to efficiently release their cargo, including large biomolecules, in high vacuum. The design enables a charge shift upon photoinduced heterolysis instead of the heterolytic charge separation observed in polar solvents. As a result, we can optically reduce the charge of large peptides, here demonstrated up to the size of lysozyme and RNase A (*m* = 14 kDa). It will be intriguing to extend our concept to more massive proteins and other visible-light-absorbing photocages and to release other cargo in high vacuum, such as small molecules, oligonucleotides, or saccharides. Our study thus opens a path to a plethora of new experiments that require a high degree of spatiotemporal control over molecular properties and dynamics in a high vacuum.

## Methods

### Gas-Phase Photocleavage Experiments

Experiments were performed using a customized Waters Q-TOF Ultima mass spectrometer. The electro-sprayed ions are collected by two ion funnels and guided through the vacuum system until they enter a quadrupole mass filter (QMS), where they are mass-selected. Two transfer hexapoles and a DC-guide are used to guide the ions to the TOF. A 45° mirror is placed in the high vacuum environment to align the laser (alternatively Edge Wave INNOSLAB or InnoLas SpitLight EVO I) collinearly and counterpropagating to the molecular ion beam.

### Molecular Beam Preparation

All peptides were sprayed using standard spray conditions. Ten 20 μM solutions of the peptides were prepared in either water or a mixture of water and acetonitrile under the addition of a small amount of formic acid. The solution was filled into a syringe pump and sprayed through a 125 μm capillary with a flow rate of 4 μL/min.

### Mass Filtering

The mass spectrometer was upgraded by MSVISION for a maximal mass range of *m*/*q* = 30′000 Da/e. This system still achieves atomic mass resolution in the region of interest, which we exploit to distinguish between heterolytic and homolytic processes and those accompanied by H-transfer. The mass filter operates at a base pressure of 1 × 10^–6^ mbar. The TOF-MS operates at 10^–7^ mbar. At pressures above 10^–6^ mbar, collision-induced cleavage was observed as an additional contribution to the signal.

### Photochemistry

The green laser pulse is generated by frequency doubling of a diode-pumped Nd:YAG laser. The light was spatially filtered to obtain a near-Gaussian intensity profile with a beam waist of *w*_0_ = 1.6 mm (1/*e*^2^-value). The laser power was regulated by combining a λ/2 waveplate with a polarizing beam splitter (PBS). For all experiments, the laser pulse energy was between 0.2 and 3 mJ, which is sufficient to allow efficient cleavage of all compounds. The laser beam entered through the top-lid of the TOF-MS, and it was directed by a 45° mirror toward the entrance hole of the customized ESI interface. The smallest beam constriction was the ion lens close to the TOF-MS instrument with a height of 1.7 mm.

### Photofragmentation Mass Spectra

Mass spectra were obtained by dividing the TOF-MS pusher frequency into a pulse train of 100 Hz, which triggered the laser emission and the Waters Q-TOF 4 GHz time-to-digital converter (TDC). This allowed us to adjust the delay between the laser and the TOF frequency to maximize the signal for depletion or fragmentation. It also ensured that every ion saw exactly one laser pulse. In these experiments, the delay between the laser pulse and the TOF extraction frequency was set to maximize the detection of all fragments of the cleavage process. To verify that all observed fragments are products of photochemistry, we compared the mass spectra with and without laser light. All mass spectra were stored as CSV files and analyzed by using the ORIGIN Pro 2021 software.

### Calculations

All calculations were performed with the Gaussian16 rev. C.01 suite of electronic structure programs.^51^ The geometries of the potential energy minima or transition states were optimized at the B3LYP/6-31G(d) level of theory. To reduce computational cost, the structures of **1**-GGF and **2**-GGF (see SI) were simplified by truncating the GGF-peptide residue to a methyl substituent. Compound **2**–OH was simplified by replacing the propanoic acid residue with a hydrogen atom.
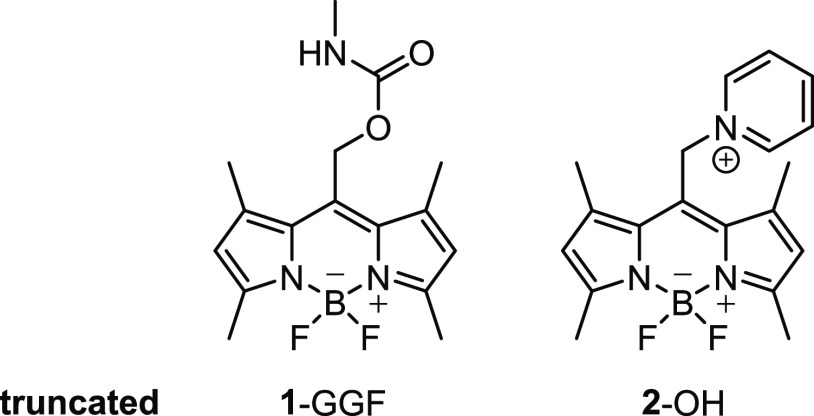


The nature of all stationary points was verified by frequency calculations. The nature of the transition states was verified by performing intrinsic reaction coordinate (IRC) calculations that connected the transition-state structure to the potential energy minima. Due to computed low imaginary force constants, the IRC paths did not always fully converge to the corresponding energy minima. Consequently, the geometry of the last point of the IRC path was optimized by calculating the full Hessian matrix in each optimization step to make sure that the optimization converged to the correct energy minimum. The wave function stability of the methylene-bodipy cations (see below) was tested. If an instability was found, the broken spin-symmetry (BS) Kohn–Sham wave functions were computed, in which the spatial symmetries of the α and β MOs are destroyed. Such a wave function was then used to optimize the geometry of the molecule. We denote such calculations here as BS-DFT. The single-point energies were then calculated with various DFT functionals and the cc-pVTZ basis set. The reported energies (at 0 K) given in kcal mol^–1^ represent the sum of the total electronic energy and the unscaled zero-point energy correction. The potential energy surface scans for the S_0_ ground state were performed on the B3LYP/6-31G(d) level of theory and for the S_1_ excited state were performed on the TD-CAM-B3LYP/6-31G(d) level of theory to avoid spurious intrusion of the charge-transfer states in **2**–OH as observed with the B3LYP functional. Both scans, for S_0_ and S_1_ states, were carried out along the C–O bond stretching coordinate in **1**-GGF and **2**-GGF (see SI) and of the C–N bond stretching coordinate in **2**–OH, while all other coordinates were relaxed in the optimization.

In the ground state, stretching of the bonds in the relaxed ground-state potential energy surface scans also leads to instability of the wave functions due to the diradicaloid nature of the ensuing methylene-bodipy. We tested the wave function stability in these cases and calculated the energy using the broken spin-symmetry Kohn–Sham wave functions to estimate the effect on the shape of the potential energy surface.

## Supporting Information Available

Synthesis of photocleavable tags and bioconjugation methods; analytical data for synthesized compounds; CID mass spectra; photostability experiments; fluorescence quantum yield and fluorescence lifetime determination; excitation spectra; experimental description for gas-phase photocleavage experiments; and DFT calculations (PDF)
