# Supplementary material for: Giving the Green Light to Photochemical Uncaging of Large Biomolecules in High Vacuum
Source: JACS Au. 2023 Oct 16;3(10):2790–9. doi: 10.1021/jacsau.3c00351 (PMC10598566; doi:10.1021/jacsau.3c00351)
Supplement: Supplementary file 1 — au3c00351_si_001.pdf [file au3c00351_si_001.pdf]

Supporting Information

Giving the green light to photochemical uncaging of large biomolecules in high vacuum

Yong Hua[1], Marcel Strauss[2], Sergey Fisher[3], Martin F. X. Mauser[2], Pierre Manchet[2], Martina Smacchia[2], Philipp Geyer[2], Armin Shayeghi[2], Michael Pfeffer[1], Tim Henri Eggenweiler[1], Steven Daly[4], Jan Commandeur[4], Marcel Mayor[1,5,6], Markus Arndt*[2], Tomáš Šolomek*[3], Valentin Köhler*[1]

[1]Department of Chemistry, University of Basel, St. Johannsring 19, CH-4056 Basel, Switzerland

[2]Vienna Faculty of Physics, University of Vienna, VDSP & VCQ, Boltzmanngasse 5, A-1090 Vienna

[3]Van't Hoff Institute for Molecular Sciences (HIMS), University of Amsterdam, PO Box 94157, 1090 GD Amsterdam, The Netherlands

[4]MS Vision, Televisieweg 40, 1322 AM Almere, The Netherlands

[5]Institute for Nanotechnology (INT), Karlsruhe Institute of Technology (KIT), P.O. Box 3640, DE-76021 Karlsruhe Eggenstein-Leopoldshafen, Germany

[6]Lehn Institute of Functional Materials, School of Chemistry, Sun Yat-Sen University, Guangzhou 510274, P. R. China

Correspondence should be addressed to: Markus Arndt, Tomáš Šolomek and Valentin Köhler

*markus.arndt@univie.ac.at

*t.solomek@uva.nl

*valentin.koehler@unibas.ch

Table of Contents

1. Synthesis - materials and methods 4
   - Preparation of **1**-pNP.[2] 5
   - Preparation of **1**-GGF 5
   - Preparation of bodipy-pyridinium **2**-OH 6
   - Preparation of **3**-OH 6
   - Preparation of **3**-oxytocin 7
   - Preparation of **S7** 8
   - Preparation of **4**-insulin 9
     - Experiments to determine the site of modification in **4-insulin** 10
   - Preparation of **5** 13
   - Preparation of a **5**-GS 13
   - Preparation of **5**-lysozyme and **5**-RNase 14
2. Comment on synthesis and design considerations 16
3. CID-measurements 16
   - **1**-GGF 16
   - **2**-OH 20
4. Photostability of **2**-OH in solution 22
5. Stability of **2**-OH and **3**-OH in DMF 24
6. Excited state lifetimes and fluorescence quantum yields 24
   - Fluorescence spectra of **2**-OH and the reference compound 27
   - Fluorescence lifetime measurements of **2**-OH and the reference in compound 28
   - Fluorescence quantum yield determination of **2**-OH and the reference compound 29
   - Excitation spectra of **2**-OH 29
7. UV-Vis Spectra 30
   - UV-Vis absorption of **1**-pNP and **2**-OH in solvents of different relative permittivity 30
   - UV-Vis absorption spectra of the compounds in MeOH 31
8. Gas phase photocleavage experiments 32
   - General set-up 32
   - Molecular beam preparation 32
   - Mass filtering 32
   - Photochemistry 33
   - Photofragmentation mass spectra 33
9. Calculations 34
   - General Remarks 34
   - Results 35

10. NMR Spectra of compounds 61

11. References 69

1. Synthesis

Chemicals were purchased from Sigma Aldrich, Fluorochem, Novabiochem or Bachem and used as received unless otherwise noted.

NMR experiments were performed at 25°C on Bruker Avance III NMR spectrometers operating at 600 or 500 MHz proton frequency. NMR spectrometers were equipped with inverse dual channel, broadband probe heads with z-gradients. ^{13}C shifts were determined by 1D or 2D (HMBC and HMQC/HSQC) NMR experiments. Spectra were referenced to residual non-deuterated solvent peaks of the NMR solvent (^{1}H NMR) or the signal of the deuterated solvent (^{13}C NMR). Abbreviations for multiplicities in quotation marks refer to pseudo-splitting patterns, i.e., as they appear in the ^{1}H-NMR spectra.

ESI-HRMS experiments were conducted with a Bruker maXis 4G.

LC-MS analyses were performed with a Shimadzu LC-MS 2020 system. The machines were additionally equipped with PDA detectors. LC-MS Shimadzu 2020: Column: Agilent Zorbax RR Eclipse, XDB-C18, 4.6 × 75 mm, 3.5 µm. Solvents were A (water : acetonitrile : formic acid=97% : 3% : 0.1%) and B (acetonitrile: water: formic acid = 95%: 5% : 0.1%). The flow rate was set to 1 mL/min and the temperature to 40°C. Method 1: 0 min – 0% B; 1 min – 5% B; 9 min – 95% B; 11 min – 95. Method 2: 0 min – 0% B; 1 min – 2% B; 20 min 40% B; ; 22 min – 95% B; 26 min – 95%.

Preparative HPLC separations were carried out with a Water Prep LC 4000 System and the following columns: (Column A) Dr. Maisch, Reprospher, 100 C18-DE, 40 × 150 mm, 5 µm, 100Å; (Column B) Dr. Maisch, Reprospher, 300 C4-DE, 40 × 150 mm, 5 µm, 300Å. (A) water/acetonitrile/TFA = 97 : 3 : 0.1; (B) acetonitrile/TFA = 99.9 : 0.1. Flow rates were between 10 and 20 mL/min. Extinction coefficients and isolated yields for compounds purified by preparative HPLC do not consider possible residual TFA after lyophilization.

UV-Spectra were measured on a Cary 50 at room temperature.

Bodipy derivatives **S1**, **S2** and **S3** were prepared following a published method.[1]

Preparation of **1**-pNP.[2]

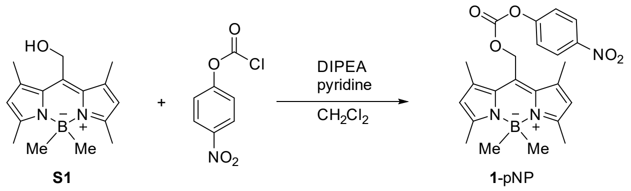

1-pNP was prepared in analogy to a published procedure.[3] To a solution of 4-nitrophenyl chloroformate (68 mg, 0.34 mmol) in dry CH_2Cl_2 (5 mL) was added pyridine (27 µL, 0.33 mmol) under a nitrogen atmosphere. The formed suspension was added dropwise to a cooled solution (ice-bath) of **S1** (27 mg, 0.10 mmol) and DIPEA (60 µL, 0.34 mmol) in dry CH_2Cl_2 (5 mL) in the dark. The mixture was allowed to warm to room temperature and stirring continued for 4 hours. Volatiles were removed under reduced pressure and the residue was purified by column chromatography over silica with petroleum ether/ethyl acetate (5:1) starting with pure petroleum ether. Product **1**-pNP was obtained as a red solid in 76% yield. ^{1}H NMR (500 MHz, $CDCl_3$, the methyl groups at boron were not detected) δ 8.29 ('d', *J* = 9.1 Hz, 2H), 7.41 ('d', *J* = 9.2 Hz, 2H), 6.12 (d, *J* = 1.2 Hz, 2H), 5.61 (s, 2H), 2.48 (s, 6H), 2.46 (s, 6H), 0.20 (s, 6H). ^{13}C NMR (126 MHz, $CDCl_3$) δ 155.51, 153.98, 152.45, 145.67, 137.07, 131.31, 131.03, 125.53, 123.30, 121.76, 62.79, 16.79, 16.22.

Preparation of **1**-GGF

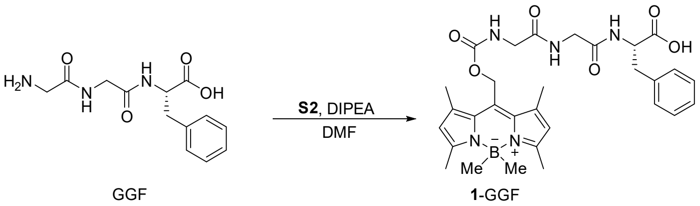

GGF (25 mg, 90 µmol) and DIPEA (16 µL, 90 µmol) were dissolved in DMF (1 mL). This solution was transferred to a solution of **1**-pNP (26 mg, 60 µmol) and pyridine (7.2 µL, 90 µmol) in DMF (2 mL). The mixture was stirred for 4h at room temperature, concentrated under reduced pressure and the crude product purified by column chromatography over silica with CH_2Cl_2/MeOH (10:1) starting with pure CH_2Cl_2, to obtain **1**-GGF as a red solid in 24% yield. HRMS-ESI m/z: [M]- calcd 574.2842, found 574.2826. ^{1}H NMR (600 MHz, DMSO-d_6) δ 8.20 (d, *J* = 8.1 Hz, 1H), 8.03 (t, *J* = 5.8 Hz, 1H), 7.65 (t, *J* = 6.2 Hz, 1H).δ 7.27 (t, *J* = 7.5 Hz, 2H), 7.20 (dd, *J* = 12.8, 7.1 Hz, 3H), 6.19 (s, 2H), 5.23 (s, 2H), 4.42 (td, *J* = 8.5, 5.1 Hz, 1H), 3.78 – 3.61 (m, 4H), 3.04 (dd, *J* = 13.7, 5.0 Hz, 1H), 2.87 (dd, *J* = 13.7, 9.0 Hz, 1H), 2.40 (s, 6H), 2.35 (s, 6H), 0.11 (s, 6H). ^{13}C NMR (151 MHz, DMSO-d_6) δ 172.80, 169.07, 168.62, 156.31, 152.48, 137.50, 137.47, 134.54, 130.68, 129.16, 128.25, 126.50, 122.64, 57.98, 53.50, 43.39, 41.58, 36.82, 29.03, 16.24, 15.54, 10.12.

UV-VIS (MeOH): $\varepsilon_{512nm(max)}$ = 6.5 × 10^4 $M^{-1}cm^{-1}$, ε_{532nm} = 0.68 × 10^4 $M^{-1}cm^{-1}$.

Preparation of bodipy-pyridinium **2**-OH

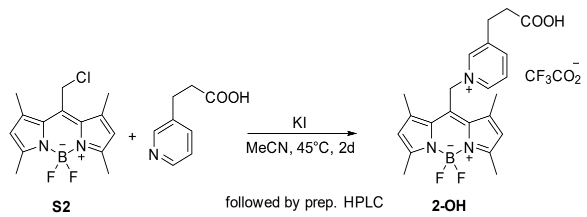

A mixture of 3-(3-pyridyl)propionic acid (30 mg, 0.20 mmol), a spatula tip of KI and **S2** (30 mg, 0.10 mmol) in MeCN (5 mL) was stirred at 45°C for 48 hours. The reaction mixture was filtered and directly injected into the preparative HPLC, (column A). After lyophilization the product **2**-OH was obtained as a red solid (5.6 mg, 11%). HRMS-ESI m/z: $[M]^+$ calcd. 412.2006, found 412.2009. LC-MS method 1: T_R = 4.5 min.

^{1}H NMR (600 MHz, MeOD-d_4) δ 9.06 (s, 1H), 8.79 (d, *J* = 6.2 Hz, 1H), 8.61 (d, *J* = 8.0 Hz, 1H), 8.06 (dd, *J* = 8.0, 6.2 Hz, 1H), 6.27 (s, 2H), 6.16 (s, 2H), 3.16 (t, *J* = 6.9 Hz, 2H), 2.80 (t, *J* = 6.9 Hz, 2H), 2.53 (s, 6H), 2.17 (s, 6H). ^{13}C NMR (151 MHz, MeOD-d_4) δ 175.1, 160.2, 148.3, 145.5, 145.3, 143.4, 141.9, 134.1, 130.0, 127.6, 124.6, 56.4, 34.3, 28.6, 15.6, 14.8.

UV-VIS (MeOH): $\varepsilon_{525nm(max)}$ = 6.0 × 10^4 $M^{-1}cm^{-1}$, ε_{532nm} = 3.9 × 10^4 $M^{-1}cm^{-1}$.

Preparation of **3**-OH

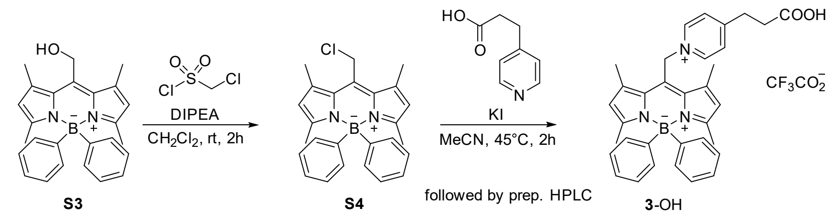

Chloromethanesulfonylchloride (18 µL, 30 mg, 0.20 mmol) was added to a solution of **S3** (39 mg, 0.99 mmol) and DIPEA (35 µL, 0.20 mmol) in CH_2Cl_2 (10 mL) at 0°C and the reaction mixture was allowed to warm to room temperature and stirred for 2 hours. Volatiles were subsequently removed under reduced pressure and the residue purified by column chromatography over silica (CH_2Cl_2:PE = 0:100 - 20:80) to obtain **S4** as a dark red solid (36 mg, yield: 87%). The product was used directly for the following step.

3-(Pyridin-4-yl)-propionic acid (12 mg, 79 µmol), a spatula tip of KI and **S4** (16 mg, 39 µmol) were reacted in CH_3CN (5 mL) at 45°C for 2 hours. The mixture was subsequently purified by preparative HPLC (column A) and the product **3**-OH was obtained as a dark red solid (17 mg, 68%).

HRMS-ESI m/z: $[M]^+$ calcd. 528.2823, found 528.2827. LC-MS method 1: T_R = 6.2 min.

^1H NMR (500 MHz, MeOD-d_4) δ 8.33 ('d', *J* = 6.8 Hz, 2H), 7.74 ('d', *J* = 7.0 Hz, 2H), 7.31 – 6.94 (m, 10H), 6.23 (d, *J* = 1.1 Hz, 2H), 6.16 (s, 2H), 3.17 (t, *J* = 7.0 Hz, 2H), 2.80 (t, *J* = 7.0 Hz, 2H), 2.26 (d, *J* = 0.9 Hz, 6H), 1.77 (s, 6H). ^{13}C NMR (151 MHz, MeOD-d_4) δ 175.0, 165.0, 159.5, 143.8, 140.7, 135.0, 129.7, 129.6, 128.8, 127.6, 125.7, 56.2, 33.4, 31.4, 17.6, 16.0.

UV-VIS (MeOH): $\varepsilon_{525nm(max)} = 5.7 \times 10^4\ M^{-1}cm^{-1}$, $\varepsilon_{532nm} = 3.9 \times 10^4\ M^{-1}cm^{-1}$

Preparation of **3**-oxytocin

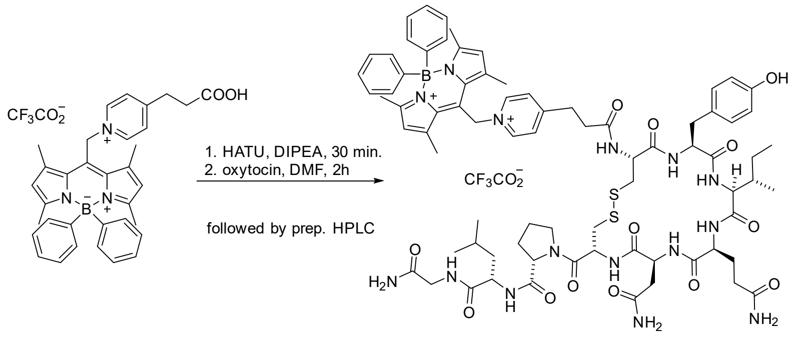

3-OH **3**-oxytocin

3-OH (5.3 mg, 8.3 µmol), HATU (7.6 mg, 20 µmol) and DIPEA (6.9 µL, 40 µmol) were dissolved in DMF (2.0 mL) and stirred for 30 min at room temperature. A solution of oxytocin (15 mg, 15 µmol, 1.5 equiv) in DMF (1 mL) was added and stirring continued for 2h at room temperature. Subsequently, water (1.5ml) was added and the mixture purified by preparative HPLC (column A) to obtain **4**-oxytocin as a dark red solid (7.6 mg, 56 %).

HRMS-ESI m/z: $[M+Na]^{2+}$ calcd 769.8490, found 769.8491. LC-MS method 1: T_R = 5.7 min.

^1H NMR (600 MHz, MeOD-d_4) δ 8.62 (d, *J* = 8.6 Hz, 1H), 8.58 (d, *J* = 5.8 Hz, 1H), 8.48 (d, *J* = 8.9 Hz, 1H), 8.41 (s, 1H), δ 8.32 ('d', *J* = 6.5 Hz, 2H), 8.27 (d, *J* = 6.6 Hz, 1H), 8.15 (d, *J* = 7.7 Hz, 2H), 8.00 (d, *J* = 7.4 Hz, 1H), 7.67 ('d', *J* = 6.5 Hz, 2H), 7.39 – 6.91 (m, 12H), 6.68 (d, *J* = 8.4 Hz, 2H), 6.23 (s, 2H), 6.16 (s, 2H), 5.13 (q, *J* = 7.7 Hz, 1H), 4.73 (dddd, *J* = 32.5, 12.5, 10.0, 5.8 Hz, 3H), 4.46 (dd, *J* = 8.6, 3.1 Hz, 1H), 4.25 (dt, *J* = 9.2, 6.0 Hz, 1H), 4.09 (dt, *J* = 10.0, 5.4 Hz, 1H), 3.93 – 3.86 (m, 2H), 3.79 – 3.65 (m, 3H), 3.32 (s, 1H), 3.30 – 3.27 (m, 1H), 3.22 – 3.07 (m, 3H), 3.04 – 2.97 (m, 1H), 2.92 – 2.83 (m, 2H), 2.77 (dd, *J* = 15.9, 7.6 Hz, 1H), 2.67 (dd, *J* = 13.9, 6.4 Hz, 1H), 2.59 (h, *J* = 8.2 Hz, 2H), 2.35 (tq, *J* = 15.8, 7.6, 6.7 Hz, 2H), 2.25 (s, 7H), 2.19 – 2.06 (m, 2H), 2.01 (tq, *J* = 11.2, 6.7, 5.4 Hz, 3H), 1.87 (q, *J* = 9.0, 6.7 Hz, 1H), 1.76 (s, 6H), 1.73 – 1.64 (m, 3H), 1.58 (ddt, *J* = 11.4, 7.5, 4.6 Hz, 1H), 1.21 (ddd, *J* = 16.3, 14.3, 7.6 Hz, 1H), 0.99 (d, *J* = 6.8 Hz, 3H), 0.96 (dt, *J* = 7.5, 4.2 Hz, 6H), 0.92 (d, *J* = 6.2 Hz, 3H).

Preparation of **S7**

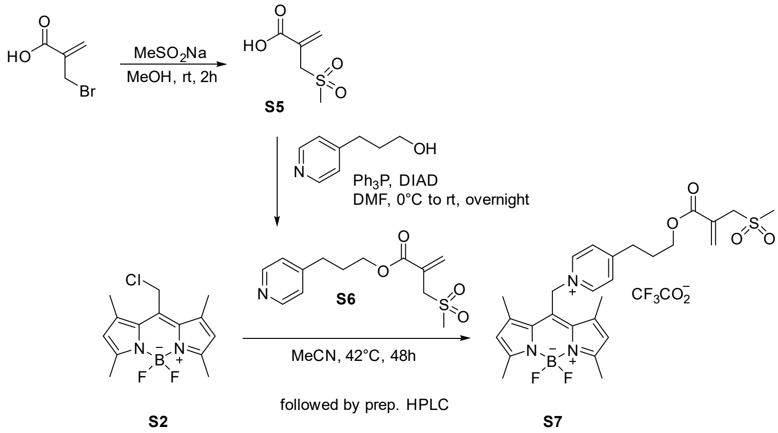

To a solution of 2-(bromomethyl)acrylic acid (330 mg, 2.00 mmol) in methanol (5 mL) was added sodium methanesulfinate (224 mg, 2.20 mmol) in portions over 10 min at room temperature. The mixture was stirred at room temperature for 2h and subsequently concentrated under reduced pressure. Rapid filtration over silica (CH_2Cl_2:MeOH = 10:0 - 10:1), yielded **S5** as a white solid, which was used directly in the next step.

S5 (164 mg, 1.00 mmol), 3-(pyridin-4-yl)propan-1-ol (137 mg, 1.00 mmol), and triphenylphosphine (315 mg, 1.20 mmol, 1.20 eq.) were dissolved in dry DMF (5 mL) and the mixture cooled to 0°C. Subsequently, DIAD (294 µl, 1.50 mmol, 1.50 eq.) was added dropwise over 15 min and the reaction was allowed to warm to room temperature and stirring was continued overnight. Water (30 mL) was added and the mixture extracted with EtOAc (3 × 20 mL). The combined organic phases were washed with brine (3 ×), dried over anhydrous sodium sulfate and volatiles removed under reduced pressure. Column chromatography over silica (CH_2Cl_2 : MeOH=10:0 - 10:1) yielded **S6** as a colorless oil (138 mg, 49%). HRMS-ESI m/z: $[M+H]^+$ calcd. 284.0951, found 284.0954.

^{1}H NMR (500 MHz, $CDCl_3$) δ 8.50 (d, *J* = 6.1 Hz, 2H), 7.13 (d, *J* = 6.1 Hz, 2H), 6.56 (s, 1H), 6.13 (s, 1H), 4.24 (t, *J* = 6.4 Hz, 2H), 4.05 – 4.04 (m, 2H), 2.90 (s, 3H), 2.73 (dd, *J* = 8.5, 6.9 Hz, 2H), 2.16 – 1.97 (m, 2H). ^{13}C NMR (126 MHz, $CDCl_3$) δ 165.3, 150.1, 149.9, 134.0, 129.1, 124.0, 64.9, 56.6, 40.7, 31.7, 29.1.

A mixture of **S6** (51 mg, 0.18 mmol), a spatula tip of KI and **S2** (59 mg, 0.2 mmol) in CH_3CN (10 mL) was stirred at 42°C for 2 days. The formed solid was separated by centrifugation and purified by preparative HPLC (column A) to yield **S7** as a red solid (16 mg, 14%).

HRMS-ESI m/z: $[M]^+$ calcd. 544.2252, found 544.2256. LC-MS method 1: T_R = 4.9 min.

^{1}H NMR (600 MHz, MeOD-d_4) δ 8.87 (d, *J* = 6.5 Hz, 2H), 8.06 (d, *J* = 6.5 Hz, 2H), 6.51 (s, 1H), 6.28 (s, 2H), 6.12 (s, 2H), 6.06 (d, *J* = 1.1 Hz, 1H), 4.29 (t, *J* = 5.9 Hz, 2H), 4.23 (s, 2H), 3.12 (t, *J* = 7.6 Hz, 2H), 2.98 (s,

3H), 2.53 (s, 6H), 2.21 – 2.16 (m, 8H). ^{13}C NMR (151 MHz, MeOD-d$_4$) δ 166.6, 165.8, 160.2, 144.2, 143.4, 134.0, 131.1, 130.3, 127.9, 124.6, 65.2, 56.9, 55.7, 40.4, 33.2, 29.3, 15.7, 14.9, 14.8.

UV-VIS (MeOH): $\varepsilon_{525nm(max)}$ = 6.7 × 10^4 M^{-1}cm^{-1}, ε_{532nm}= 4.1 × 10^4 M^{-1}cm^{-1}

Preparation of **4**-insulin

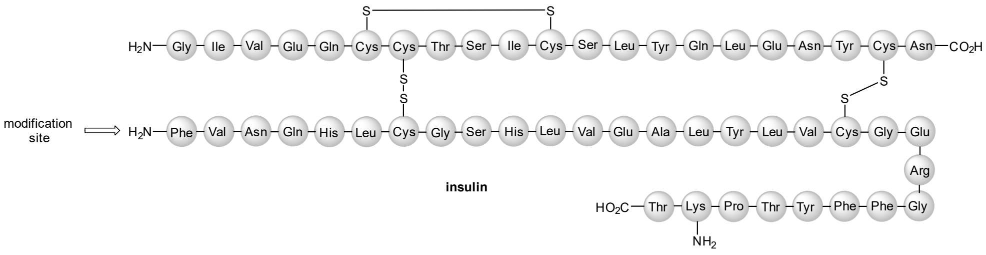

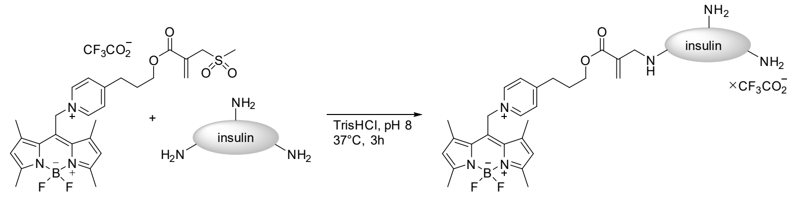

A solution of **S7** in MeOH (400 µL, 2.68 µmol, 6.7 mM) and insulin (7.8 mg, 1.3 µmol.) was added to TrisHCl buffer (4 mL, 20 mM, pH 8) and the resulting mixture stirred for 2 h at 37°C. After purification by preparative HPLC (column B, main fractions are collected at 525 nm) a red solid was obtained (1.2 mg, 15% without considering TFA). The cleavage of the disulfide bonds with TCEP and a tryptic digest followed by LC-MS analysis revealed that the isolated product was modified at the N-terminus of the B-chain.

HRMS-ESI m/z: calcd. [M]$^{4+}$ 1568.4753, [M]$^{5+}$ 1254.9817, [M]$^{6+}$ 1045.9859, [M]$^{7+}$ 896.7033, found [M]$^{4+}$ 1568.4781, [M]$^{5+}$ 1254.9841, [M]$^{6+}$ 1045.9885, [M]$^{7+}$ 896.7040. LC-MS method 1: T_R = 4.4 min.

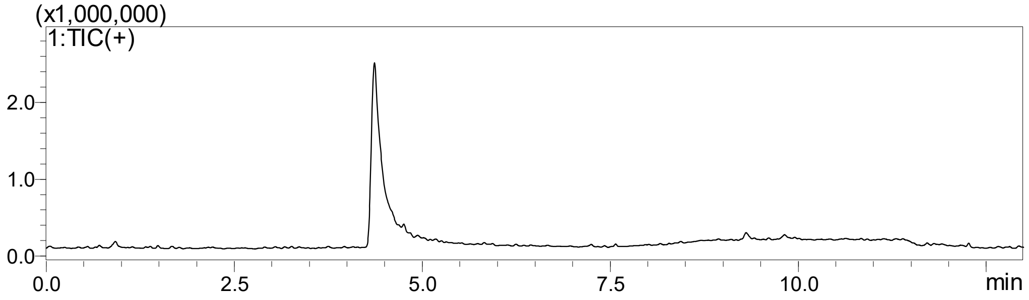

*Figure S1. LC-MS trace of **4**-insulin (total ion count, ESI(+) scan mode m/z 400 - 1850)*

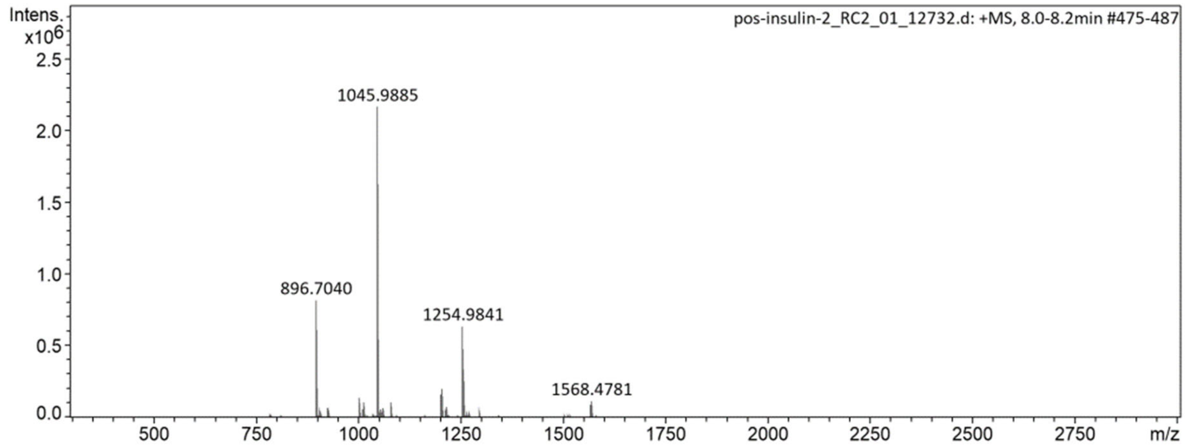

*Figure S2. High resolution mass spectrum (Bruker-Maxis II) extracted from an LC-MS run of **4**-insulin between 8.0 min and 8.2 min.*

Experiments to determine the site of modification in **4-insulin**:

Insulin has three reactive amino groups, i.e., at the N-termini of the A and B chain and at the side chain of Lys29 of the B-chain. LC-MS clearly showed that only singly modified product was formed. In order to elucidate the site or possible sites of modification two experiments were conducted with **4**-insulin: i) Separation of the A and B-chain by reduction of the disulfide bonds with TCEP and ii) tryptic digest of the modified insulin. The formed mixtures were analysed by LC-MS (Shimadzu LC-MS 2020). Only fragments that are consistent with the modification of the N-terminal phenylalanine of the B-chain were observed.

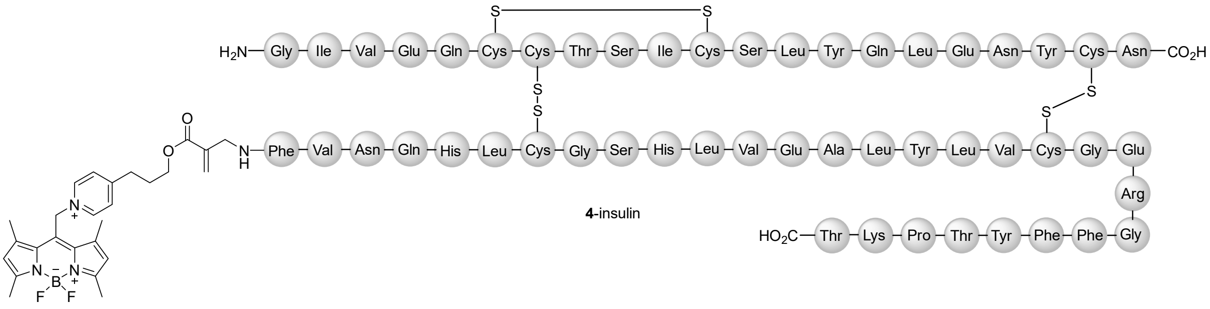

Reduction with TCEP

4-insulin (0.13 mg, 0.020 µmol) was dissolved in Tris buffer (0.50 mL, 20 mM, pH 8.0). To this solution was added TCEP-HCl (5.0 µL, 10 eq, 40 mM in Tris buffer (20 mM, pH 8.0). The resulting mixture was incubated at 37 °C for 1h. A sample analyzed by LC-MS (method 2) showed the following diagnostic fragments with the indicated m/z-values:

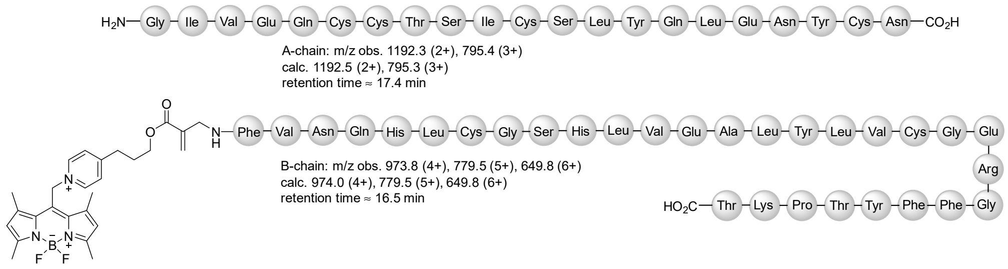

No signal corresponding to the mass of the modified A-chain was detected.

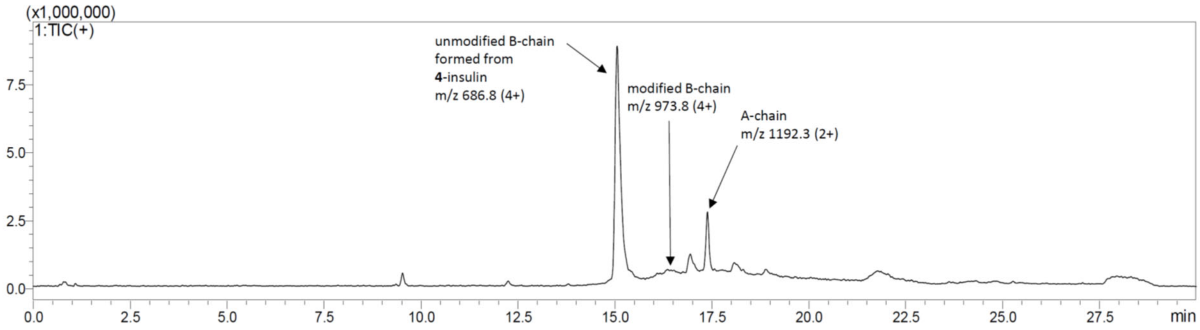

Figure S3. Chromatogram (total ion count, ESI (+), scan-mode m/z 400-1650). Peaks have been assigned based on the observed extracted mass spectra.

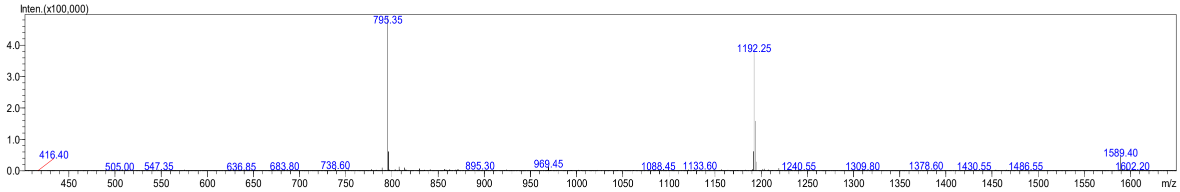

Figure S4. Mass spectrum extracted at 17.3-17.5 min of the LC-MS chromatogram showing diagnostic signals for the unmodified A-chain.

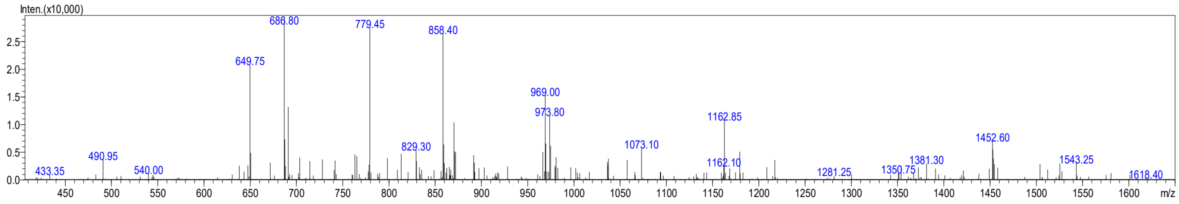

Figure S5. Mass spectrum extracted at 16.0-17.0 min of the LC-MS chromatogram showing diagnostic signals for the modified B-chain.

Trypsin digestion

The title compound (0.13 mg, 0.020 µmol) was dissolved in TrisHCl buffer (0.50 mL, 20 mM, pH 8.0). To this solution was added trypsin in TrisHCl buffer (1.3 mg/ml, 5 µL, 1:20 w/w, trypsin to peptide). The resulting mixture was incubated at 37 °C overnight. Analysis by LCMS (Shimadzu LCMS 2020, following diagnostic fragments with the indicated m/z-values:

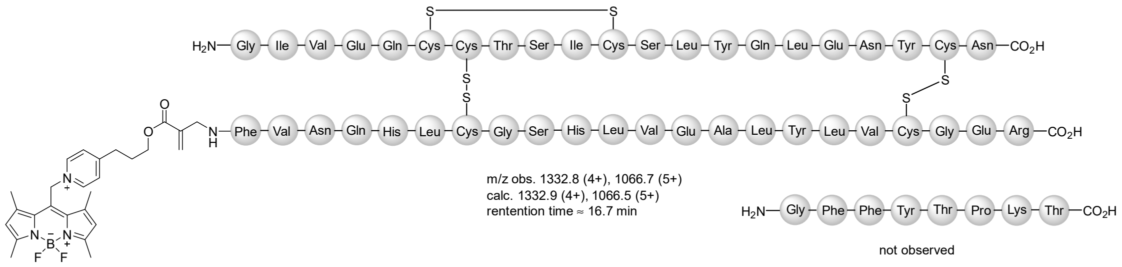

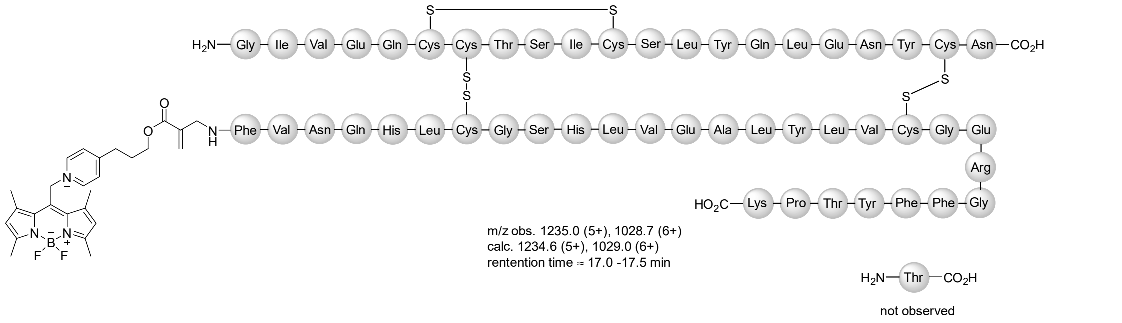

No signals indicating modification of Lys29 of the B-chain were observed.

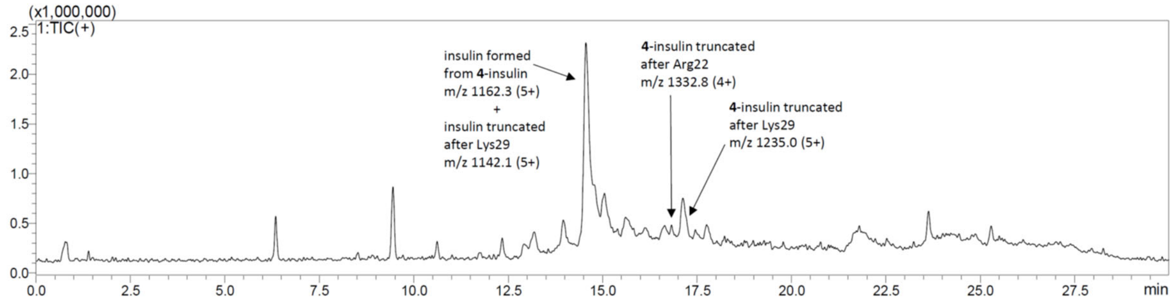

Figure S6. Chromatogram (total ion count, ESI (+), scan-mode m/z 400-1650). Peaks have been assigned based on the extracted mass spectra.

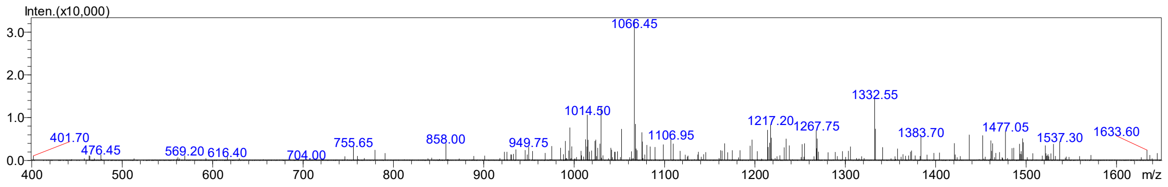

Figure S7. Mass spectrum extracted at 16.5-17.0 min of the LC-MS chromatogram showing diagnostic signals for the tagged fragment truncated after Arg22 of the B-chain.

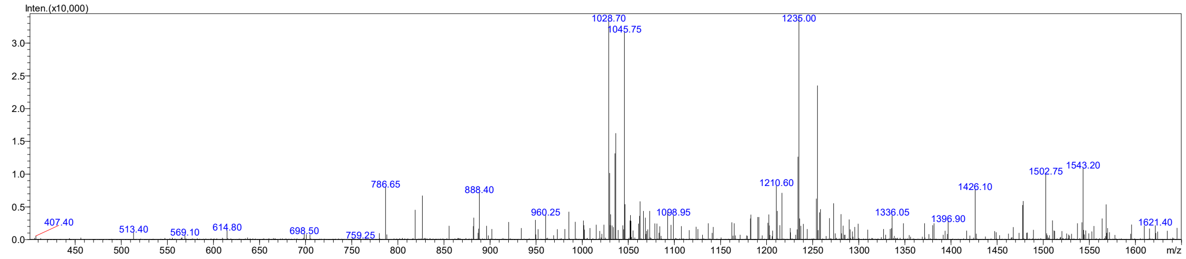

Figure S8. Mass spectrum extracted at 16.0-18.5 min of the LC-MS chromatogram showing diagnostic signals for the tagged fragment truncated after Lys29 of the B-chain.

Preparation of **5**

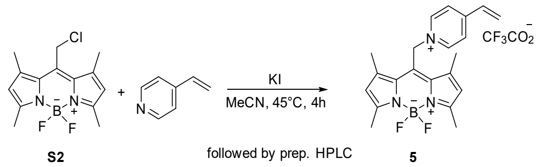

A mixture of 4-vinylpyridine (21 mg, 0.20 mmol), a spatula tip of KI and **S2** (30 mg, 0.10 mmol) in MeCN (5 mL) was stirred at 45°C for 4 hours. The reaction mixture was filtered and directly injected into the preparative HPLC, (column A). After lyophilization the product was obtained as a red solid (9.8 mg, 20%). The compound had low stability und was used directly in the next step. HRMS-ESI m/z: $[M]^+$ calcd. 366.1948, found 366.1956. LC-MS method 1: T_R = 4.8 min.

Preparation of a **5**-GS

In order to characterize a conjugation product in more detail, **5** was reacted with GSH.

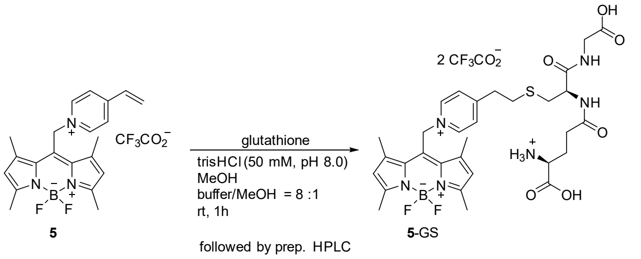

A solution of **5** (4.8 mg, 10 µmol) in 1 ml MeOH and glutathione (4.6 mg, 15 µmol.) was added to TrisHCl buffer (8 mL, 50 mM, pH 8.0) and the resulting mixture stirred for 1 h at rt. Purified by preparative HPLC (column A) and after lyophilization a red solid was obtained (6.1 mg, 68%). HRMS-ESI m/z: [M]+ calcd. 673.2791, found 673.2786. LC-MS method 1: T_R = 4.4 min.

^{1}H NMR (600 MHz, CD_3OD) δ 8.90 (d, *J* = 6.4 Hz, 2H), 8.09 (d, *J* = 6.3 Hz, 2H), 6.28 (s, 2H), 6.14 (s, 2H), 4.58 (dd, *J* = 8.5, 5.4 Hz, 1H), 4.11 – 3.84 (m, 3H), 3.28 (t, *J* = 7.2 Hz, 2H), 3.02 (ddd, *J* = 15.6, 13.4, 6.0 Hz, 3H), 2.80 (dd, *J* = 14.0, 8.5 Hz, 1H), 2.61 – 2.53 (m, 8H), 2.19 (s, 8H). ^{13}C NMR (151 MHz, CD_3OD)

δ 174.50, 173.00, 172.69, 171.94, 164.36, 160.23, 144.11, 143.30, 134.00, 130.53, 127.83, 124.58, 55.83, 54.15, 53.79, 41.78, 36.31, 34.40, 32.44, 31.76, 27.18, 15.61, 14.84.

UV-VIS (MeOH): $\varepsilon_{525nm(max)} = 6.0 \times 10^4$ $M^{-1}cm^{-1}$, $\varepsilon_{532nm} = 3.7 \times 10^4$ $M^{-1}cm^{-1}$

Preparation of **5**-lysozyme and **5**-RNase

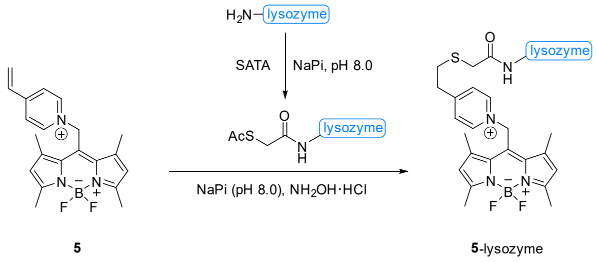

Egg white lysozyme or bovine pancreatic RNase A (14 mg, 1.0 µmol), respectively, was dissolved in NaPi buffer (8 mL, 50 mM, pH 8.0) and S-Acetylthioglycolic acid N-hydroxysuccinimide ester (0.35 mg, 1.5 µmol) was added in MeCN (0.5 mL). The mixture was stirred for 2 h at rt. Hydroxylamine hydrochloride (10 mg, 0.15 mmol) and **5** (1.4 mg, 3.0 µmol) in a small amount of MeOH (ca. 0.5 mL) were added, and stirring continued for 1 h. The mixture was purified by preparative HPLC (column B, detection at 525 nm) and two main fractions were collected consisting of single and double tagged proteins, respectively. After lyophilization red solids were obtained.

Single tagged lysozyme: 3.3 mg. HRMS-ESI m/z: [M]+ calcd. 14744 Da, found 14745 Da.

Double tagged lysozyme: 2.5 mg. HRMS-ESI m/z: [M]+ calcd. 15183 Da, found 15183 Da.

Single tagged RNase A: 2.8 mg. HRMS-ESI m/z: [M]+ calcd. 14121 Da, found 14121 Da.

Double tagged RNAse A: 1.8 mg. HRMS-ESI m/z: $[M]^+$ calcd. 14561 Da, found 14562 Da.

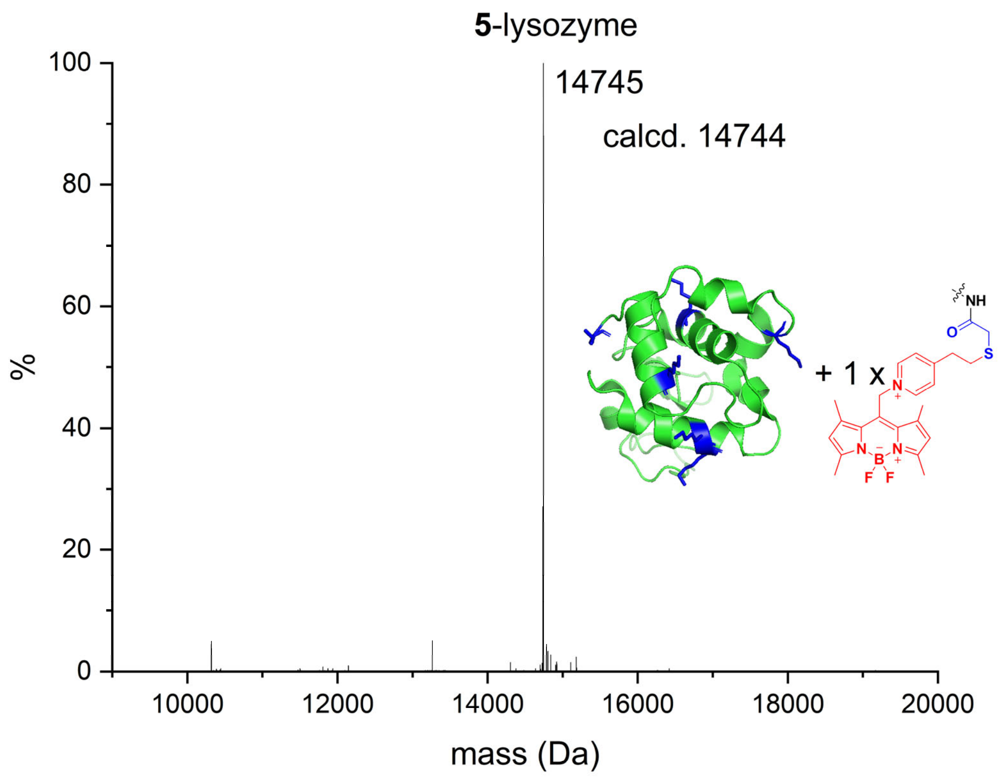

*Figure S9. Deconvoluted mass spectrum of **5**-lysozyme.*

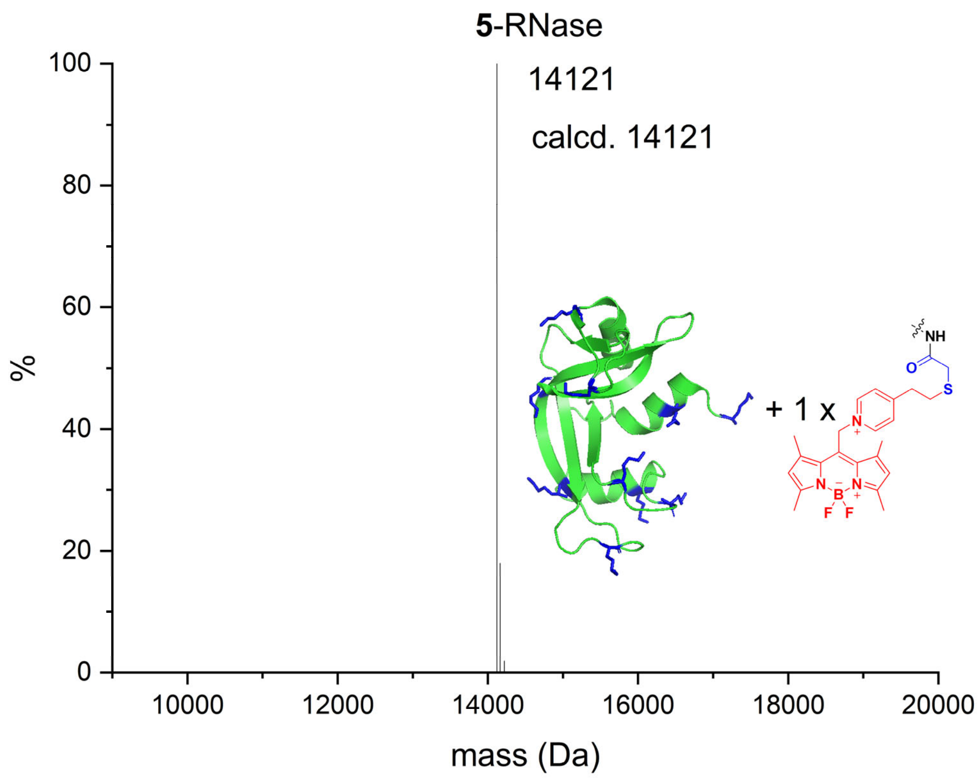

*Figure S10. Deconvoluted mass spectrum of **5**-RNase.*

2. Comment on synthesis and design considerations

Remarkably, **2**-OH cleaves efficiently in high vacuum without heavy atom substitution or methyl groups at boron, features that were introduced to improve cleavage yields in solution.[1] This simplifies the synthesis of the bodipy phototags, which require only one step from commercially available precursors followed by the reaction with the respective pyridine derivative. Construct **2**-OH shows good stability in protic solvents which is desirable for protein couplings but unstable in DMF or SOLVENT that are routinely used for peptide coupling, (Figure S20). The stability in DMF could be improved by replacing the fluorine atoms at boron with phenyl groups to form **3**-OH, which was successfully coupled to the nonapeptide oxytocin (**3**-oxytocin). Alternative linkers (**4**-SO_2Me[4], **5**[5]) enabled convenient protein modification in aqueous solution to synthesize **4**-insulin, **5**-lysozyme, and **5**-RNase.

3. CID-measurements

In order to investigate feasible thermal decomposition pathways, we conducted CID (collision-induced dissociation) experiments on **1**-GGF in negative mode and **2**-OH in positive mode. Experiments were conducted on a Bruker maXis 4G with N_2 as the collision gas.

1-GGF

In contrast to the gas-phase photocleavage experiments, collision-induced decomposition (CID) experiments with negative **1**-GGF ions and nitrogen as collision gas showed a consistent set of fragments, already at collision energies of 5–25 eV.

We observed fragments (Figures S11 - S13) corresponding to the peptide with a carbamic acid end group at the N-terminus at low relative intensity (322.1032; 0.4%). The dominant fragments can be derived from this peptide by decarboxylation (278.1145; 100%) or dehydration (304.0939; 75%). This series of fragments is most likely formed via a sigmatropic rearrangement, in which one H from the methyl group in the bodipy chromophore shifts to the carbamate linker of the peptide accompanied by synchronous cleavage of the bodipy-O bond. We calculated the transition state for this process (see Table S4), whose activation energy requires ~2.4 eV to occur. This can be achieved within the collision energies used in our CID experiments.

The heterolysis of the carbamate group was also observed and it became more pronounced at higher collision energies (>10 eV) than the sigmatropic rearrangement, yielding a bodipy-O^- fragment (269.1824) with ~10% relative intensity at 20 eV. Formally, positive and negative charges separate in

a heterolysis, a process that requires very high energies due to Coulomb attraction. Indeed, our calculations (Tables S6, S8, S10, S11) show clearly that any such heterolysis cannot compete with homolytic bond cleavage, either in the carbamate group or between the carbamate and bodipy. Therefore, we believe that the formation of this fragment is assisted by a nucleophilic attack of the pendant carboxylate at the C-terminus (Table S14). Consequently, the bond cleavage is triggered while the charge only formally shifts from one part of the molecule to the other resulting in the observed fragmentation.

Additionally, an unidentified fragment with an m/z value of 344.1409 (1.6%) was formed at higher collision energies (≥ 12.5 eV). As expected, no direct heterolysis between the bodipy-methylene carbon and the carbamate group was seen. Such a process would require charge separation and the formation of a doubly charged peptide fragment.

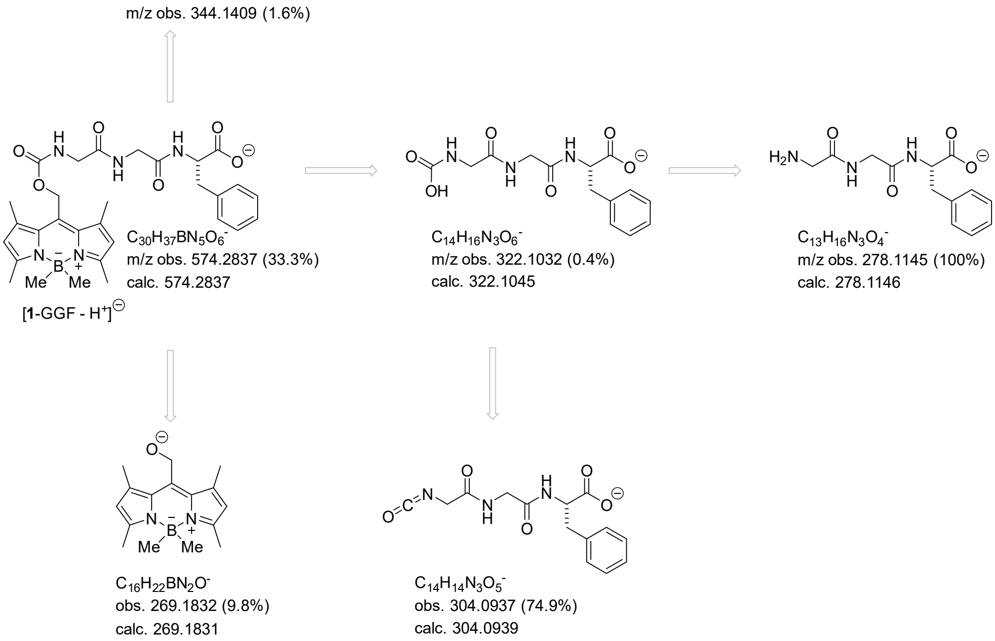

*Figure S11. Observed m/z values and tentative structures and fragmentation pathways for species observed in CID of **1**-GGF in the negative mode with N_2 as collision gas (20 eV). Relative abundance is displayed in brackets and calculated m/z values are displayed below the observed m/z values. Signals with a relative abundance <0.4% are not included.*

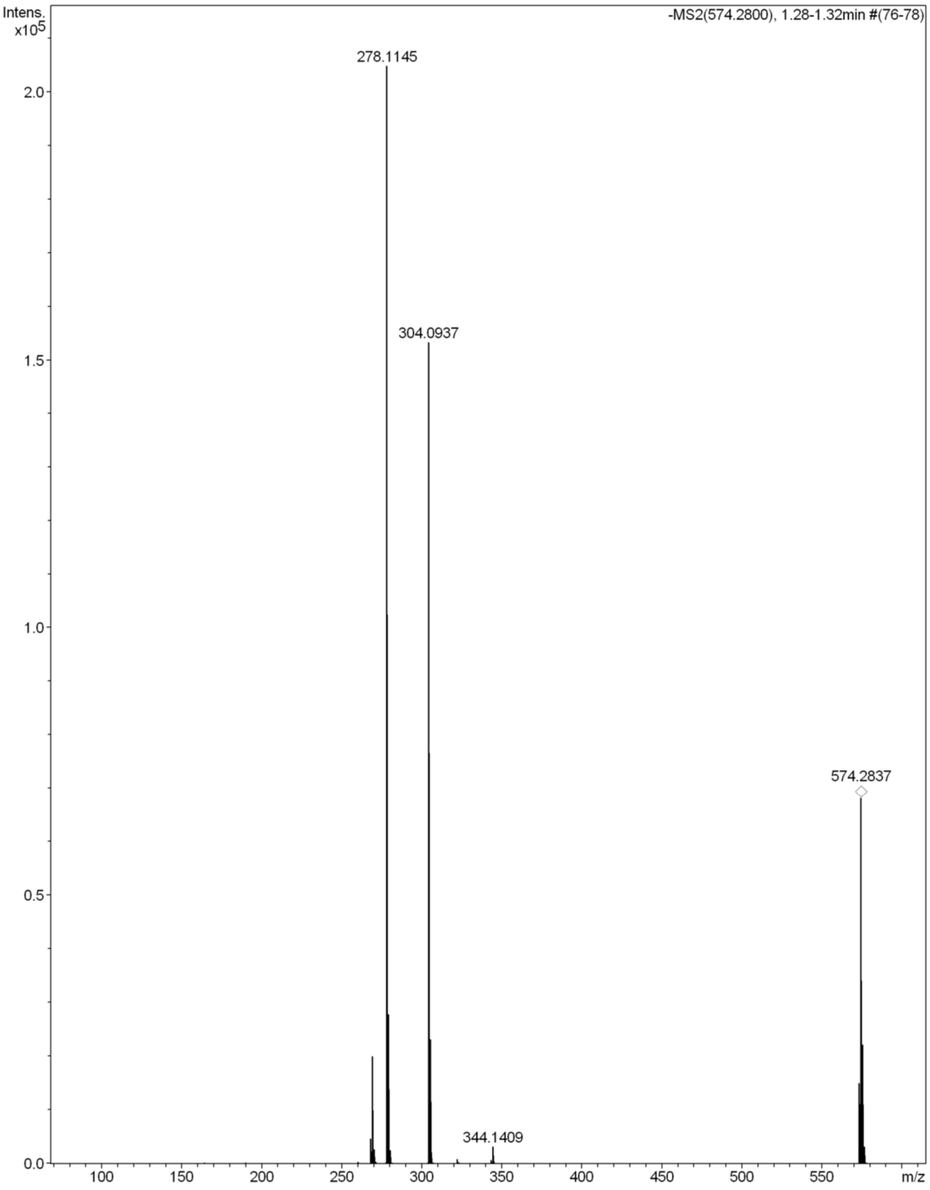

*Figure S12. MSMS of **1**-GGF, negative mode with N_2 as collision gas (20 eV).*

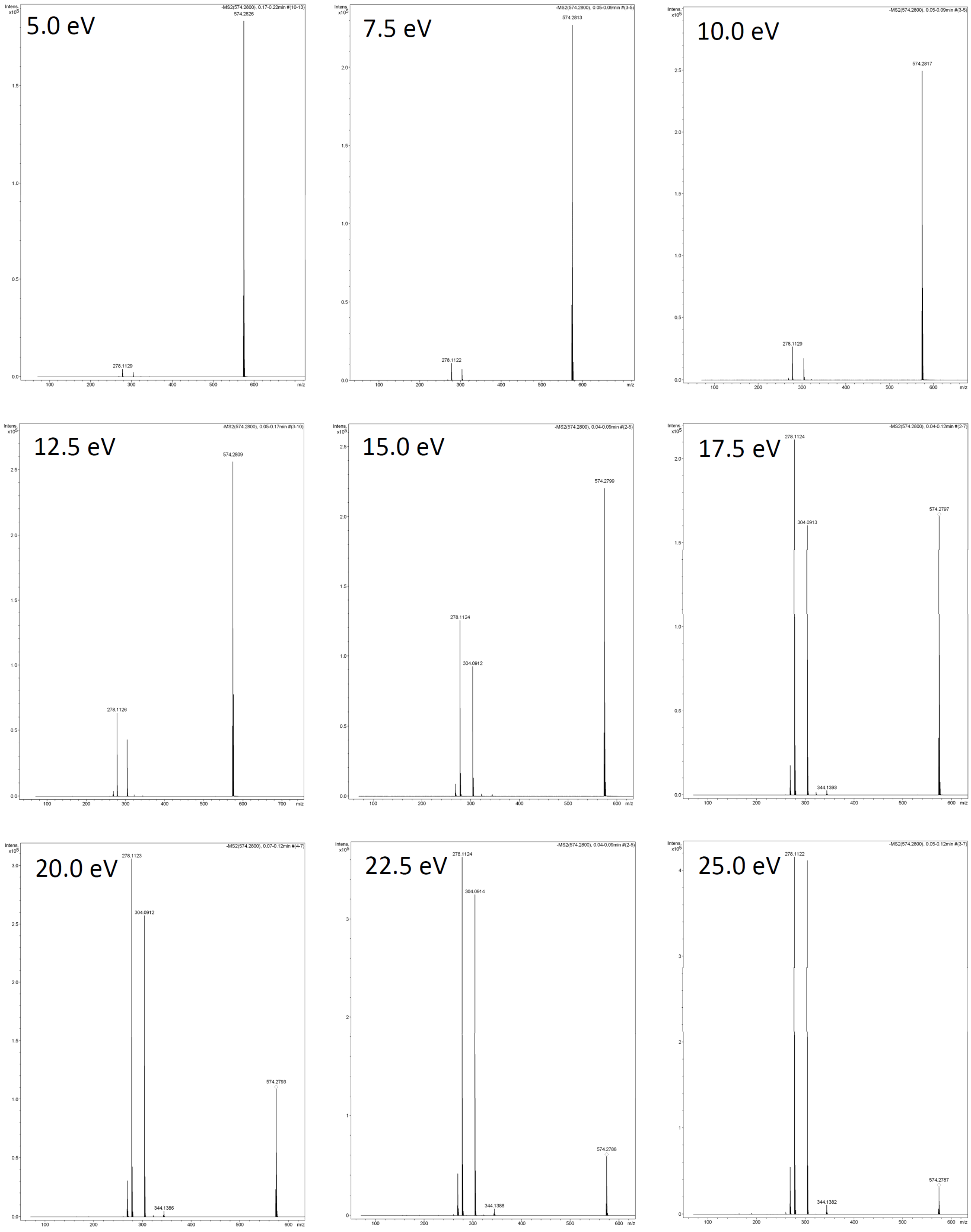

*Figure S13. MSMS of **1**-GGF, negative mode with N_2 as collision gas at collision energies from 2.5 eV to 25 eV.*

2-OH

In contrast to **1**-GGF, **2**-OH was cleaved cleanly by CID (N_2; positive ion mode) via charge-shifting heterolysis with the bodipy cation as the only observable fragment signal (100%) up to 24 eV collision energy Figures S14 - S16). At higher energies, secondary fragments corresponding to $CH_3^{\bullet}$-loss (30 eV; 246.1134; 0.9%) and HF-loss (36 eV; 241.1298; 1.4%) were additionally observed.

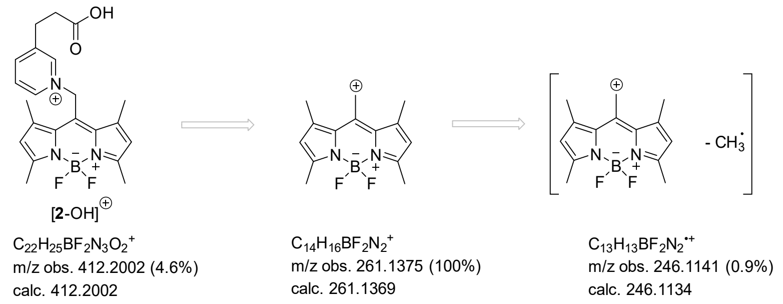

*Figure S14. Observed m/z values and structures and fragmentation pathway for species observed in CID of **2**-OH, positive mode with N_2 as collision gas (30 eV). Relative abundance is displayed in brackets and calculated m/z values are displayed below the observed values.*

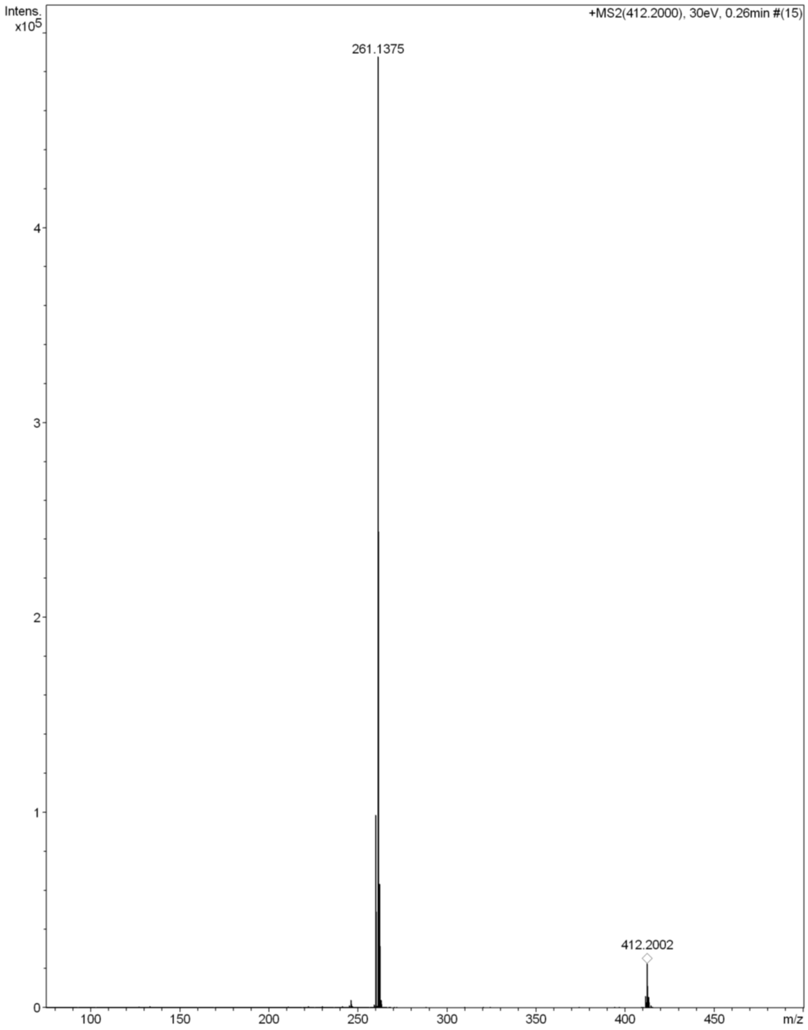

*Figure S15. MSMS of **2**-OH, positive mode with N_2 as collision gas (30 eV).*

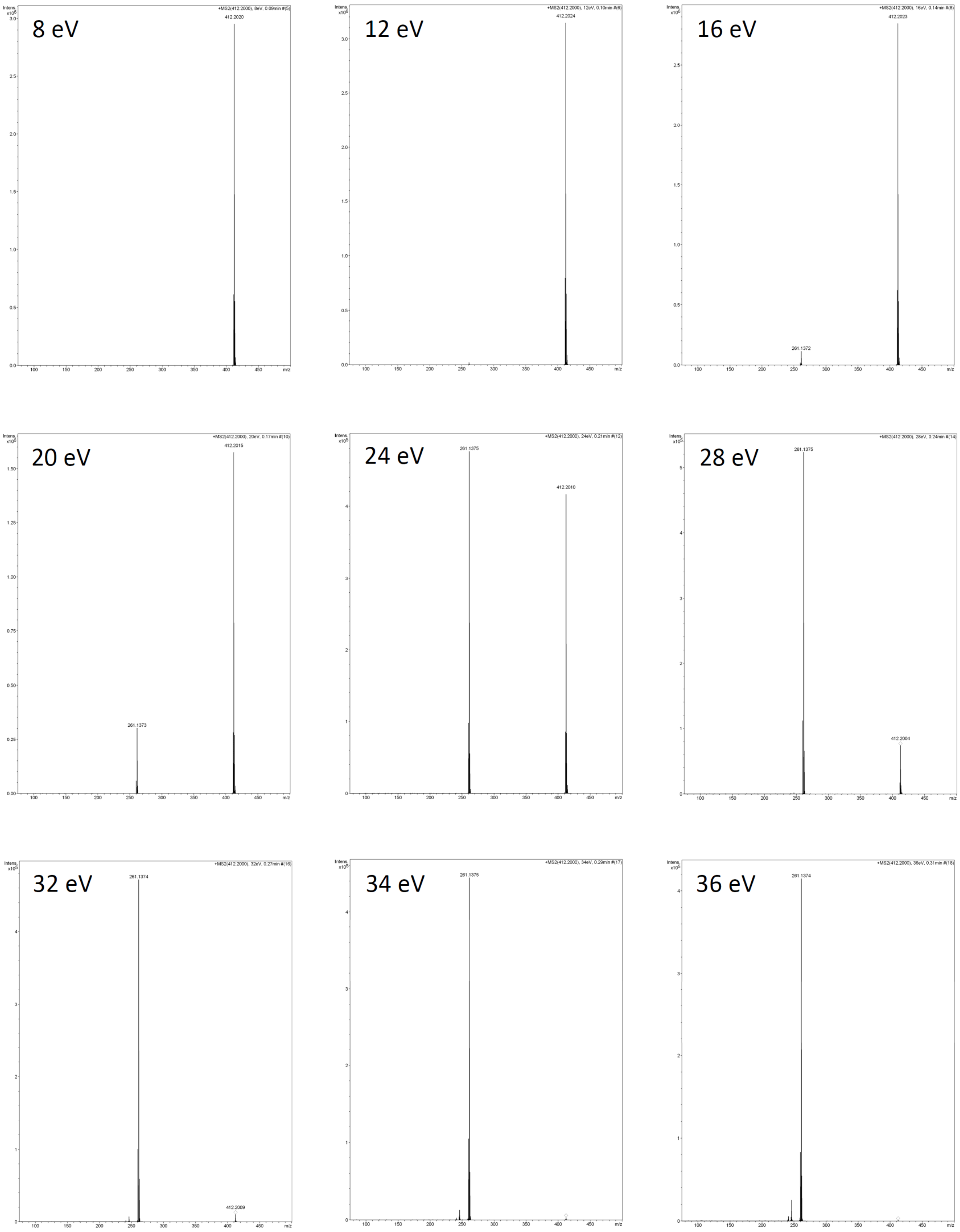

*Figure S16. MSMS of **2**-OH, positive mode with N_2 as collision gas at collision energies from 8 eV - 36 eV.*

4. Photostability of 2-OH in solution

The photostability of **2**-OH in solution (MeOH) was investigated by comparison to the stability of an established photocage (**S2**). Compounds **2**-OH and **S2**[1] have nearly identical absorption spectra with only a slight shift by 3 nm.

Samples of **2**-OH and **S2** (10 µM) were prepared by diluting stock solutions in MeOH (1 mL) in quartz-cuvettes with 10 mm path length for the t_0 measurement and immediately afterwards transferred to hplc-vials with screw caps. The vials were placed in a vial stand (Figure S17) where the vials were illuminated from the bottom with green LED-light (light source: ELGO LED stripes - BASIC - RGB) without stirring. The solutions were transferred back to the quartz cuvette at the indicated time points and a UV-spectrum (instrument: Cary 50) was taken before illumination in the HPLC vials was continued.

We observed a rapid shift (within seconds) in the absorption spectrum of **S2** (instrument: Cary 50), indicating a successful photoreaction, whereas the absorption spectrum of **2**-OH changed only marginally after prolonged irradiation (only a subtle change was observed within 60 min; Figure S18). Compound **S2** has a reported photocleavage quantum yield of 1.62 ± 0.11% in aerated MeOH, which suggests that **2**-OH is a very poor photocage in MeOH. This was further confirmed by LC-MS of the solution of **2**-OH after 60 min of irradiation (Figure S19). No visible changes in the chromatograms could be observed.

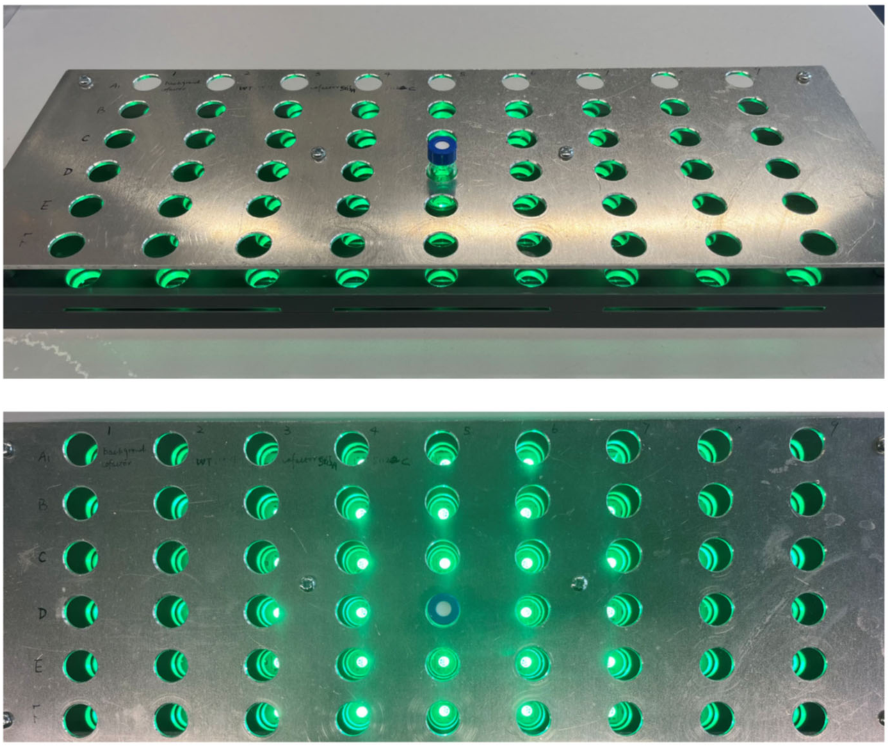

Figure S17. Instrumental set-up for photocleavage experiments in solution.

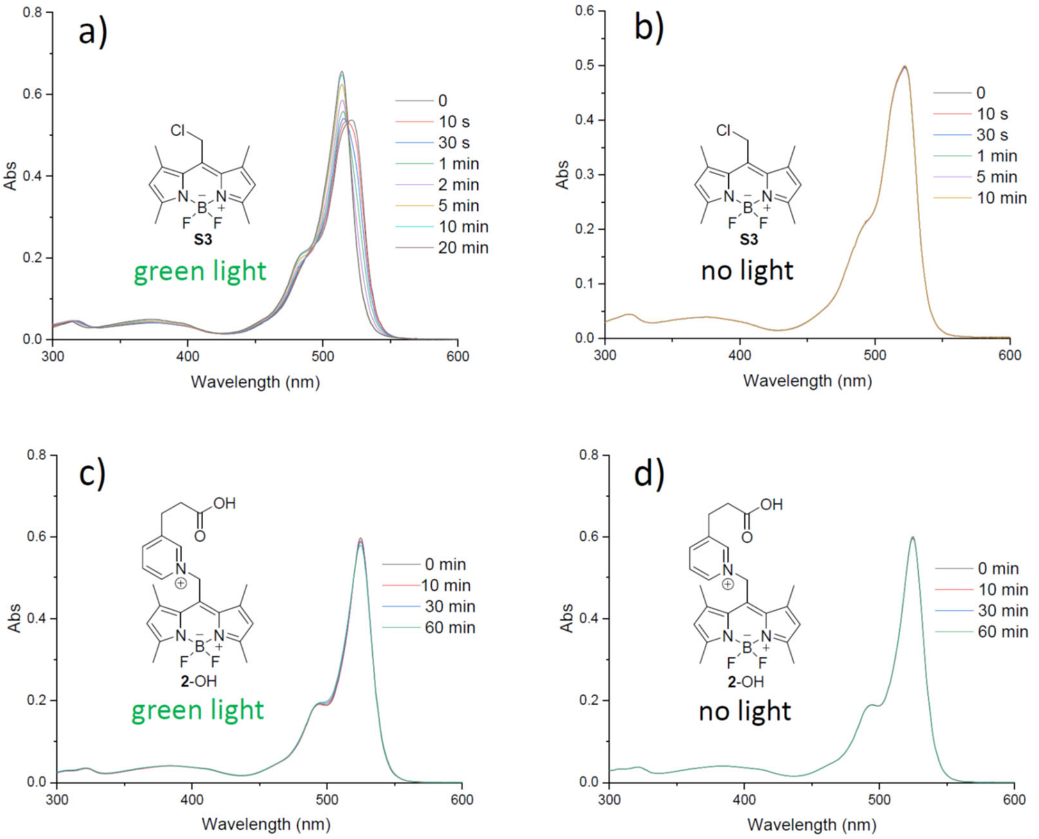

*Figure S18. UV-VIS spectra at various timepoints after irradiation under green light. a) **S2** is a reported photocage with a QY of ~1.6% in aerated MeOH. b) **S2** is stable in MeOH in the absence of light. c) The UV-VIS spectrum of **2**-OH shows hardly any changes even after prolonged irradiation, despite a very similar absorption maximum to **S2**. d) Spectra of **2**-OH taken at various timepoints without irradiation and comparison with spectra recorded after irradiation indicate that **2**-OH has some, but very little photoactivity.*

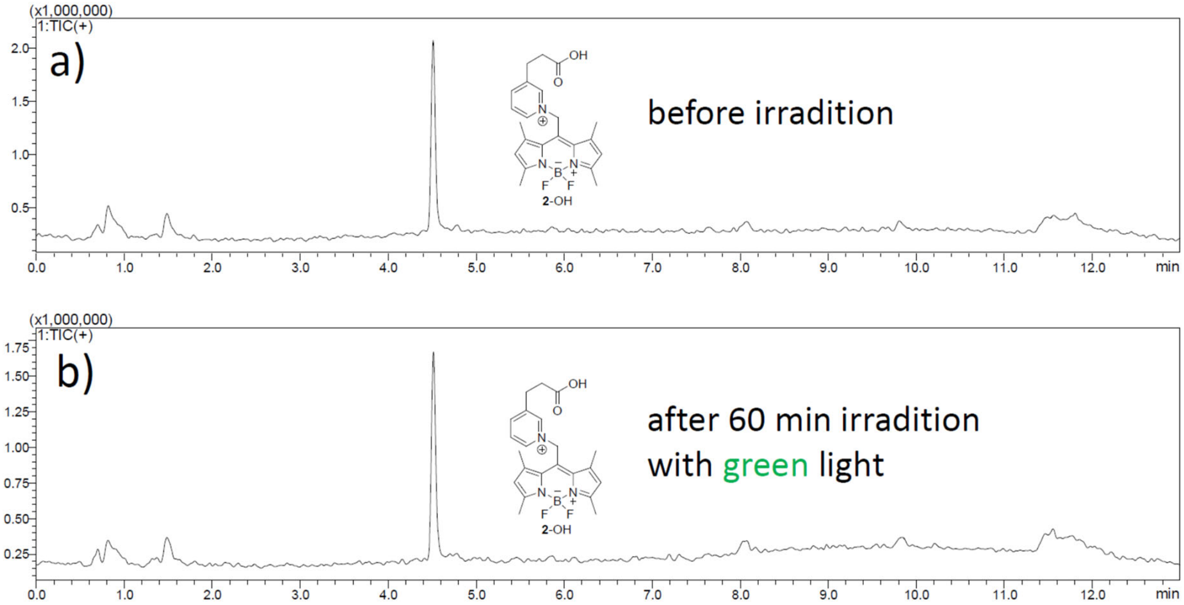

*Figure S19. Irradiation of **2**-OH with green light in MeOH does not lead to a significant change in the appearance of the LC-MS chromatogram (total ion count; scan range m/z = 100 - 950). Only the signal of intact **2**-OH was detected. a) LC-MS trace of **2**-OH before irradiation with green light. b) LC-MS trace of **2**-OH after irradiation with green light for 60 min.*

5. Stability of 2-OH and 3-OH in DMF

Compound **2**-OH showed very low stability in solvents such as DMF, NMP and DMSO, which excluded standard peptide coupling procedures (Figure S20a). The introduction of phenyl groups at boron increased the stability of the analogous Bodipy **3**-OH sufficiently to couple it to oxytocin in DMF.

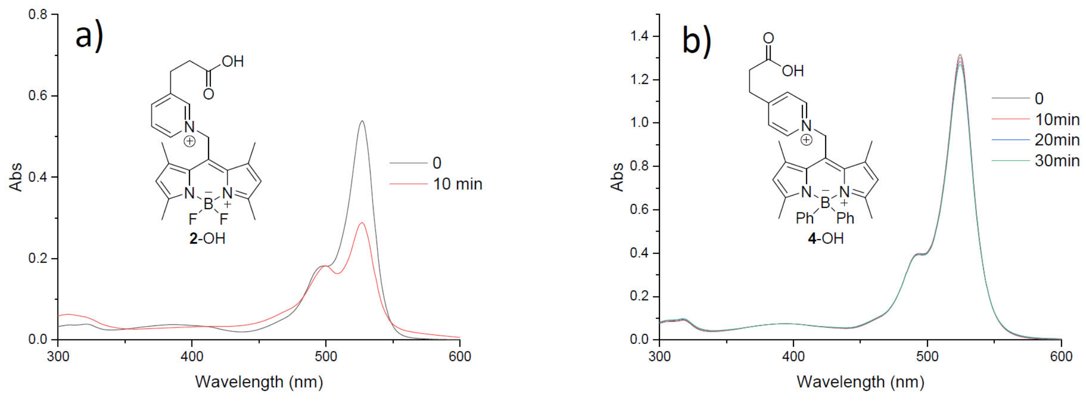

*Figure S20. Stability of **2**-OH and **3**-OH in DMF. a) UV-VIS spectra (Instrument: Cary 50) of **2**-OH in DMF directly after dissolution and of the same solution 10 minutes later. **2**-OH decomposes rapidly in DMF. b) **3**-OH shows in comparison a significantly improved stability in DMF. In between measurements the samples were kept in the dark.*

6. Excited state lifetimes and fluorescence quantum yields

The unexpected stability of **2**-OH in MeOH irradiated with green light (see Figures S18 and S19) prompted us to investigate its origin because it might affect the cleavage outcome in the gas phase. Therefore, we measured excited state lifetimes and fluorescence quantum yields of **2**-OH and a reference compound (see below) that contains the structurally identical bodipy chromophore for comparison.

Emission spectra (Figure S19) were measured on a HORIBA Fluorolog-3 spectrometer under excitation at 447 nm with a continuous wave laser (oxygen was removed from the sample solutions by bubbling the solutions with argon for a few minutes). Excitation spectra (Figure S23) were recorded on a HORIBA Fluoromax-4 with a xenon lamp. Fluorescence quantum yields (Table S1) were recorded on an Absolute PL Quantum Yield Spectrometer: Hamamatsu Quantaurus QY - C11347 (oxygen was removed from the sample solutions by bubbling the solutions with nitrogen for a few minutes).

The excited state lifetimes (Figure S22) were determined with LifeSpec II spectrometer (time-correlated single photon counting technique) from Edinburgh Instruments using picosecond pulsed diode lasers for excitation at 472 nm (oxygen was removed from the solutions by bubbling the solutions with argon for a few minutes). Quartz glass cuvettes with 10 mm path length were used. The concentrations indicated do not consider residual TFA in the solid samples after lyophilization. The

excess TFA content was estimated by comparing the integrals of the BF_2-group with CF_3-signal of TFA in ^{19}F NMR for one batch (16 weight%).

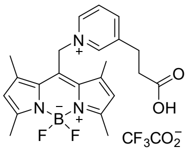

2-OH

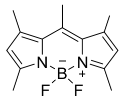

reference

The fluorescence in **2**-OH is partially quenched with respect to the reference bodipy compound and the effect is more pronounced in more polar MeOH. The measured fluorescence intensity decay curves required two exponentials to fit for **2**-OH unlike in the reference bodipy, in which the singlet excited state decays monoexponentially. We ruled out the possibility that the shorter component in the excited state decay is due to an impurity in our samples by measuring the excitation spectra (Figure S23). Therefore, the presence of two time constants to describe the kinetics of the excited state decay in **2**-OH clearly suggests that a new excited state process is present. This process shortens the prompt fluorescence but must be (pseudo)-reversible to account for the delayed component in the fluorescence intensity decay. The pyridinium moiety serves frequently as an electron acceptor, which suggests that this new process is most likely a photoinduced electron transfer from the bodipy chromophore to the pyridinium moiety. Clearly, such process is absent in the reference compound. The state energy diagram shown in Scheme S1 describes the involved excited states in **2**-OH and displays the involved processes that conform to the observed kinetics.

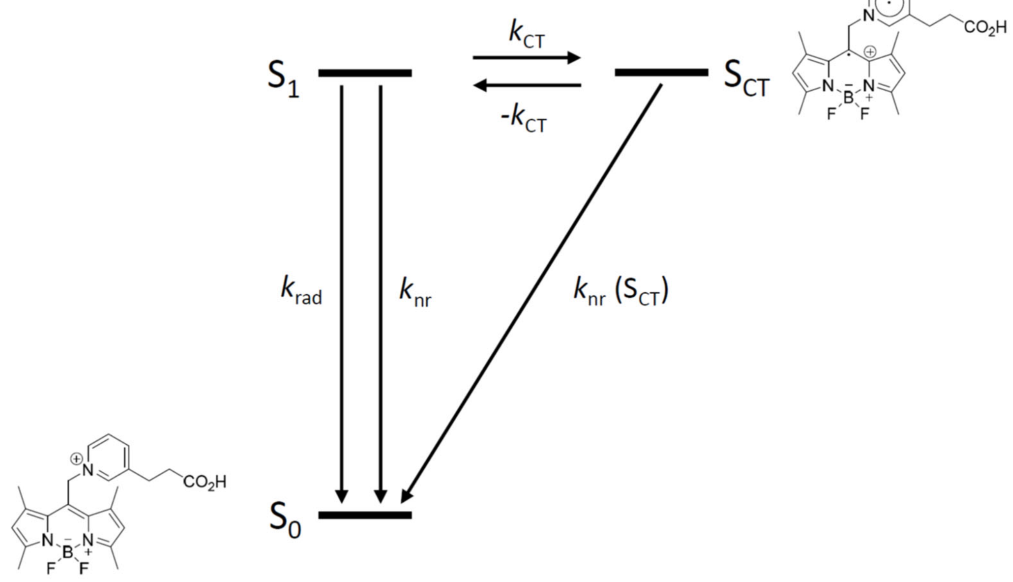

Scheme S1. Energy diagram to rationalize the two apparent time constants for excited state decay in time-correlated single photon counting measurements. Adapted from.[6]

After the excitation of **2**-OH, part of the excited state population undergoes electron transfer to form the singlet charge-transfer (S_{CT}) state. The rest decays via fluorescence or non-radiatively with a rate constant that will be similar to that measured for the reference compound. Therefore, the shortening of the singlet excited state lifetime to ~1.5 ns is largely due to the electron transfer. The process leading to the S_{CT} state seems more efficient in polar MeOH compared to CH_2Cl_2, a medium-polarity solvent, although the electron transfer is only accompanied by a charge shift and not by charge separation. Consequently, the weight of the faster component of the excited state decay increases from 0.44 in CH_2Cl_2 to 0.82 in MeOH and the prompt lifetime shortens further from 1.86 ns to 1.50 ns, respectively. Possibly, the S_{CT} state is slightly more stable in MeOH, which would also impact the reversibility of the electron transfer. The lower fluorescence quantum yield in MeOH clearly shows that the rate constant for the reverse electron transfer from the S_{CT} back to the singlet excited state of **2**-OH is lower in MeOH than in CH_2Cl_2 or that the electron transfer back to the ground state is faster. In any case, the major decay pathway of the S_{CT} in **2**-OH is the electron transfer back to the ground state S_0 of **2**-OH. This is clearly reflected in the weaker emission and the relatively high photostability of the compound when irradiated by green light. Note that intersystem crossing (ISC) from the S_{CT} state to the triplet T_1 state of **2**-OH seems unlikely in contrast to an analogous system described previously[5] where a pyridinium moiety was directly attached via C4 of the pyridine ring to the bodipy chromophore, i.e., without an additional methylene group. If a T_1 state was formed, it would compromise the stability of **2**-OH because bodipy and related photocages release their cargo more efficiently via a triplet state than the S_1 state.[1, 7] Compound **2**-OH would thus not be stable under irradiation in MeOH, which is contrary to our experimental observations. The reason for the inefficient ISC is the spatial orientation of the pyridinium moiety and the bodipy chromophore in **2**-OH. An efficient charge-transfer mediated ISC requires that the two moieties are perpendicular to each other, which is not fullfilled in **2**-OH unlike in the bodipy-pyridinium described previously.[8]

In conclusion, photoinduced electron transfer in **2**-OH is likely responsible for its stability under irradiation in polar MeOH. Whether such an electron transfer can take place in the gas phase is not known. Our TD-DFT calculations of the excited state potential energy surfaces and the corresponding MOs involved in the lowest energy transitions (see Figures S27, S32, S33) suggest that the S_{CT} state is not the lowest excited state in **2**-OH even upon stretching the C–N bond that leads to heterolysis.

Fluorescence spectra of **2**-OH and the reference compound

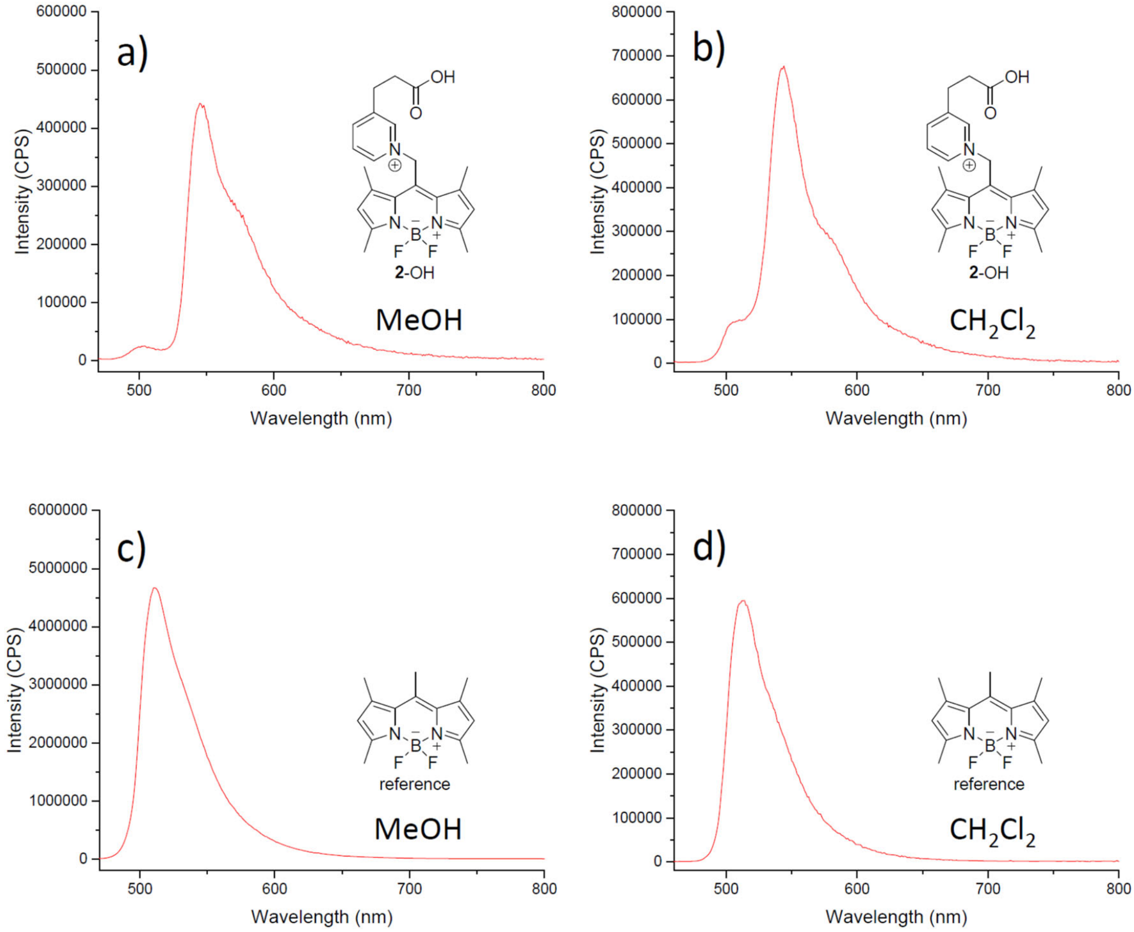

Figure S21. Fluorescence spectra (Instrument: Horiba Fluorolog 3, excitation wavelength = 447 nm) of ***2****-OH and the reference compound in MeOH and* CH_2Cl_2*. The counter anion for* ***2****-OH is* $CF_3CO_2^-$*. a) Emission spectrum of* ***2****-OH in MeOH. b) Emission spectrum of* ***2****-OH in* CH_2Cl_2*. c) Emission spectrum of of the reference compound in MeOH d) Emission spectrum of the reference compound in* CH_2Cl_2*.*

Fluorescence lifetime measurements of **2**-OH and the reference in compound

- **2**-OH in CH_2Cl_2 (ca. 6 µM, 550 nm detection wavelength, 1 nm slit width): 1.86 ns (44%), 5.44 ns (56%)
- **2**-OH in MeOH (ca. 6 µM , 550 nm detection wavelength, 1 nm slit width): 1.50 ns (82%), 5.58 ns (18%)
- reference in CH_2Cl_2 (7.2 µM, 515 nm detection wavelength, 1 nm slit width): 5.90 ns
- reference in MeOH (14.6 µM, 515 nm detection wavelength, 1 nm slit width): 6.38 ns

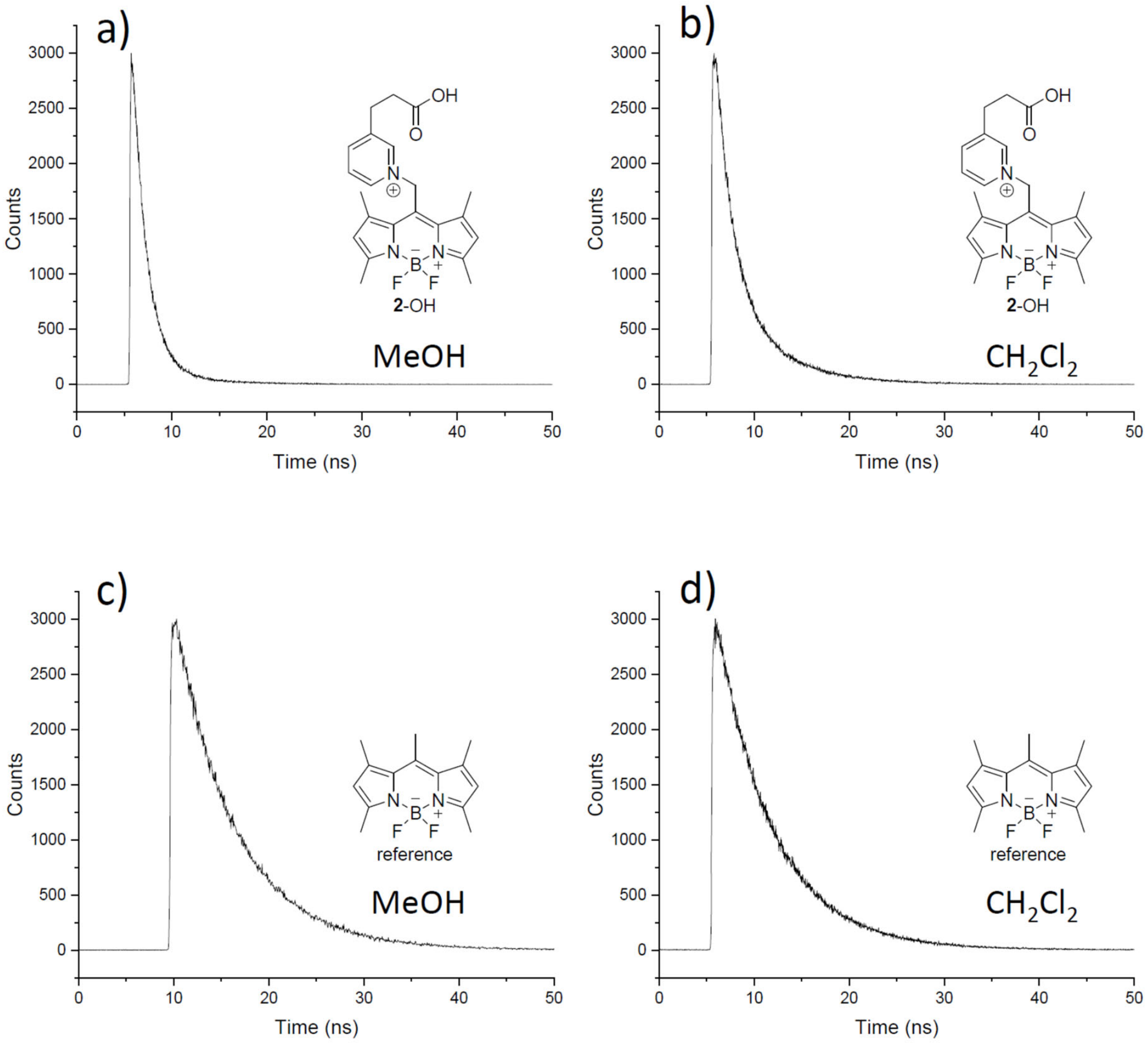

*Figure S22. Excited state lifetime measurements (Instrument Life-Spec II, excitation wavelength 472 nm) of **2**-OH and the reference compound in MeOH and CH_2Cl_2. The counterion for **2**-OH is $CF_3CO_2^-$. Fluorescence intensity decay curves: a) **2**-OH in MeOH; b) **2**-OH in CH_2Cl_2; c) the reference molecule in MeOH; d) the reference molecule in CH_2Cl_2.*

Fluorescence quantum yield determination of **2**-OH and the reference compound

	excitation wavelength(nm)	ϕ_f (MeOH)	ϕ_f (degassed MeOH)	ϕ_f (CH_2Cl_2)	ϕ_f (degassed CH_2Cl_2)
**2**-OH	**520**	**13.5%**	**15.8%**	**21.3%**	**25.7%**
reference	**480**	**89.0%**	**91.8%**	**77.3%**	**77.6%**

*Table S1. Fluorescence quantum yields (Instrument: Hamamatsu Quantaurus QY - C11347) of **2**-OH and the reference compound in MeOH and CH_2Cl_2. 'Degassed' solvents refer to solutions where oxygen was removed by bubbling N_2 through the solutions for a few minutes.*

Excitation spectra of **2**-OH

Although the total ion count (TIC) trace in LC-MS and the ^{1}H-NMR of **2**-OH indicated high purity of the sample, we recorded excitation spectra to exclude that the second observed excited state lifetime is due to an impurity. The data support our assumption of two competing processes within the same molecule. The excitation spectra recorded at different excitation wavelength are nearly identical to each other and to the absorption spectrum of **2**-OH.

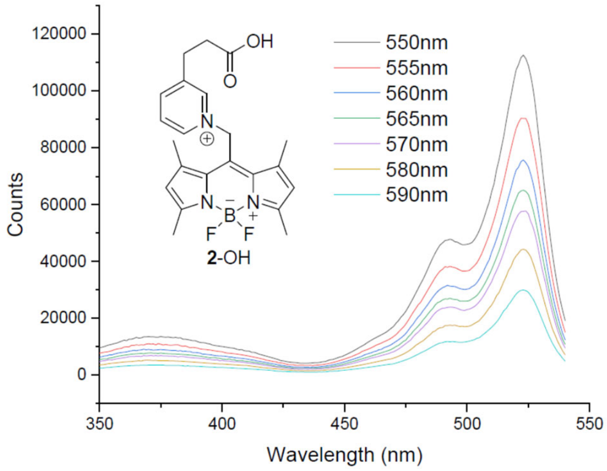

*Figure S23. Excitation spectra (Instrument: HORIBA Fluromax-4) of **2**-OH in MeOH at various excitation wavelengths. The counteranion is $CF_3CO_2^-$.*

7. UV-Vis Spectra

UV-Vis absorption of **1**-pNP and **2**-OH in solvents of different relative permittivity

To evaluate the solvatochromic properties of the photocages and thereby assess potential shifts of their absorption spectra in high vacuum, we recorded solution phase spectra in solvents with different relative permittivity. Since **1**-GGF is poorly soluble in solvents of low polarity we used **S2** as a proxy with an identical bodipy chromophore. A comparison of the spectra shows that both compounds display only subtle solvatochromism. For **S2** the absorption maximum is red-shifted by 3 nm when changing from the solvent of the highest polarity (DMSO-d_6) to the one of the lowest polarity (cyclohexane); for **2**-OH the position of the absorption maximum is at 525 nm in both, MeOH and 1,4-dioxane.

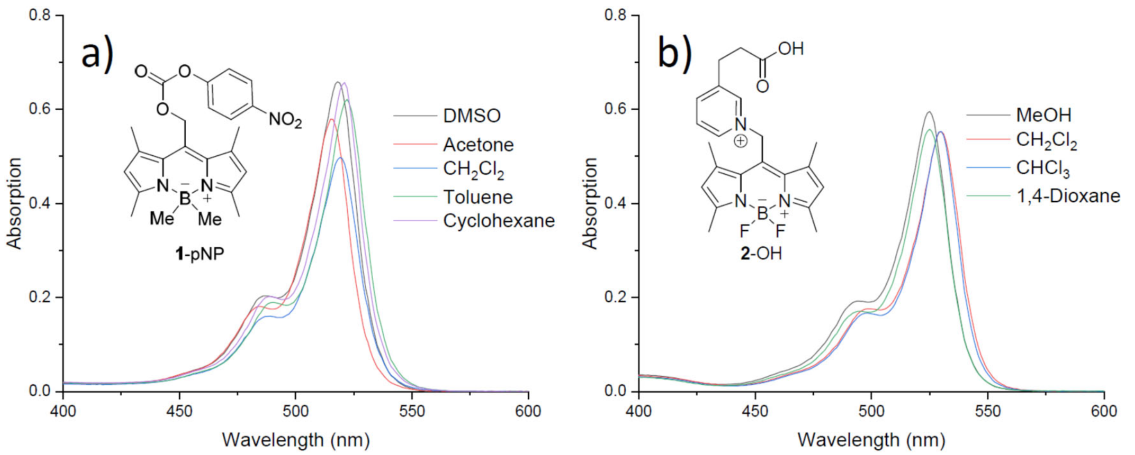

*Figure S24. Absorption spectra (Instrument: Cary 50) of **1**-pNP and **2**-OH in various solvents. The counteranion for **2**-OH is $CF_3CO_2^-$ a) Absorption spectra of **S2**. Note the small shift of only 3 nm in the solvent with the lowest (cyclohexane) and highest (DMSO-d_6) relative dielectric constant ε_r. b) Absorption spectra of **2**-OH. Note the identical position of the absorption maximum in MeOH and 1,4-dioxane.*

	DMSO	Acetone	CH_2Cl_2	Toluene	Cyclohexane
ε_r [a)]	**47.24**	**21.01**	**8.93**	**2.38**	**2.02**
λ_{max} (nm)	**518**	**515**	**519**	**522**	**521**

Table S2. a) dielectric constants taken from: CRC Handbook of Chemistry and Physics, 89th Ed., Editor D. R. Lide, CRC Press, Boca Raton, Florida, USA, 2008

	MeOH	CH_2Cl_2	$CHCl_3$	1,4-Dioxane
ε_r [a)]	**33.0**	**8.93**	**4.81**	**2.22**
λ_{max} (nm)	**525**	**530**	**530**	**525**

Table S3. a) dielectric constants taken from: CRC Handbook of Chemistry and Physics, 89th Ed., Editor D. R. Lide, CRC Press, Boca Raton, Florida, USA, 2008

UV-Vis absorption spectra of the compounds in MeOH

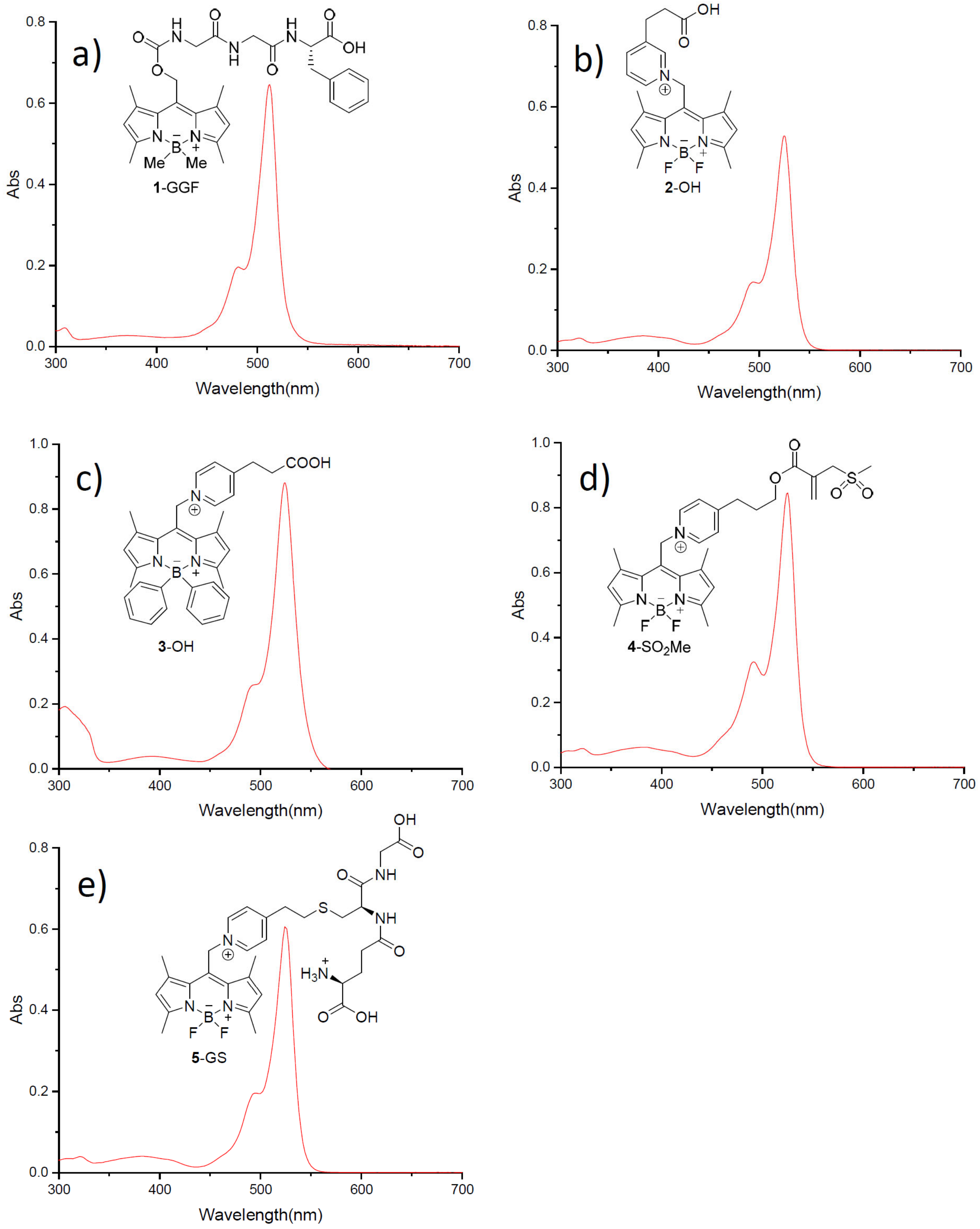

*Figure S25. Absorption spectra (Instrument: Cary 50) in MeOH of compounds a) **1**-GGF, b) **2**-OH, c) **3**-OH, d) **4**-SO_2Me,e) **5**-GS.Counteranions are $CF_3CO_2^-$.*

8. Gas phase photocleavage experiments

General set-up

Experiments were performed using a customized Waters Q-TOF Ultima (©) mass spectrometer, as illustrated in Fig. S26. The electro-sprayed ions are collected by two ion funnels and guided through the vacuum system until they enter a quadrupole mass filter (QMS), where they are mass selected. Two transfer hexapoles and a DC-guide are used to guide the ions to the TOF. A 45° mirror is placed in the high vacuum environment to align the laser (alternatively Edge Wave INNOSLAB or InnoLas SpitLight EVO I) collinearly and counter-propagating to the molecular ion beam.

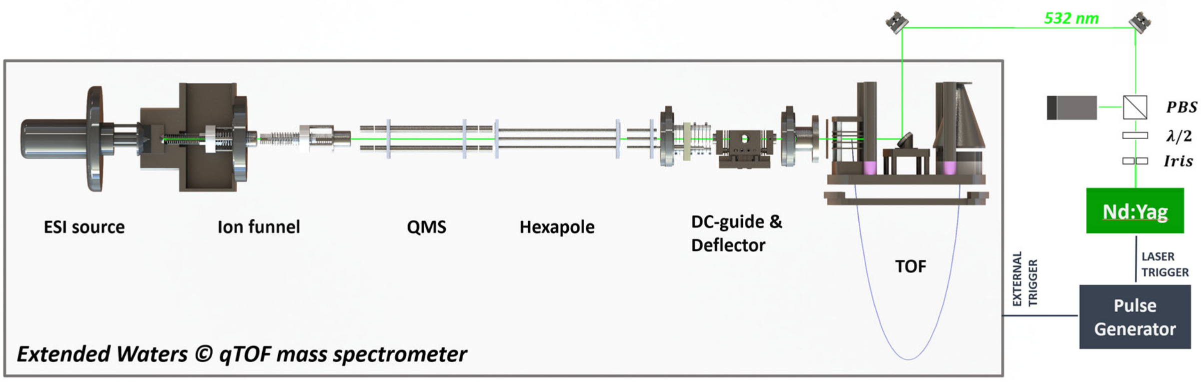

Figure S26. Experimental setup. Ions are electro-sprayed and mass selected by a quadrupole mass filter (QMS) before interacting with a light pulse (10 ns, 532 nm), which is collinearly aligned with the laser beam.

Molecular beam preparation

All peptides were sprayed using standard spray conditions. 10 - 20 µM solutions of the peptides were prepared in either water or a mixture of water and acetonitrile under addition of a small amount of formic acid. The solution was filled into a syringe pump and sprayed through a 125 µm capillary, with a flow rate of 4 µL/min.

Mass filtering

The mass spectrometer was upgraded by MSVISION (©) for a maximal mass range of m/q =30'000 Da/e. This system still achieves atomic mass resolution in the region of interest, which we exploit to distinguish between heterolytic, homolytic processes and those accompanied by H-transfer. The mass filter operates at a base pressure of 10^{-6} mbar. The TOF-MS operates at 10^{-7} mbar. At pressures above 10^{-6} mbar collision-induced cleavage was observed as an additional contribution to the signal.

Photochemistry

The green laser pulse is generated by frequency doubling a diode pumped Nd:YAG laser. The light was spatially filtered to obtain a near-Gaussian intensity profile with a beam waist of w_0 =1.6 mm ($1/e^2$-value). The laser power was regulated by combining a $\lambda/2$ waveplate with a polarizing beam splitter (PBS). For all experiments, the laser pulse energy was between 0.2 - 3 mJ, which is sufficient to allow efficient cleavage of all compounds. The laser beam entered through the top-lid of the TOF-MS and it was directed by a 45° mirror towards the entrance hole of the customized ESI interface. The smallest beam constriction was the ion lens close to the TOF-MS with a height of 1.7 mm.

Photofragmentation mass spectra

Mass spectra were obtained by dividing the TOF-MS pusher frequency into a pulse train of 100 Hz, which triggered the laser emission and the Waters © QTOF 4 GHz time to digital converter (TDC). This allowed us to adapt the delay between the laser and the TOF frequency to maximize the signal for depletion or fragmentation. It also ensured that every ion saw exactly one laser pulse. In these experiments the delay between the laser pulse and the TOF extraction frequency was set to maximize the detection of all fragments of the cleavage process. To verify that all observed fragments are products of photochemistry, we compared mass spectra with and without laser light. All mass spectra were stored as CSV files and analyzed using the ORIGIN Pro 2021 software.

9. Calculations

General Remarks

All calculations were performed with Gaussian16 rev. C.01 suite of electronic structure programs.[9] The geometries of the potential energy minima or transition states were optimized at B3LYP/6-31G(d) level of theory. To reduce computational cost, the structures of **1**-GGF and **2**-GGF were simplified by truncating the GGF-peptide residue to a methyl substituent (see Figure S27 below). Compound **2**-OH was simplified by replacing the propanoic acid residue with a hydrogen atom.

The nature of all stationary points was verified by frequency calculations. The nature of the transition states was verified by performing intrinsic reaction coordinate (IRC) calculations that connected the transition state structure with the potential energy minima. Due to computed low imaginary force constants, the IRC paths did not always fully converge to the corresponding energy minima. Consequently, the geometry of the last point of the IRC path was optimized by calculating the full Hessian matrix in each optimization step to make sure that the optimization converged to the correct energy minimum. The wavefunction stability of the methylene-bodipy cations (see below) was tested. If an instability was found, the broken spin-symmetry (BS) Kohn-Sham wavefunctions were computed, in which the spatial symmetries of the α and β MOs are destroyed. Such wavefunction was then used to optimize the geometry of the molecule. We denote such calculations here as BS-DFT. The single point energies were then calculated with various DFT functionals and the cc-pVTZ basis set. The reported energies (at 0K) given in kcal mol^{-1} represent the sum of the total electronic energy and the unscaled zero-point energy correction. The potential energy surface scans for the S_0 ground state were performed on the B3LYP/6-31G(d) level of theory and for the S_1 excited state were performed on the TD-CAM-B3LYP/6-31G(d) level of theory to avoid spurious intrusion of the charge-transfer states in **2**-OH as observed with B3LYP functional. Both scans, for S_0 and S_1 states, were carried out along the C–O bond stretching coordinate in **1**-GGF, **2**-GGF and of the C–N bond stretching coordinate in **2**-OH, while all other coordinates were relaxed in the optimization.

In the ground state, stretching of the bonds in the relaxed ground state potential energy surface scans also lead to instability of the wavefunctions due to the diradicaloid nature of the ensuing methylene-bodipy. We tested the wavefunction stability in these cases and calculated the energy using the broken spin-symmetry Kohn-Sham wavefunctions to estimate the effect on the shape of the potential energy surface.

Results

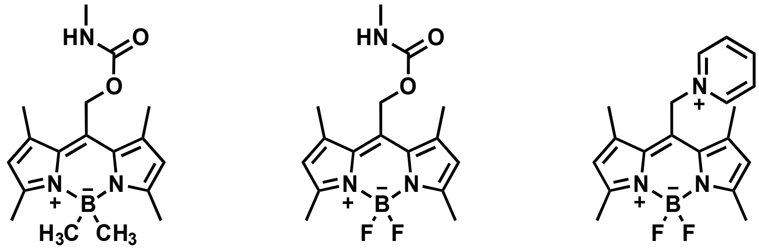

truncated **1**-GGF **2**-GGF **2**-OH

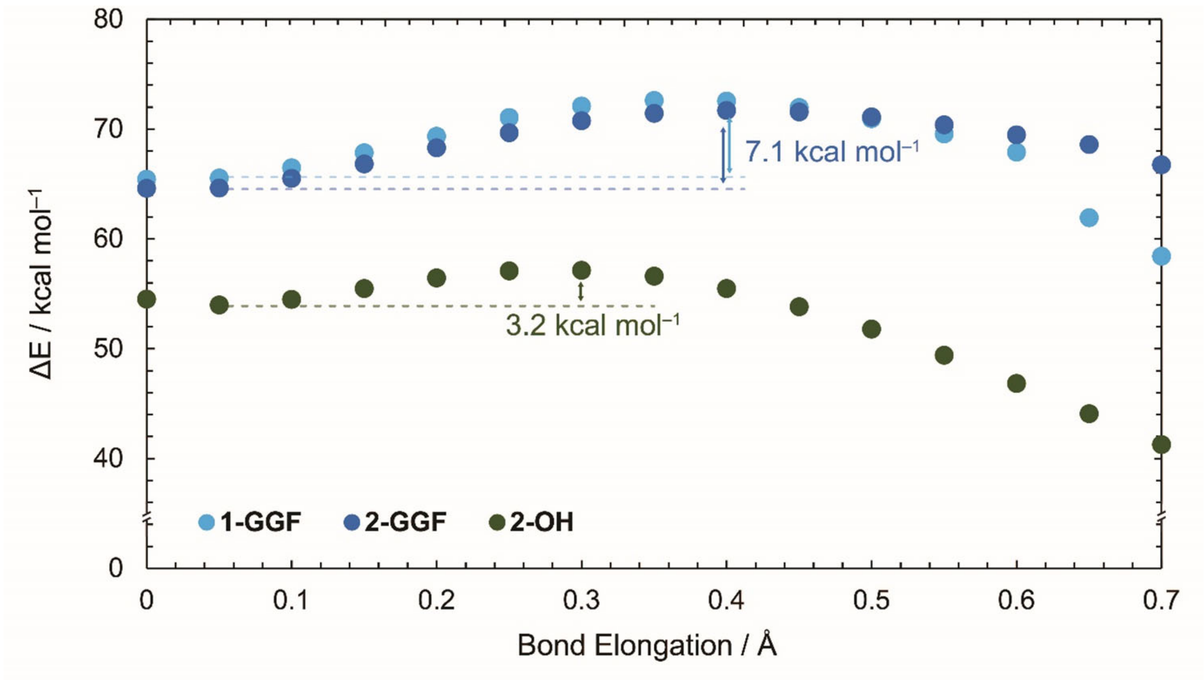

*Figure S27. The S_1 excited state potential energy surface scans along the C–O bond stretching coordinate in **1**-GGF (blue), **2**-GGF (dark blue) and of the C–N bond in **2**-OH (dark green) from the S_1 state energy minimum. TD-CAM-B3LYP/6-31G(d) level of theory. The optimized bond lengths in the S_1 state are $d_{C–O}$ = 1.44 Å (**1**-GGF), $d_{C–O}$ = 1.54 Å (**2**-GGF), and $d_{C–N}$ = 1.52 Å (**2**-OH).*

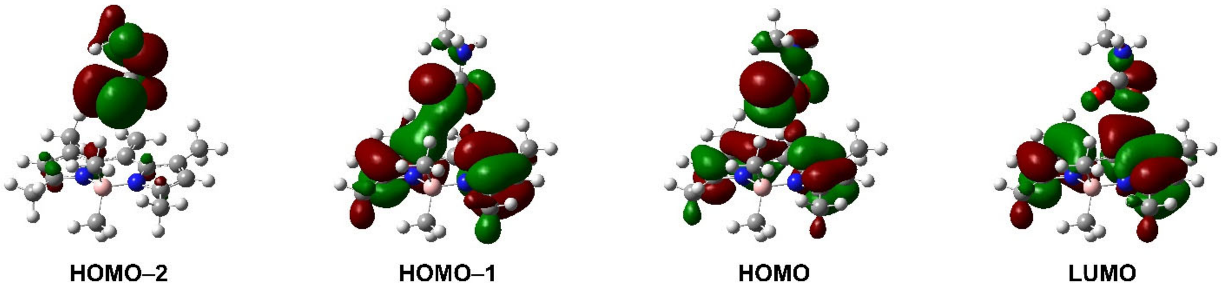

Transition	Calc. λ_{max} / nm	*E* / eV	*f*
HOMO→LUMO	416.51	2.9767	0.4918
HOMO–1→LUMO	355.60	3.4866	0.0380
HOMO–2→LUMO	313.16	3.9592	0.0248

FigureS28. The HOMO–2, HOMO–1, HOMO, and LUMO of ***1****-GGF in the energy minimum of the S_1 state (level of theory: TD-CAM-B3LYP/6-31G(d)).*

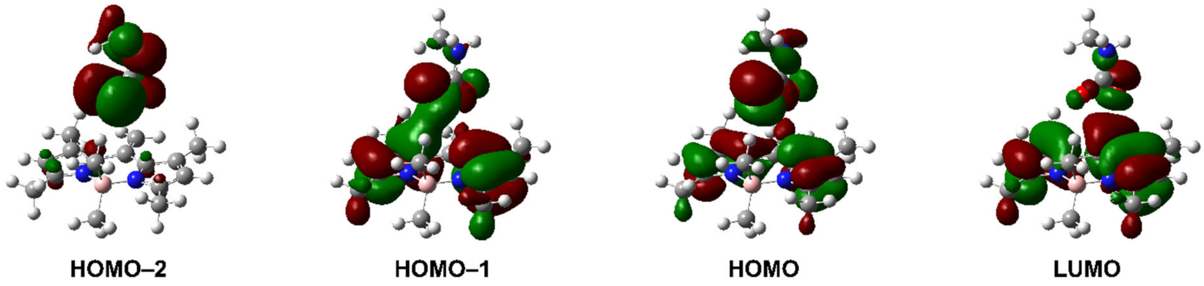

Transition	Calc. λ_{max} / nm	*E* / eV	*f*
HOMO→LUMO	2949.26	0.4204	0.0006
HOMO–1→LUMO	787.38	1.5746	0.1673
HOMO–2→LUMO	554.78	2.2348	0.0001

Figure S29. The HOMO–2, HOMO–1, HOMO, and LUMO of ***1****-GGF with C–O bond stretched by 0.8 Å in the S_1 state (level of theory: TD-CAM-B3LYP/6-31G(d)).*

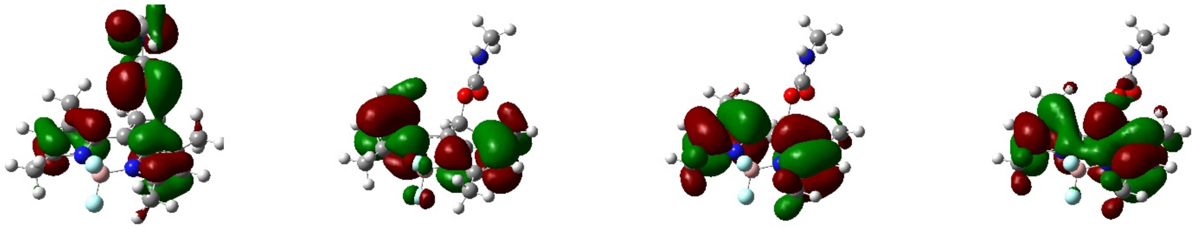

HOMO–2 HOMO–1 HOMO LUMO

Transition	Calc. λ_{max} / nm	E / eV	f
HOMO→LUMO	423.94	2.9246	0.5266
HOMO–1→LUMO	328.99	3.7687	0.0523
HOMO–2→LUMO	307.60	4.0308	0.0365

*Figure S30. The HOMO–2, HOMO–1, HOMO, and LUMO of **2**-GGF in the energy minimum of the S_1 state (level of theory: TD-CAM-B3LYP/6-31G(d)).*

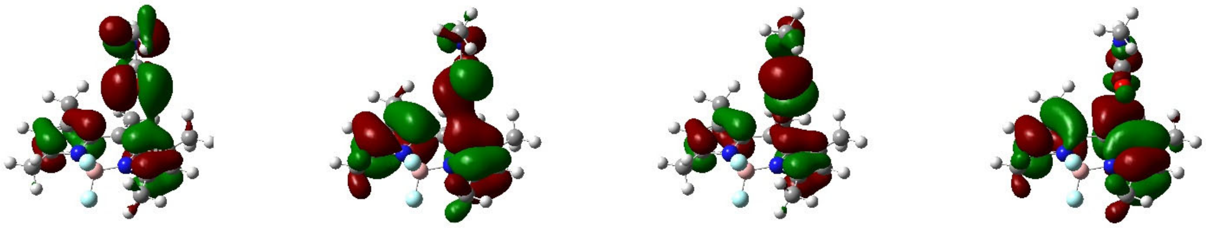

HOMO–2 HOMO–1 HOMO LUMO

Transition	Calc. λ_{max} / nm	E / eV	f
HOMO→LUMO	3872.63	0.3202	0.0003
HOMO–1→LUMO	770.45	1.6092	0.1893
HOMO–2→LUMO	576.15	2.1520	0.0003

*Figure S31. The HOMO–2, HOMO–1, HOMO, and LUMO of **2**-GGF with C–O bond stretched by 0.8 Å in the S_1 state (level of theory: TD-CAM-B3LYP/6-31G(d)).*

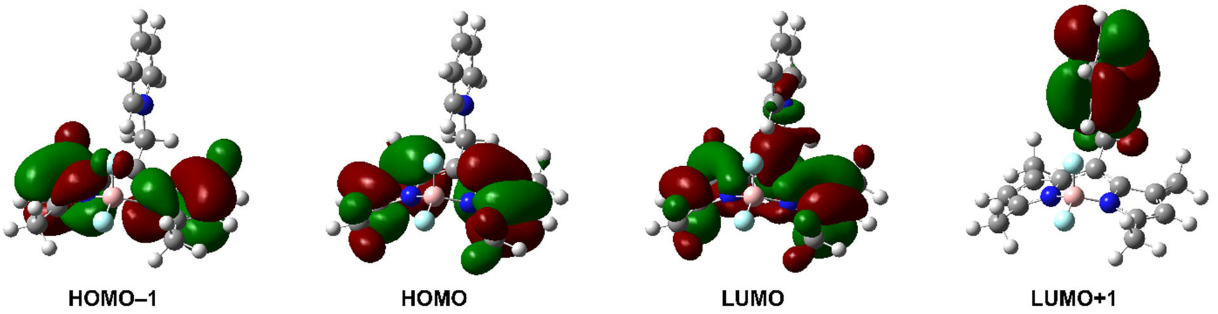

Transition	Calc. λ_{max} [nm]	*E* [eV]	f_{os}
HOMO→LUMO	526.66	2.3542	0.3671
HOMO→LUMO+1	432.75	2.8650	0.0004
HOMO–1→LUMO	396.07	3.1303	0.0341

Figure S32. The HOMO–1, HOMO, LUMO, and LUMO+1 of ***2-OH*** *in the energy minimum of the* S_1 *state (level of theory: TD-CAM-B3LYP/6-31G(d)).*

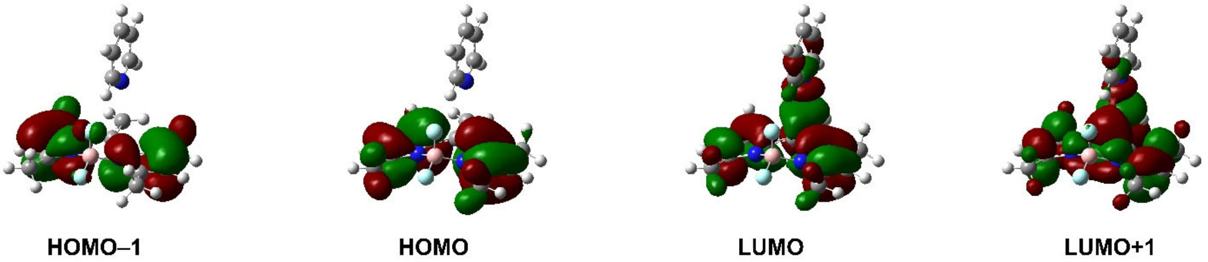

Transition	Calc. λ_{max} / nm	*E* / eV	*f*
HOMO→LUMO	1072.83	1.1557	0.0905
HOMO–1→LUMO	558.53	2.2198	0.0000
HOMO→LUMO+1	340.03	3.6462	0.0003

Figure S33. The HOMO–1, HOMO, LUMO, and LUMO+1 of ***2-OH*** *with C–N bond stretched by 0.8 Å in the* S_1 *state (level of theory: TD-CAM-B3LYP/6-31G(d)).*

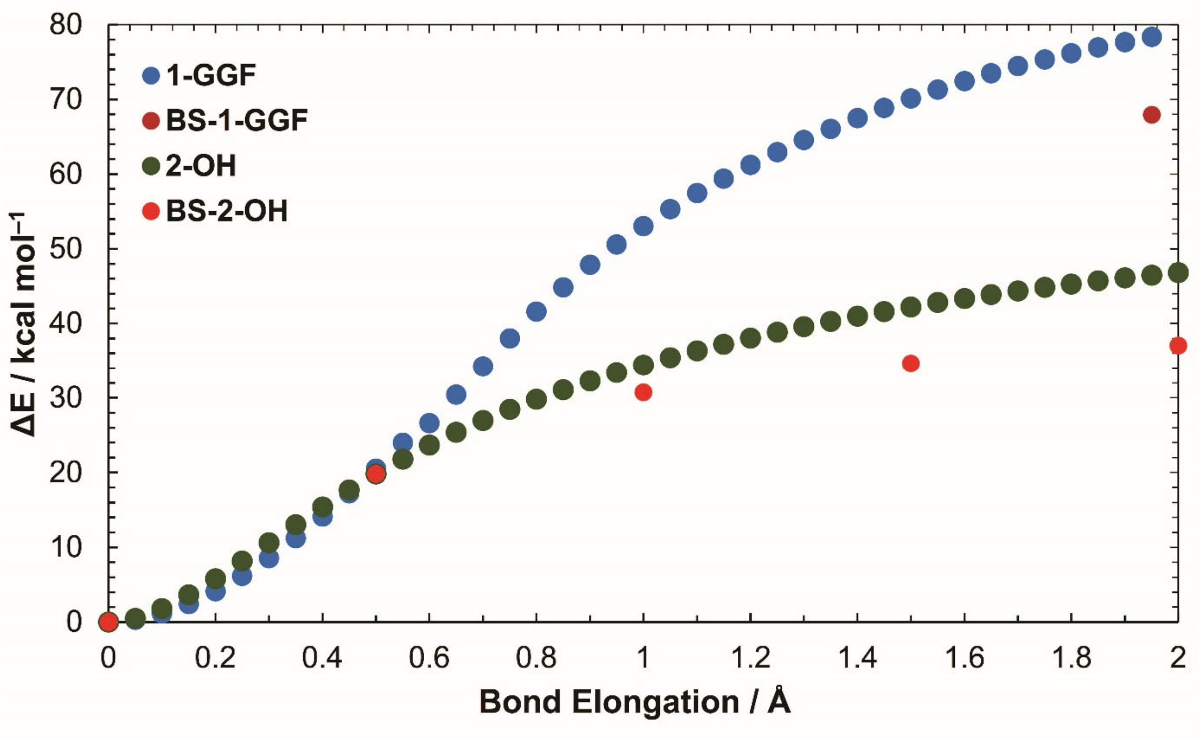

*Figure S34. The S_0 ground state potential energy surface scans along the C–O bond stretching coordinate in **1**-GGF (blue), and of the C–N bond stretching coordinate in **2**-OH (dark green) from the ground state energy minimum. B3LYP/6-31G(d) level of theory. The broken-symmetry corrected energies are calculated for **1**-GGF (dark red) and **2**-OH (red) at the BS-B3LYP/6-31G(d) level of theory. The optimized bond lengths are $d_{C\text{–}O}$ = 1.44 Å (**1**-GGF) and $d_{C\text{–}O}$ = 1.54 Å (**2**-OH).*

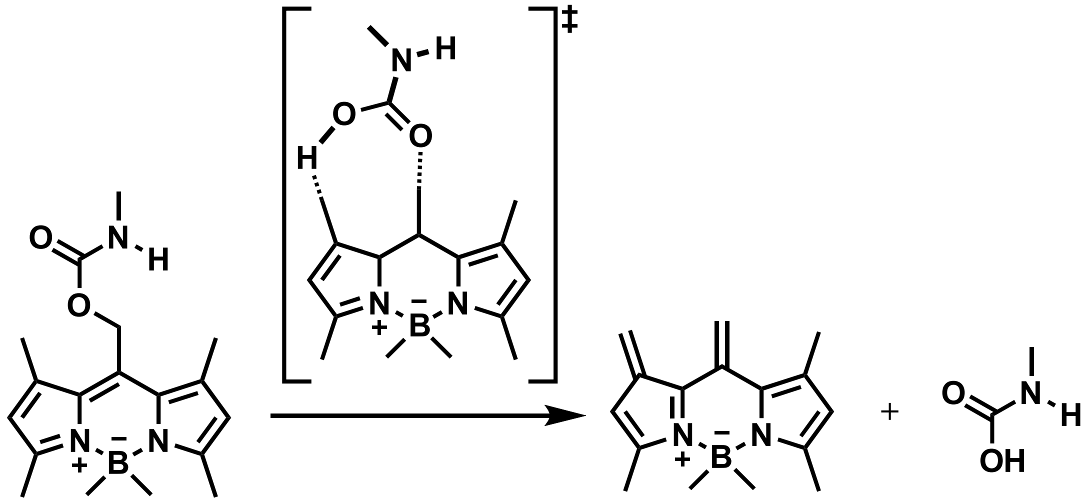

Method	Δ*E*/ kcal mol^{-1}	Δ*E* / eV	ΔE† / kcal mol^{-1}	ΔE† / eV
B3LYP	26.6	1.15	52.5	2.26
BMK	32.5	1.40	58.6	2.52
M06-2X	32.8	1.41	55.9	2.40
wB97XD	32.9	1.41	55.5	2.39

Table S4. Calculated reaction (ΔE) and the transition state (E†) energies of the sigmatropic hydrogen transfer and C–O bond cleavage in ***1****-GGF in the ground state at different levels of theory. Calculations were performed using the cc-pVTZ basis-set with B3LYP/6-31G(d) optimized geometries. Energies at 0K.*

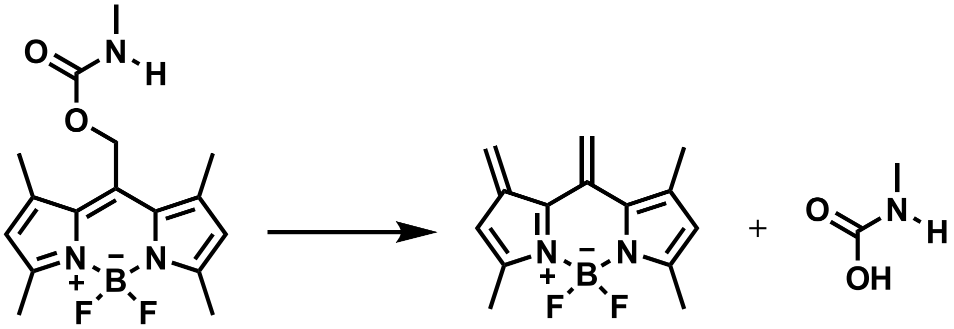

Method	Δ*E* / kcal mol⁻¹	Δ*E* / eV
B3LYP	29.0	1.25
BMK	34.6	1.49
M06-2X	34.9	1.50
wB97XD	35.0	1.50

Table S5. Calculated reaction energies (ΔE) of the sigmatropic hydrogen transfer and C–O bond cleavage in ***2-GGF*** *in the ground state at different levels of theory. Calculations were performed using the cc-pVTZ basis-set with B3LYP/6-31G(d) optimized geometries. Energies at 0K.*

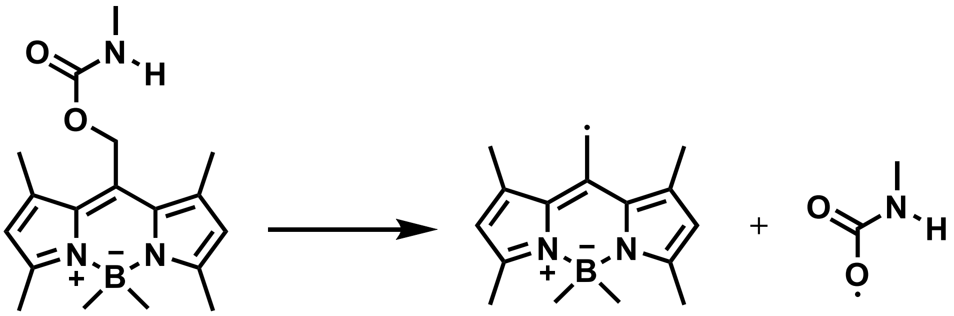

Method	Δ*E* / kcal mol⁻¹	Δ*E* / eV
B3LYP	59.9	2.58
BMK	72.4	3.11
M06-2X	78.6	3.38
wB97XD	71.1	3.06

Table S6. Calculated reaction energies (ΔE) of the homolytic C–O bond cleavage in ***1****-GGF in the ground state at different levels of theory. Calculations were performed using the cc-pVTZ basis-set with B3LYP/6-31G(d) optimized geometries. Energies at 0K.*

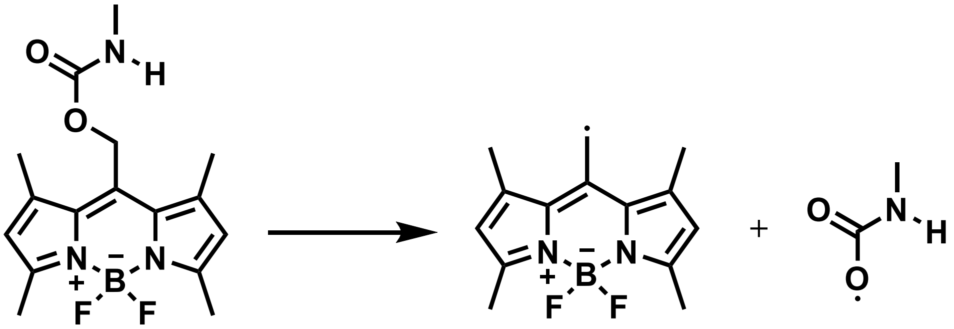

Method	Δ*E* / kcal mol⁻¹	Δ*E* / eV
B3LYP	60.2	2.59
BMK	72.5	3.12
M06-2X	78.9	3.39
wB97XD	71.5	3.07

Table S7. Calculated reaction energies (ΔE) of the homolytic C–O bond cleavage in ***2****-GGF in the ground state at different levels of theory. Calculations were performed using the cc-pVTZ basis-set with B3LYP/6-31G(d) optimized geometries. Energies at 0K.*

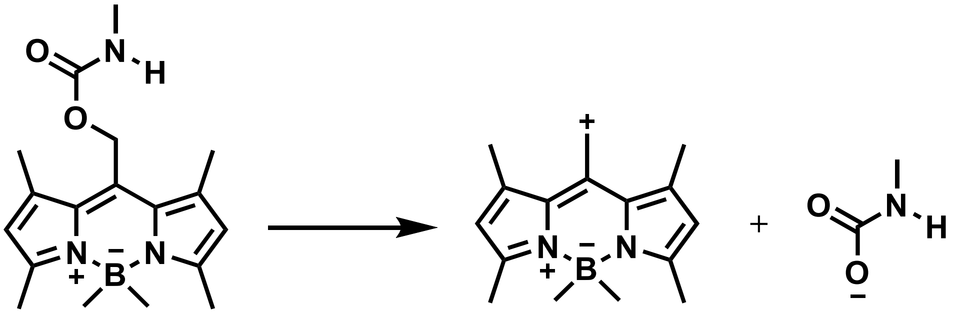

Method	Δ*E* / kcal mol⁻¹	Δ*E* / eV
B3LYP	206.2	8.86
BMK	218.2	9.38
M06-2X	225.7	9.70
wB97XD	223.9	9.63

Table S8. Calculated reaction energies (ΔE) of the heterolytic C–O bond cleavage in ***1****-GGF in the ground state at different levels of theory. Calculations were performed using the cc-pVTZ basis-set with B3LYP/6-31G(d) optimized geometries. Energies at 0K.*

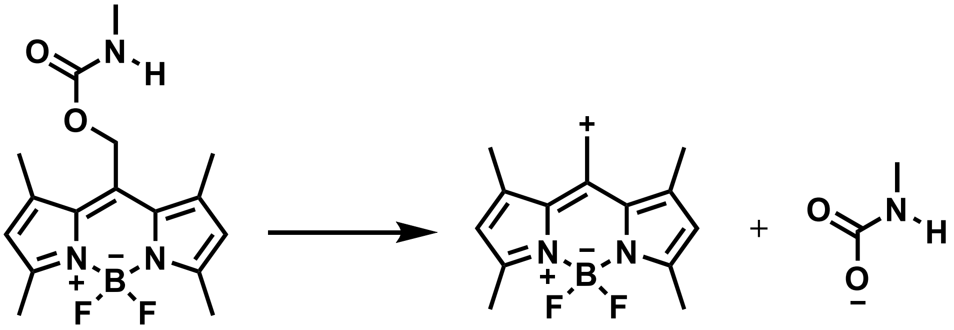

Method	ΔE / kcal mol⁻¹	ΔE / eV
B3LYP	155.2	6.68
BMK	161.9	6.96
M06-2X	166.3	7.15
wB97XD	160.2	6.89

*Table S9. Calculated reaction energies (ΔE) of the heterolytic C–O bond cleavage in **2**-GGF in the ground state at different levels of theory. Calculations were performed using the cc-pVTZ basis-set with B3LYP/6-31G(d) optimized geometries. Energies at 0K.*

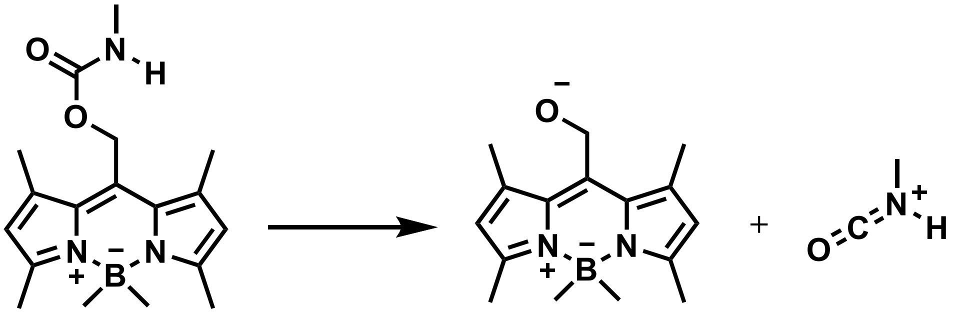

Method	ΔE / kcal mol⁻¹	ΔE / eV
B3LYP	189.7	8.16
BMK	196.8	8.46
M06-2X	199.4	8.58
wB97XD	196.5	8.45

*Table S10. Calculated reaction energies (ΔE) of the heterolytic C–O bond cleavage in **1**-GGF in the ground state at different levels of theory. Calculations were performed using the cc-pVTZ basis-set with B3LYP/6-31G(d) optimized geometries. Energies at 0K.*

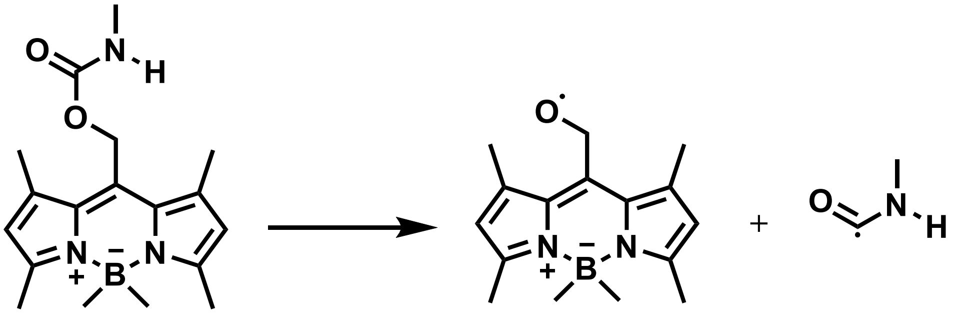

Method	ΔE / kcal mol^{-1}	ΔE / eV
B3LYP	86.1	3.70
BMK	94.1	4.05
M06-2X	98.8	4.25
wB97XD	94.4	4.06

*Table S11. Calculated reaction energies (ΔE) of the homolytic C–O bond cleavage in **1**-GGF in the ground state at different levels of theory. Calculations were performed using the cc-pVTZ basis-set with B3LYP/6-31G(d) optimized geometries. Energies at 0K.*

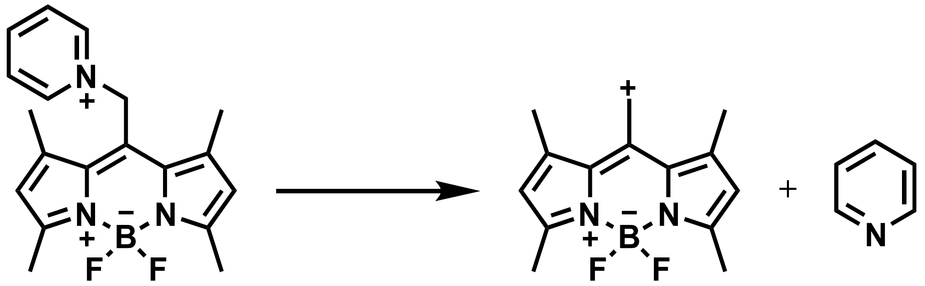

Method	ΔE / kcal mol^{-1}	ΔE / eV
B3LYP	34.4	1.48
BMK	42.2	1.81
M06-2X	44.9	1.93
wB97XD	41.8	1.80

*Table S12. Calculated reaction energies (ΔE) of the heterolytic C–N bond cleavage in **2**-OH in the ground state at different levels of theory. Calculations were performed using the cc-pVTZ basis-set with B3LYP/6-31G(d) optimized geometries. Energies at 0K.*

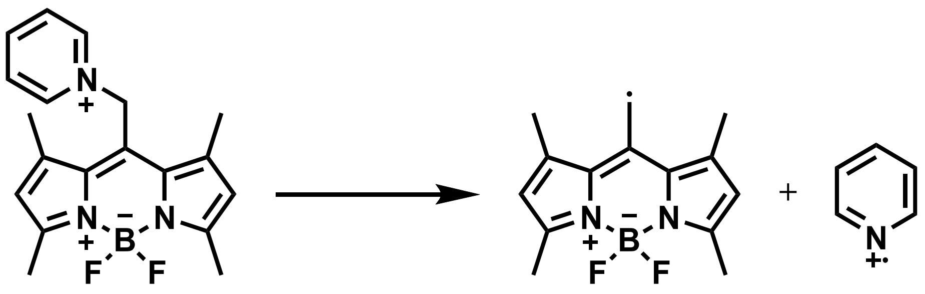

Method	ΔE / kcal mol⁻¹	ΔE / eV
B3LYP	82.3	3.54
BMK	90.2	3.88
M06-2X	95.7	4.12
wB97XD	94.6	4.07

*Table S13. Calculated reaction energies (ΔE) of the homolytic C–N bond cleavage in **2**-OH in the ground state at different levels of theory. Calculations were performed using the cc-pVTZ basis-set with B3LYP/6-31G(d) optimized geometries. Energies at 0K.*

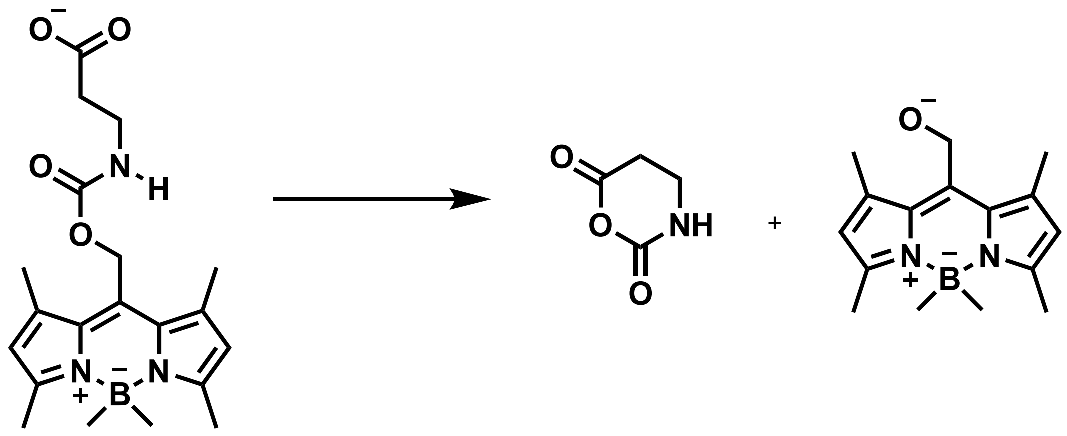

Method	ΔE/ kcal mol⁻¹	ΔE / eV
B3LYP	50.6	2.2
BMK	54.6	2.3
M06-2X	56.7	2.4
wB97XD	54.9	2.4

Table S14. Calculations were performed using the cc-pVTZ basis-set with B3LYP/6-31G(d) optimized geometries. Energies at 0K.

Cartesian Coordinates

2-GGF, Charge = 0, singlet, B3LYP/6-31G(d), *E* = –1161.206495 Hartree, *ZPVE* = 0.353581 Hartree / Particle.

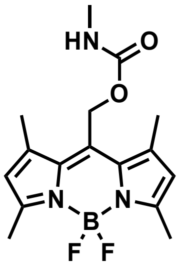

N	-4.55162400	0.00015200	1.19244600
C	0.40864200	-1.22347000	-0.30948600
N	1.77186700	-1.24420500	0.00562100
B	2.69825700	-0.00003700	0.14783700
N	1.77191200	1.24416500	0.00562700
C	0.40868700	1.22348400	-0.30948600
C	-0.25522200	0.00001800	-0.49641200
C	-0.04831400	-2.58567700	-0.35184200
C	1.06124900	-3.37116400	-0.06672700
C	2.16856500	-2.52368700	0.14296300
C	2.16866200	2.52363200	0.14296200
C	1.06138100	3.37115300	-0.06673200
C	-0.04821700	2.58571000	-0.35183300
C	-1.70408600	0.00003800	-0.92445000
C	-1.42569500	-3.13038600	-0.59861100
C	3.57617600	-2.90237800	0.46858500
C	-1.42557700	3.13047900	-0.59858700
C	3.57629000	2.90226700	0.46857500
O	-2.52455300	0.00010100	0.26307900
C	-3.87288900	0.00007900	0.01466300
O	-4.35968700	-0.00005800	-1.10154800
H	1.08237900	4.45174500	-0.00316200
H	1.08220300	-4.45175700	-0.00315500
C	-6.00025500	-0.00006300	1.25697500
H	-4.00579600	0.00012700	2.04172000
H	-1.94039300	0.87489600	-1.52736500
H	-1.94043800	-0.87484900	-1.52730400
H	-1.73806700	-3.02269700	-1.64558800
H	-1.44668900	-4.19958000	-0.36629900
H	-2.18109100	-2.63759300	0.02164600
H	3.68713000	-3.98960800	0.48177300
H	4.26676000	-2.47823100	-0.26810200
H	3.86911200	-2.49777200	1.44309400
H	-1.44651900	4.19967500	-0.36628200
H	-1.73796700	3.02279900	-1.64555900
H	-2.18098600	2.63772500	0.02168400
H	4.26684900	2.47811800	-0.26813500
H	3.68728200	3.98949300	0.48179300
H	3.86922700	2.49762000	1.44306600
H	-6.37538400	0.89131900	1.77367400
H	-6.37518500	-0.89179400	1.77322600
H	-6.37623400	0.00014700	0.23300800
F	3.66296900	-0.00005200	-0.85672800
F	3.30091700	-0.00005200	1.40402000

1-GGF, Charge = 0, singlet, B3LYP/6-31G(d), *E* = –1041.223045 Hartree, *ZPVE* = 0.423755 Hartree / Particle.

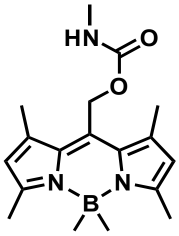

N	4.30030000	-0.62336900	1.29837200
C	-0.48570400	-1.24416800	-0.47954500
N	-1.80994200	-1.18358900	-0.01502200
B	-2.55224500	0.14905600	0.46134200
N	-1.59722300	1.34954700	0.00274200
C	-0.27935500	1.20547500	-0.47452000
C	0.26043900	-0.07164200	-0.68111400
C	-0.14108300	-2.62435700	-0.69145100
C	-1.26903400	-3.34655900	-0.34514300
C	-2.27507700	-2.44674700	0.06725900
C	-1.85508500	2.66723000	0.10510200
C	-0.72510700	3.40358600	-0.31338200
C	0.27228400	2.52187500	-0.68348900
C	1.70440500	-0.21212400	-1.11073400
C	1.12745000	-3.25843300	-1.18972700
C	-3.64475200	-2.86158700	0.50886700
C	1.60264200	2.97635600	-1.21811400
C	-3.13477900	3.29062200	0.57041200
O	2.47175600	-0.65122200	0.03360000
C	3.70109700	-0.07787200	0.20725000
O	4.17982600	0.77896300	-0.51675300
H	-0.66663700	4.48468900	-0.34057300
H	-1.37780100	-4.42366000	-0.37918400
C	5.57346600	-0.14533200	1.80301700
C	-3.98335200	0.27456500	-0.30805800
C	-2.60842800	0.13973200	2.09606800
H	3.74862800	-1.26386900	1.85082700
H	1.80568900	-0.96571900	-1.89290800
H	2.12976800	0.71184000	-1.48135700
H	2.00885900	-2.92384900	-0.63552900
H	1.05484300	-4.34575000	-1.08543300
H	1.30700900	-3.05260100	-2.25369000
H	-3.62703200	-3.91944300	0.78880200
H	-4.00551500	-2.28455400	1.36237000
H	-4.37784100	-2.74623800	-0.29861300
H	1.75264400	2.68105600	-2.26478700
H	1.64468600	4.06988700	-1.18740600
H	2.45877000	2.59536500	-0.65298900
H	-3.58610600	2.75406100	1.40645700
H	-2.93928300	4.32081100	0.88379600
H	-3.87687400	3.32678500	-0.23635300
H	6.23886300	-0.98694100	2.02298300
H	5.45251700	0.45784900	2.71202700
H	-4.66905800	-0.54135600	-0.05675700
H	-4.52333900	1.19203600	-0.05204400
H	-3.86489000	0.26761800	-1.40103900
H	-3.19634600	-0.69705400	2.49542700
H	-1.60336300	0.05553500	2.53250700
H	-3.05840000	1.04956600	2.51438600
H	6.02668000	0.47489700	1.02877800

2-OH, Charge = +1, singlet, B3LYP/6-31G(d), *E* = –1125.432801Hartree, *ZPVE* = 0.376292 Hartree / Particle.

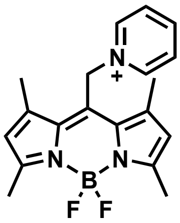

C	-0.36797400	1.23187300	-0.73081200
N	-1.61447200	1.23873400	-0.09612000
B	-2.07032900	0.00033900	0.73885700
N	-1.61495800	-1.23831700	-0.09598800
C	-0.36847100	-1.23199700	-0.73071500
C	0.27020800	-0.00020200	-0.96259500
C	-0.05027400	2.59162000	-1.09143100
C	-1.12729100	3.35345600	-0.66791100
C	-2.06862500	2.50408500	-0.03600200
C	-2.06958200	-2.50349100	-0.03570700
C	-1.12860900	-3.35328400	-0.66758900
C	-0.05131800	-2.59190900	-1.09122700
F	-1.32500600	0.00026200	1.93082700
F	-3.42437100	0.00063100	0.95605700
C	1.72947300	-0.00050000	-1.31163900
C	1.12856800	3.14487400	-1.84162200
C	-3.34894400	2.89373100	0.62278100
C	1.12725000	-3.14566400	-1.84148200
C	-3.34999400	-2.89259400	0.62321500
N	2.60766200	-0.00037600	-0.06604900
C	3.95005200	-0.00047000	-0.26414200
C	4.82888700	-0.00039400	0.80198900
C	4.31753700	-0.00022700	2.10403800
C	2.93709100	-0.00013400	2.28822200
C	2.09184700	-0.00020200	1.18557700
H	-1.24807600	4.42059000	-0.80186800
H	-1.24982600	-4.42038100	-0.80145800
H	2.02154900	0.88304700	-1.87479800
H	2.02127700	-0.88437100	-1.87443100
H	1.24324800	2.69137600	-2.83441200
H	2.07547200	3.01934700	-1.29992500
H	0.99597300	4.21875700	-1.99781900
H	-3.52453300	3.96477800	0.50067700
H	-3.32719700	2.66256200	1.69399000
H	-4.19310500	2.34057800	0.20053400
H	2.07418000	-3.02086300	-1.29966300
H	1.24228300	-2.69195500	-2.83413100
H	0.99401800	-4.21942200	-1.99800200
H	-3.32777100	-2.66197900	1.69454300
H	-3.52632200	-3.96346900	0.50066200
H	-4.19389500	-2.33865800	0.20149700
H	4.27970900	-0.00061200	-1.29692000
H	5.89580200	-0.00046900	0.60951100
H	4.98828700	-0.00017000	2.95739200
H	2.49557000	-0.00000600	3.27839000
H	1.01280800	-0.00011800	1.28941600

Charge = 0, singlet, B3LYP/6-31G(d), *E* = –876.702216 Hartree, *ZPVE* = 0.269032 Hartree / Particle.

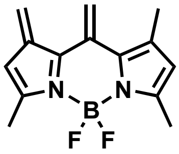

C	1.30072500	0.78375400	0.07270700
N	1.18808300	-0.60143500	-0.01490500
B	-0.08655100	-1.41252700	0.22079100
N	-1.29489200	-0.36457300	0.08408000
C	-1.16642500	0.94494300	0.05215900
C	0.12016900	1.59656600	0.29548800
C	2.64360800	1.13862000	-0.10407300
C	3.34429700	-0.07352400	-0.29420300
C	2.43735500	-1.12271600	-0.22674500
C	-2.67354500	-0.73175300	-0.05529300
C	-3.41247600	0.38844600	-0.21429200
C	-2.50246200	1.52714800	-0.23173600
C	0.12393900	2.86809500	0.78651300
C	3.24412700	2.51378700	-0.13169800
C	2.68794200	-2.59420800	-0.34306500
C	-2.76939400	2.78548600	-0.63265200
C	-3.08628600	-2.16193900	0.00467800
H	-4.48100700	0.43824700	-0.37229600
H	4.40766900	-0.17362300	-0.47497600
H	1.04770500	3.36107000	1.05969100
H	-0.79307000	3.39882200	1.00334800
H	3.37807400	2.93543300	0.87515700
H	4.23313500	2.48992500	-0.60137200
H	2.62556700	3.22325200	-0.69648200
H	3.76024000	-2.77983800	-0.45433700
H	2.33777300	-3.13189600	0.54637700
H	2.16681700	-3.02786900	-1.20326400
H	-1.99139700	3.52986700	-0.75407700
H	-3.78432300	3.08253600	-0.88199400
H	-2.70617900	-2.62842900	0.92007300
H	-4.17644200	-2.23827300	-0.01416800
H	-2.66420000	-2.72489100	-0.83259700
F	-0.16359100	-1.97710100	1.48517000
F	-0.27300600	-2.36799600	-0.77116800

Charge = 0, singlet, B3LYP/6-31G(d), *E* = –756.664572 Hartree, *ZPVE* = 0.338564 Hartree / Particle.

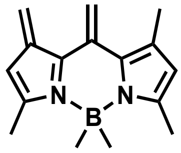

C	1.29780000	0.78010000	0.04060000
N	1.20600000	-0.60730000	-0.06110000
B	-0.10190000	-1.45380000	0.19760000
N	-1.32010000	-0.37920000	0.06750000
C	-1.16580000	0.93030000	0.03440000
C	0.11660000	1.57580000	0.29250000
C	2.63170000	1.18020000	-0.11250000
C	3.36340000	-0.00730000	-0.28980000
C	2.48260000	-1.08310000	-0.24080000
C	-2.72750000	-0.68070000	-0.04850000
C	-3.42400000	0.46640000	-0.20510000
C	-2.47530000	1.56500000	-0.24480000
C	0.11530000	2.82980000	0.82750000
C	3.19800000	2.57080000	-0.12730000
C	2.88300000	-2.52540000	-0.34540000
C	-2.69620000	2.82830000	-0.65800000
C	-3.29300000	-2.06090000	0.01580000
H	-4.49310000	0.55050000	-0.34510000
H	4.43320000	-0.08470000	-0.44450000
C	-0.31310000	-2.57330000	-0.97040000
C	-0.10800000	-2.02770000	1.72500000
H	1.03670000	3.31810000	1.11570000
H	-0.80410000	3.34610000	1.06760000
H	3.32590000	2.98810000	0.88240000
H	4.18670000	2.57330000	-0.59900000
H	2.56350000	3.27110000	-0.68500000
H	3.96290000	-2.60260000	-0.18250000
H	2.38950000	-3.15910000	0.39820000
H	2.67330000	-2.95580000	-1.33290000
H	-1.88920000	3.53880000	-0.79280000
H	-3.70100000	3.16270000	-0.90140000
H	-2.86930000	-2.63810000	0.84150000
H	-4.37540000	-1.99180000	0.15840000
H	-3.10550000	-2.62020000	-0.90560000
H	0.59120000	-3.16980000	-1.11040000
H	-1.10130000	-3.29700000	-0.73770000
H	-0.54560000	-2.13030000	-1.94950000
H	0.72380000	-2.72800000	1.87680000
H	0.01450000	-1.22780000	2.46780000
H	-1.02240000	-2.57760000	1.98720000

Charge = **+1**, **singlet**, BS-B3LYP/6-31G(d), *E* = –877.078687 Hartree, *ZPVE* = 0.278750 Hartree / Particle.

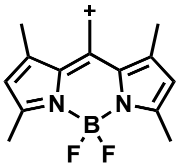

C	1.25380000	0.83270000	0.02320000
N	1.24830000	-0.52870000	0.01500000
B	0.00000000	-1.47890000	0.10890000
N	-1.24830000	-0.52870000	0.01490000
C	-1.25380000	0.83270000	0.02330000
C	0.00000000	1.57430000	0.11840000
C	2.64060000	1.29930000	-0.07730000
C	3.40570000	0.15850000	-0.12940000
C	2.54530000	-0.97320000	-0.06610000
C	-2.54530000	-0.97320000	-0.06630000
C	-3.40560000	0.15850000	-0.12950000
C	-2.64060000	1.29930000	-0.07710000
F	0.00000000	-2.33750000	-0.96890000
F	-0.00010000	-2.13830000	1.31630000
C	0.00010000	2.91800000	0.34000000
C	3.16700000	2.69890000	-0.14310000
C	2.89930000	-2.41040000	-0.07810000
C	-3.16710000	2.69890000	-0.14260000
C	-2.89920000	-2.41040000	-0.07860000
H	4.48310000	0.10810000	-0.21290000
H	-4.48310000	0.10810000	-0.21300000
H	0.91670000	3.47970000	0.44720000
H	-0.91650000	3.47970000	0.44730000
H	3.03220000	3.23420000	0.80560000
H	2.68430000	3.28490000	-0.93360000
H	4.23920000	2.68060000	-0.35260000
H	3.98180000	-2.54590000	-0.08670000
H	2.45980000	-2.90160000	-0.95540000
H	2.47080000	-2.91290000	0.79810000
H	-4.23930000	2.68060000	-0.35200000
H	-2.68460000	3.28500000	-0.93320000
H	-3.03230000	3.23410000	0.80610000
H	-2.46020000	-2.90130000	-0.95620000
H	-3.98170000	-2.54600000	-0.08680000
H	-2.47040000	-2.91320000	0.79740000

Charge = **0**, **doublet**, UB3LYP/6-31G(d), *E* = –877.325711 Hartree, *ZPVE* = 0.279344 Hartree / Particle.

C	1.24412900	0.85671600	0.00001200
N	1.24553800	-0.52991200	0.00016300
B	-0.00000200	-1.46168700	-0.00036700
N	-1.24553800	-0.52990500	0.00016200
C	-1.24412600	0.85672600	0.00001400
C	0.00000800	1.57752700	-0.00006500
C	2.60625800	1.29978200	0.00009300
C	3.38474200	0.14411400	0.00023500
C	2.52953300	-0.96996600	0.00032600
C	-2.52953200	-0.96996000	0.00030000
C	-3.38474300	0.14411600	0.00020000
C	-2.60626400	1.29978600	0.00012300
F	-0.00000500	-2.25595300	-1.14563200
F	-0.00000500	-2.25713900	1.14411600
C	0.00003600	2.97230500	-0.00018400
C	3.17753400	2.68889200	0.00000700
C	2.88515600	-2.41945000	0.00036900
C	-3.17756500	2.68888700	0.00008300
C	-2.88515000	-2.41944600	0.00031600
H	4.46660600	0.10214600	0.00034700
H	-4.46660700	0.10214300	0.00028900
H	0.91775100	3.53926400	-0.00022000
H	-0.91764700	3.53930800	-0.00022400
H	2.88102300	3.26587900	0.88514300
H	2.88104400	3.26574100	-0.88522400
H	4.27058700	2.63679800	0.00002500
H	3.97085600	-2.54607300	0.00078900
H	2.46382400	-2.92058800	-0.87793200
H	2.46305600	-2.92077700	0.87817200
H	-4.27061700	2.63676900	0.00008900
H	-2.88108100	3.26577200	-0.88512500
H	-2.88107900	3.26584800	0.88524400
H	-2.46378300	-2.92057300	-0.87797400
H	-3.97084900	-2.54607200	0.00069500
H	-2.46308000	-2.92077900	0.87813000

Charge = **+1**, **singlet**, BS-B3LYP/6-31G(d), *E* = –757.104149 Hartree, *ZPVE* = 0.348252 Hartree / Particle.

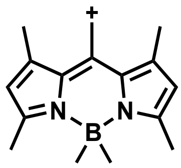

C	1.24890000	0.81650000	0.02700000
N	1.27110000	-0.54440000	0.00890000
B	0.00000000	-1.53290000	0.16830000
N	-1.27110000	-0.54440000	0.00890000
C	-1.24890000	0.81650000	0.02690000
C	0.00000000	1.54210000	0.20270000
C	2.60900000	1.34260000	-0.13620000
C	3.40850000	0.23390000	-0.21500000
C	2.59060000	-0.92830000	-0.11030000
C	-2.59060000	-0.92830000	-0.11030000
C	-3.40850000	0.23390000	-0.21510000
C	-2.60900000	1.34260000	-0.13630000
C	0.00000000	2.84450000	0.59870000
C	3.06950000	2.76280000	-0.24750000
C	3.10060000	-2.32260000	-0.13240000
C	-3.10070000	-2.32260000	-0.13230000
H	-4.48280000	0.21600000	-0.34350000
H	4.48280000	0.21600000	-0.34340000
C	0.00000000	-2.58410000	-1.06530000
C	0.00000000	-2.13630000	1.67370000
H	0.91670000	3.38410000	0.78830000
H	-0.91670000	3.38410000	0.78800000
H	3.05600000	3.27870000	0.72160000
H	4.10120000	2.78800000	-0.60740000
H	2.45670000	3.34330000	-0.94540000
H	4.14970000	-2.33130000	0.17560000
H	2.52960000	-2.99120000	0.51290000
H	3.05580000	-2.73020000	-1.15190000
H	-2.52960000	-2.99120000	0.51290000
H	-4.14970000	-2.33130000	0.17580000
H	-3.05600000	-2.73020000	-1.15190000
H	0.86510000	-3.25290000	-1.04570000
H	-0.86510000	-3.25290000	-1.04560000
H	-0.00010000	-2.08840000	-2.04590000
H	0.87320000	-2.77000000	1.87190000
H	0.00000000	-1.35030000	2.44120000
H	-0.87310000	-2.77010000	1.87180000
C	-3.06950000	2.76280000	-0.24750000
H	-3.05600000	3.27870000	0.72170000
H	-2.45660000	3.34340000	-0.94530000
H	-4.10120000	2.78810000	-0.60750000

Charge = **0**, **doublet**, UB3LYP/6-31G(d), *E* = –757.341640 Hartree, *ZPVE* = 0.349049 Hartree / Particle.

C	1.24505000	0.84357500	0.00100400
N	1.27214200	-0.54675500	-0.02217200
B	0.00003100	-1.50960700	0.11867800
N	-1.27213900	-0.54676300	-0.02203800
C	-1.24504900	0.84357300	0.00099900
C	0.00000100	1.55182700	0.06636100
C	2.59058300	1.33578500	-0.06059500
C	3.39624100	0.20834100	-0.10947300
C	2.57461300	-0.93355800	-0.07946700
C	-2.57459000	-0.93357800	-0.07929200
C	-3.39623300	0.20831800	-0.10951900
C	-2.59060400	1.33576800	-0.06063000
C	-0.00002900	2.94062100	0.21723000
C	3.12859900	2.73863800	-0.09985500
C	3.08267400	-2.34046200	-0.12033000
C	-3.08266900	-2.34048100	-0.12002500
H	-4.47759900	0.19352900	-0.16804900
H	4.47760900	0.19358800	-0.16797100
C	-0.00008100	-2.58054400	-1.10961000
C	0.00010600	-2.13958200	1.62643900
H	0.91617600	3.50308000	0.29523600
H	-0.91627000	3.50300500	0.29534200
H	2.94724300	3.28981900	0.83233300
H	4.21296800	2.70825100	-0.24608300
H	2.70680700	3.33089800	-0.92094600
H	4.13808700	-2.34821200	0.16945800
H	2.53585200	-3.00631400	0.55021800
H	3.01516000	-2.76522000	-1.12948400
H	-2.53580400	-3.00630000	0.55051500
H	-4.13806300	-2.34820200	0.16983200
H	-3.01522800	-2.76530200	-1.12915900
H	0.86894700	-3.24616500	-1.09063100
H	-0.86902900	-3.24626500	-1.09039900
H	-0.00023500	-2.08282700	-2.09002800
H	0.87582100	-2.77155900	1.82477800
H	0.00029200	-1.35207800	2.39324500
H	-0.87577200	-2.77128300	1.82493700
C	-3.12862500	2.73864000	-0.10012200
H	-2.94745700	3.28992000	0.83204000
H	-2.70664700	3.33078700	-0.92119700
H	-4.21295900	2.70822500	-0.24658600

Charge = 0, singlet, B3LYP/6-31G(d), *E* = –284.440087 Hartree, *ZPVE* = 0.080057 Hartree / Particle.

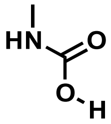

N	-0.62800300	-0.65686700	-0.07685700
O	1.65888300	-0.73344600	0.03507700
C	0.61473400	-0.10695600	-0.00793600
O	0.56481700	1.25779700	-0.01362200
C	-1.88369300	0.06782700	0.03600200
H	-0.62913900	-1.65998300	0.03403600
H	1.49643800	1.53592900	0.00661900
H	-2.12814200	0.33240200	1.07374100
H	-2.68679600	-0.55648000	-0.36490600
H	-1.83219100	0.98616000	-0.55152700

Charge = **–1**, **singlet**, B3LYP/6-31G(d), *E* = –283.859133 Hartree, *ZPVE* = 0.066482 Hartree / Particle.

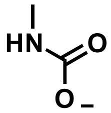

N	0.59672700	-0.64806200	0.26712900
O	-1.65598500	-0.69733800	-0.11724100
C	-0.67256400	0.06750600	0.02997000
O	-0.59808900	1.32047500	0.03653300
C	1.82262600	0.01449500	-0.11441800
H	0.50202900	-1.59218800	-0.09265900
H	2.07378700	-0.03921500	-1.19602200
H	2.69350700	-0.38012200	0.43859000
H	1.68580400	1.07085800	0.13253600

Charge = **0**, **doublet**, UB3LYP/6-31G(d), *E* = –283.772941 Hartree, *ZPVE* = 0.066206 Hartree / Particle.

N	0.62175500	-0.62814500	0.00368400
O	-1.68400400	-0.64957400	-0.00114900
C	-0.58000100	-0.01928100	0.00019800
O	-0.74272500	1.23904800	0.00019200
C	1.88511900	0.09359400	-0.00146200
H	0.61475100	-1.63883600	-0.00707500
H	2.45326400	-0.10998100	-0.91585600
H	2.49302400	-0.17833500	0.86796700
H	1.66979200	1.16249300	0.04442100

Charge = **0**, **singlet**, B3LYP/6-31G(d), *E* = –248.284961 Hartree, *ZPVE* = 0.089021 Hartree / Particle.

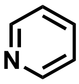

C	1.14236400	-0.72169300	-0.00007800
C	1.19860900	0.67315200	0.00002400
C	-0.00020600	1.38568000	-0.00001200
C	-1.19879000	0.67285500	-0.00002100
C	-1.14214000	-0.72202800	0.00005600
H	2.05980500	-1.30874800	0.00011200
H	2.15767600	1.18291200	0.00012100
H	-0.00030700	2.47255000	0.00001100
H	-2.15803300	1.18228200	-0.00004600
H	-2.05945800	-1.30928400	-0.00000300
N	0.00018500	-1.42107200	-0.00000200

Charge = **+1**, **doublet**, UB3LYP/6-31G(d), *E* = –247.958502 Hartree, *ZPVE* = 0.086620 Hartree / Particle.

C	-1.20183300	-0.74882400	0.00001200
C	-1.21656400	0.65668400	0.00007600
C	-0.00025900	1.34379700	-0.00006700
C	1.21629000	0.65706000	0.00005300
C	1.20215400	-0.74844100	0.00017400
H	-2.08179300	-1.38726600	0.00025800
H	-2.17762500	1.16196100	-0.00005100
H	-0.00038400	2.43035500	-0.00002300
H	2.17712800	1.16276100	-0.00032500
H	2.08239600	-1.38645300	0.00006400
N	0.00022200	-1.27757400	-0.00020200

Charge = **–1**, **singlet**, B3LYP/6-31G(d), *E* = –832.609067 Hartree, *ZPVE* = 0.352939 Hartree / Particle.

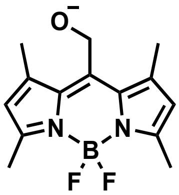

C	1.22769000	0.64096500	-0.13630200
N	1.27087500	-0.75887300	-0.06636000
B	0.00033200	-1.71069300	0.04921600
N	-1.27055500	-0.75925400	-0.06656700
C	-1.22770000	0.64056300	-0.13614700
C	-0.00008900	1.33545600	-0.28409200
C	2.55431300	1.14592800	-0.06682900
C	3.38251900	0.02467500	0.01305100
C	2.57987200	-1.12380200	-0.00773700
C	-2.57934100	-1.12460400	-0.00777800
C	-3.38235700	0.02365200	0.01361000
C	-2.55442400	1.14516100	-0.06650500
C	-0.00059400	2.82599400	-0.54491000
C	2.99745900	2.57632100	0.06352000
C	3.10388700	-2.52934400	-0.02748900
C	-2.99835000	2.57530400	0.06347600
C	-3.10266700	-2.53041200	-0.02823000
O	-0.00114900	3.33260100	0.71614000
H	-4.46371200	0.02466400	0.09994900
H	4.46390800	0.02596400	0.09907600
C	0.00032900	-2.79487800	-1.17772800
C	0.00010200	-2.36453100	1.55847600
H	0.87955300	3.08934200	-1.17737900
H	-0.88067300	3.08850300	-1.17766500
H	2.22661900	3.15659700	0.58690900
H	3.94016700	2.62643800	0.62473200
H	3.17781700	3.05205000	-0.91128800
H	4.15569700	-2.52249800	0.28096600
H	2.55847100	-3.20086300	0.64052200
H	3.06024400	-2.97145500	-1.03202200
H	-3.17983300	3.05037700	-0.91142600
H	-3.94056300	2.62520800	0.62554300
H	-2.22733100	3.15642500	0.58570200
H	-2.55345200	-3.20312100	0.63534800
H	-4.15297100	-2.52505000	0.28536900
H	-3.06375400	-2.97000800	-1.03413600
H	0.87313100	-3.45992700	-1.15507000
H	-0.87566800	-3.45581700	-1.15900300
H	0.00360100	-2.29704700	-2.15870200
H	0.87630700	-2.99681200	1.75741100
H	-0.00005300	-1.57523000	2.32294800
H	-0.87610600	-2.99688700	1.75718000

Charge = **0**, **doublet**, UB3LYP/6-31G(d), *E* = –832.527543 Hartree, *ZPVE* = 0.352154 Hartree / Particle.

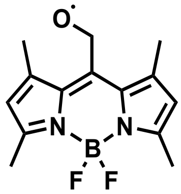

C	1.22873100	0.62157500	-0.10850400
N	1.26783000	-0.78300500	-0.05705400
B	-0.01311200	-1.73436900	0.09081500
N	-1.28103600	-0.76545300	-0.05429100
C	-1.22407500	0.63864400	-0.10869600
C	0.00684200	1.30131000	-0.17425000
C	2.57943100	1.11744200	-0.09854000
C	3.38524700	-0.00429600	-0.05625000
C	2.56212100	-1.15460600	-0.03937600
C	-2.58001100	-1.12097300	-0.03880100
C	-3.38838500	0.03926200	-0.05771700
C	-2.56812400	1.15065900	-0.10068700
C	0.03023600	2.81095700	-0.35633700
C	3.10595100	2.52657000	-0.07826100
C	3.07974300	-2.55940600	-0.03350300
C	-3.07605500	2.56630500	-0.07322500
C	-3.11526200	-2.51925600	-0.03448700
O	0.04315100	3.54500700	0.79851500
H	-4.47098900	0.03891000	-0.02719900
H	4.46773600	-0.01863500	-0.02518900
C	-0.02112000	-2.82706200	-1.11624300
C	-0.01597100	-2.32947400	1.61465300
H	0.86885100	3.11235300	-1.00406000
H	-0.85229300	3.14057100	-0.93150100
H	2.59988100	3.15467600	0.66140400
H	4.17142600	2.51216200	0.17173500
H	3.01464200	3.02691600	-1.05202500
H	4.11503100	-2.56216900	0.32099600
H	2.49328200	-3.22373400	0.60274900
H	3.07607700	-2.98563700	-1.04437700
H	-2.96173700	3.07970000	-1.03807800
H	-4.14615400	2.56390300	0.15662800
H	-2.57412100	3.17455700	0.68586100
H	-2.53881100	-3.19099400	0.60316300
H	-4.15125900	-2.50883200	0.31785400
H	-3.11492700	-2.94561800	-1.04527700
H	0.84806200	-3.49353500	-1.08640200
H	-0.89462800	-3.48754800	-1.08073400
H	-0.02252100	-2.34814800	-2.10581400
H	0.85349700	-2.96396300	1.82845600
H	-0.00770400	-1.52193800	2.35960000
H	-0.89634700	-2.94791300	1.83077900

Charge = **+1**, **singlet**, B3LYP/6-31G(d), *E* = –208.290075 Hartree, *ZPVE* = 0.063233 Hartree / Particle.

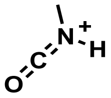

N	-0.43531100	0.54425300	-0.00063200
C	0.76228200	0.12495400	0.00078100
O	1.83098100	-0.27926800	-0.00025800
C	-1.64396000	-0.36479000	0.00006900
H	-0.57700800	1.55845300	0.00093200
H	-2.21561500	-0.14685500	-0.90258700
H	-2.21421100	-0.14801600	0.90388200
H	-1.30377000	-1.40019200	-0.00084600

Charge = **0**, **doublet**, UB3LYP/6-31G(d), *E* = –208.543219 Hartree, *ZPVE* = 0.062187 Hartree / Particle.

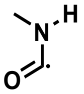

N	-0.46437400	0.64698900	0.00024300
C	0.87203100	0.50406000	-0.00017000
O	1.53195600	-0.50510100	0.00003900
C	-1.41335200	-0.46973600	-0.00005800
H	-0.82556300	1.58943800	-0.00051800
H	-2.04700200	-0.44609500	-0.89373900
H	-2.05039100	-0.44322100	0.89110100
H	-0.83414700	-1.39418300	0.00251400

1-GGF**,** sigmatropic hydrogen transfer transition state, Charge = 0, singlet, B3LYP/6-31G(d), *E* = –1041.125882 Hartree, *ZPVE* = 0.417849 Hartree / Particle.

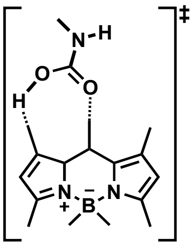

N	3.85831300	-0.74273500	1.19566400
C	-0.36922900	-1.30054300	-0.63065300
N	-1.61616200	-1.18304500	-0.02799000
B	-2.31356000	0.18404400	0.41583100
N	-1.36326100	1.34791500	-0.16537600
C	-0.12902500	1.15763500	-0.73040100
C	0.35579400	-0.12243000	-1.02587100
C	-0.04364500	-2.67436600	-0.76391900
C	-1.12995400	-3.37207800	-0.22436200
C	-2.08032500	-2.44663200	0.21241200
C	-1.54507800	2.71801600	-0.00799400
C	-0.42947100	3.41081200	-0.40976700
C	0.53930600	2.47922800	-0.88298600
C	1.55601500	-0.24345600	-1.80102500
C	1.17832600	-3.34351600	-1.33327500
C	-3.40486700	-2.80148600	0.81584100
C	1.86030200	2.79298400	-1.19547600
C	-2.81150000	3.35413600	0.46967700
O	3.03835100	-0.36688600	-0.86286500
C	3.17844100	0.04059200	0.34565600
O	2.75194300	1.16612800	0.83116700
H	-0.31205300	4.48598200	-0.39435400
H	-1.22839900	-4.44889600	-0.15332700
C	4.12694400	-0.40094800	2.58784500
C	-3.79259600	0.29687900	-0.26032500
C	-2.26773600	0.27676500	2.05050300
H	4.10856200	-1.65445200	0.84142500
H	1.72074600	-1.18847500	-2.29689600
H	1.82937900	0.60146000	-2.41657700
H	2.11770000	-2.91474700	-0.96326800
H	1.17563800	-4.40458600	-1.06120600
H	1.21776400	-3.30474100	-2.43226300
H	-3.39211400	-3.85711700	1.10628900
H	-3.64385500	-2.20879600	1.70263400
H	-4.23025800	-2.66677500	0.10560600
H	2.51529700	2.16332100	-1.78882900
H	2.13572100	3.84478000	-1.19026700
H	2.42160100	1.86111100	0.10400300
H	-3.19883800	2.88863300	1.37822300
H	-2.63060700	4.41452800	0.66822900
H	-3.59721300	3.28101700	-0.29091700
H	4.53946900	0.60831400	2.65395400
H	4.85657800	-1.11169000	2.98017200
H	-4.45874800	-0.51416400	0.05461600
H	-4.31750500	1.22112500	0.00830300
H	-3.74610800	0.26136100	-1.35840300
H	-2.86528000	-0.50477200	2.53789000
H	-1.24086000	0.16906800	2.42934900
H	-2.64872000	1.22778800	2.44616700
H	3.21510600	-0.44792200	3.19252200

10. NMR Spectra of compounds

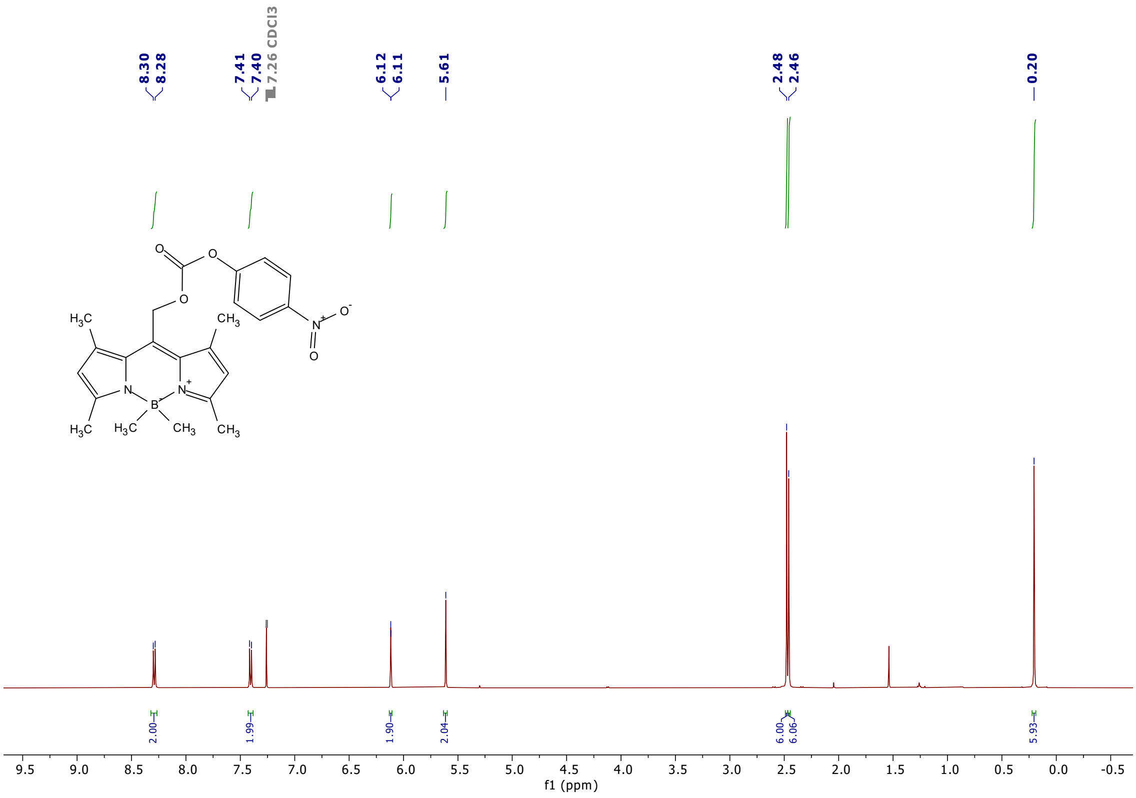

*Figure S35. ¹H NMR (500 MHz, CDCl3) of **1**-pNP*

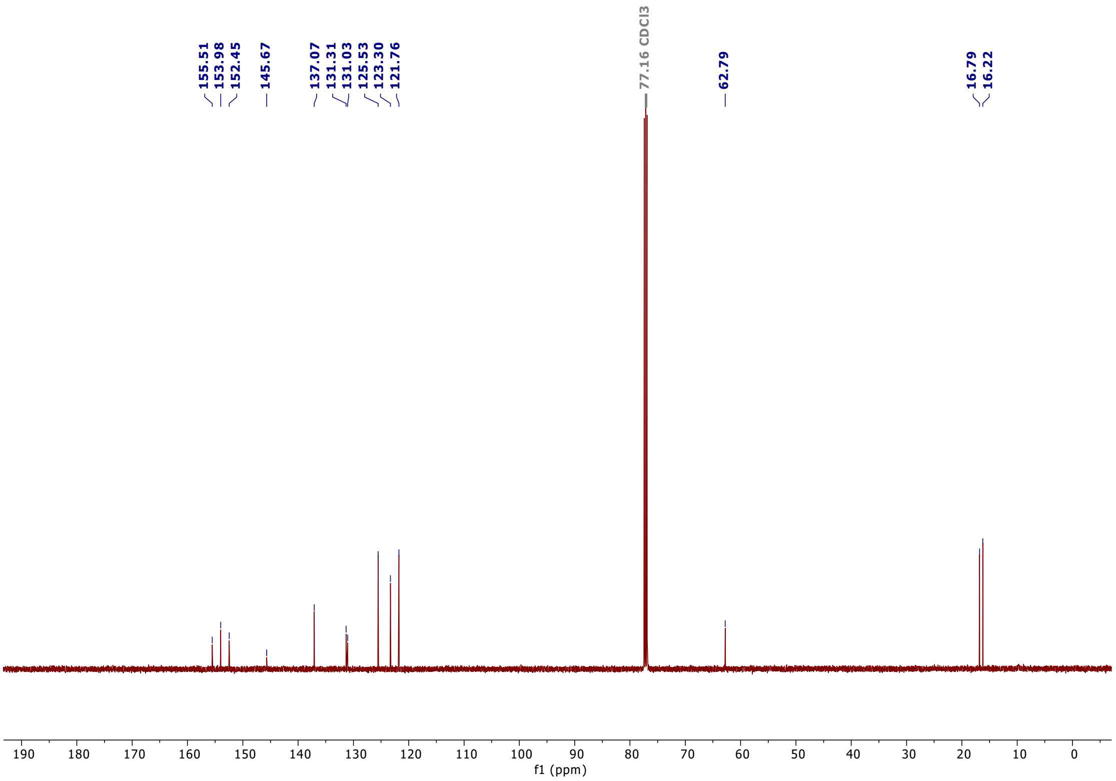

*Figure S36. ¹³C NMR (125 MHz, CDCl3) of **1**-pNP*

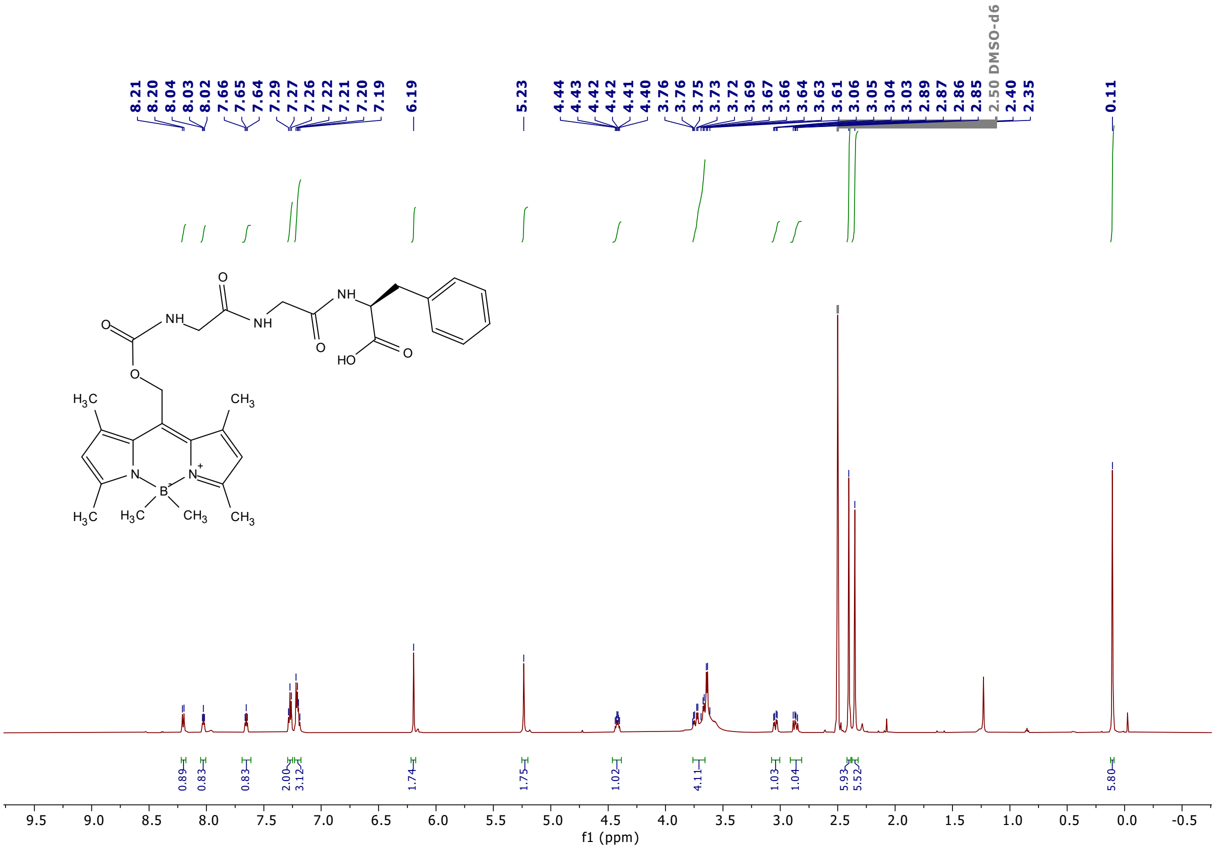

*Figure S37. 1H NMR (600 MHz, DMSO-d$_6$) of **1**-GGF*

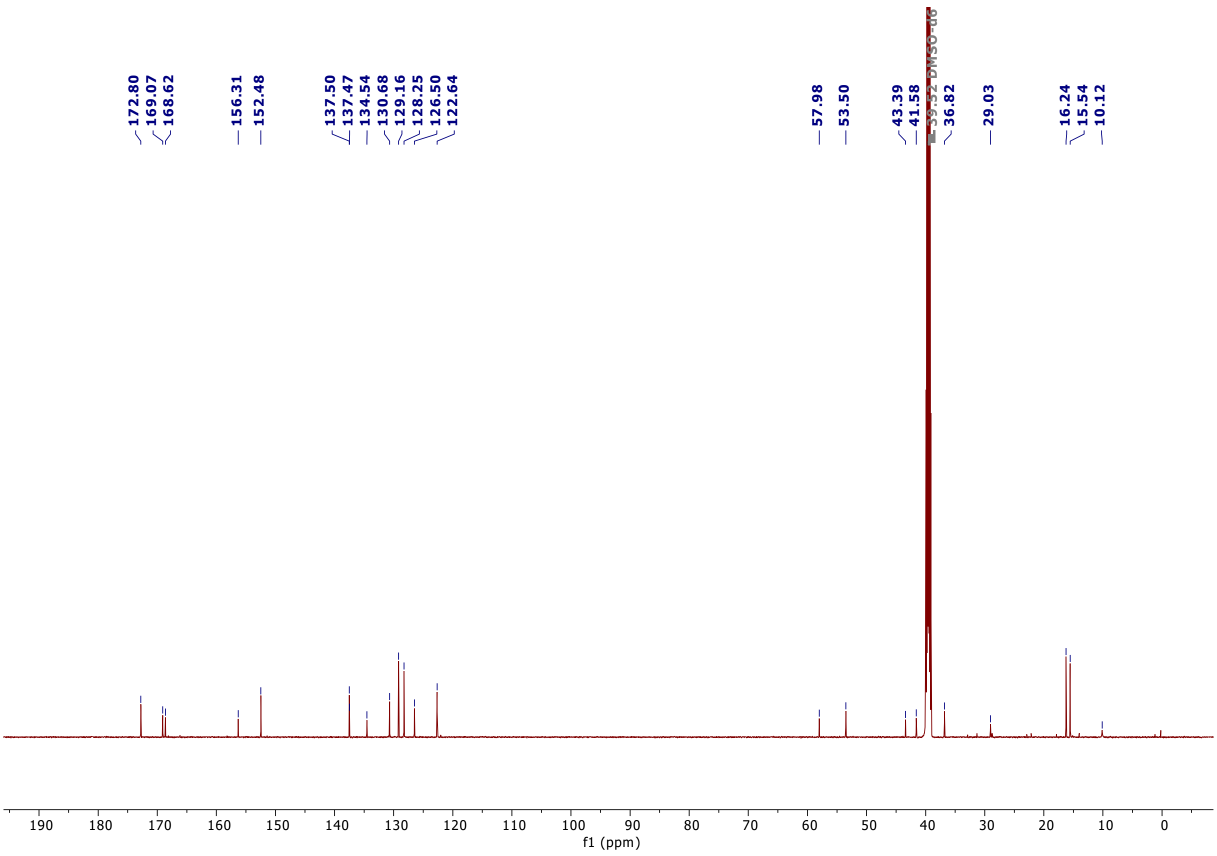

Figure S38. ^{13}C NMR (151 MHz, DMSO-d$_6$) of 1-GGF

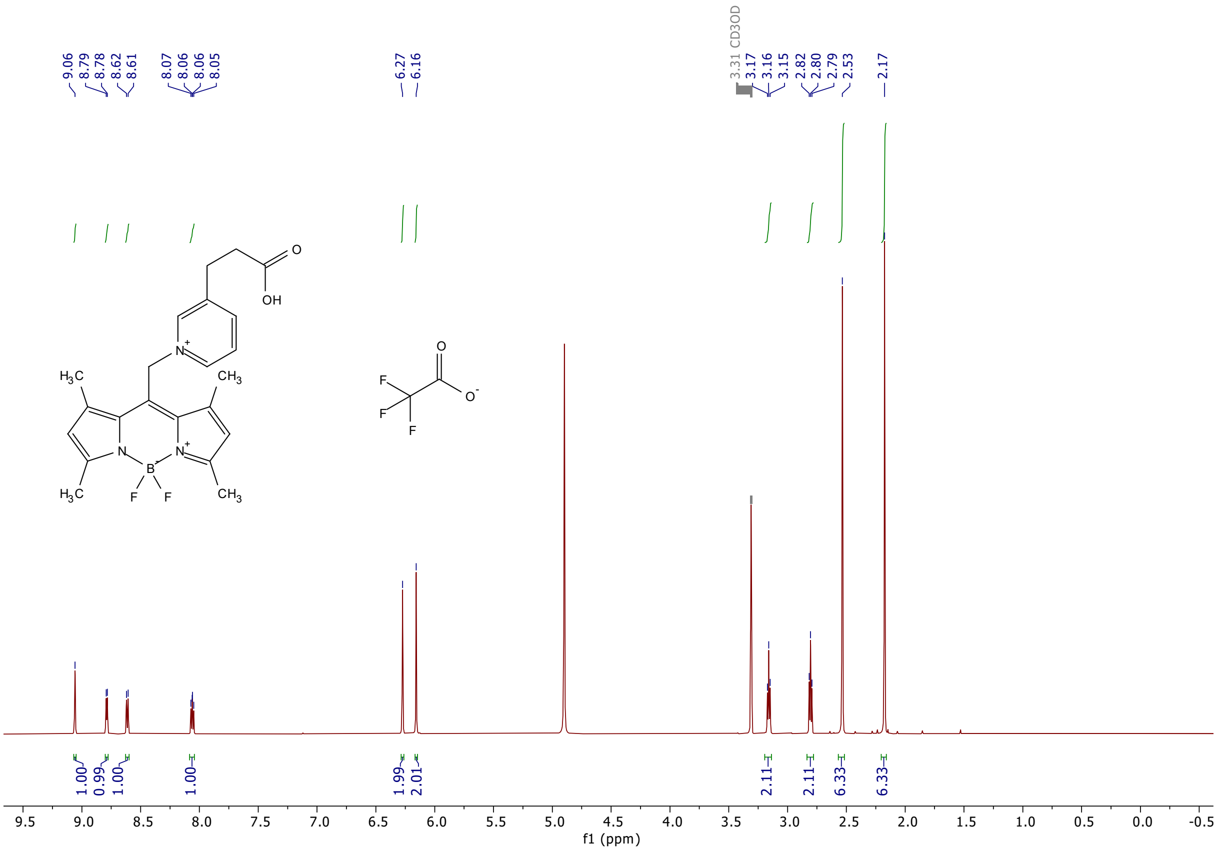

*Figure S39. 1H NMR (500 MHz, CD_3OD) of **2**-OH*

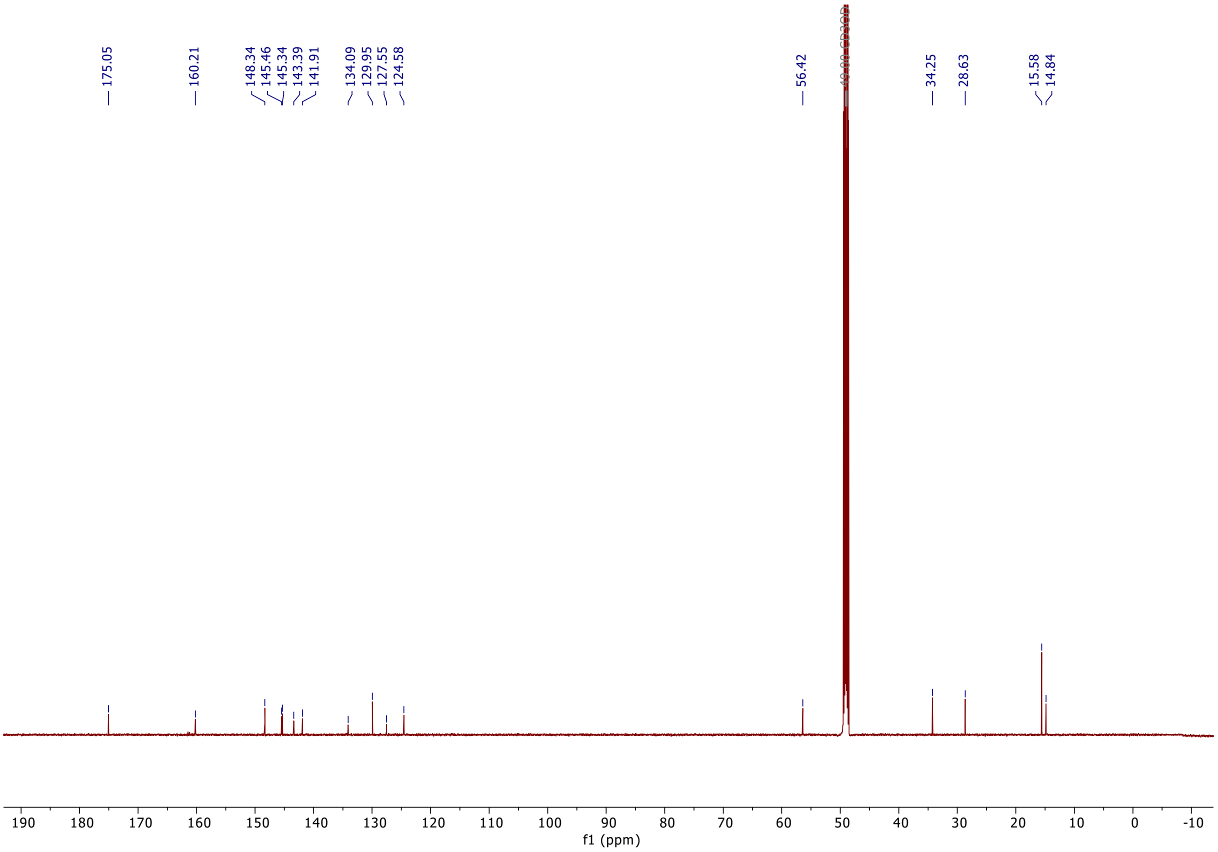

*Figure S40. ^{13}C NMR (151 MHz, CD_3OD) of **2**-OH*

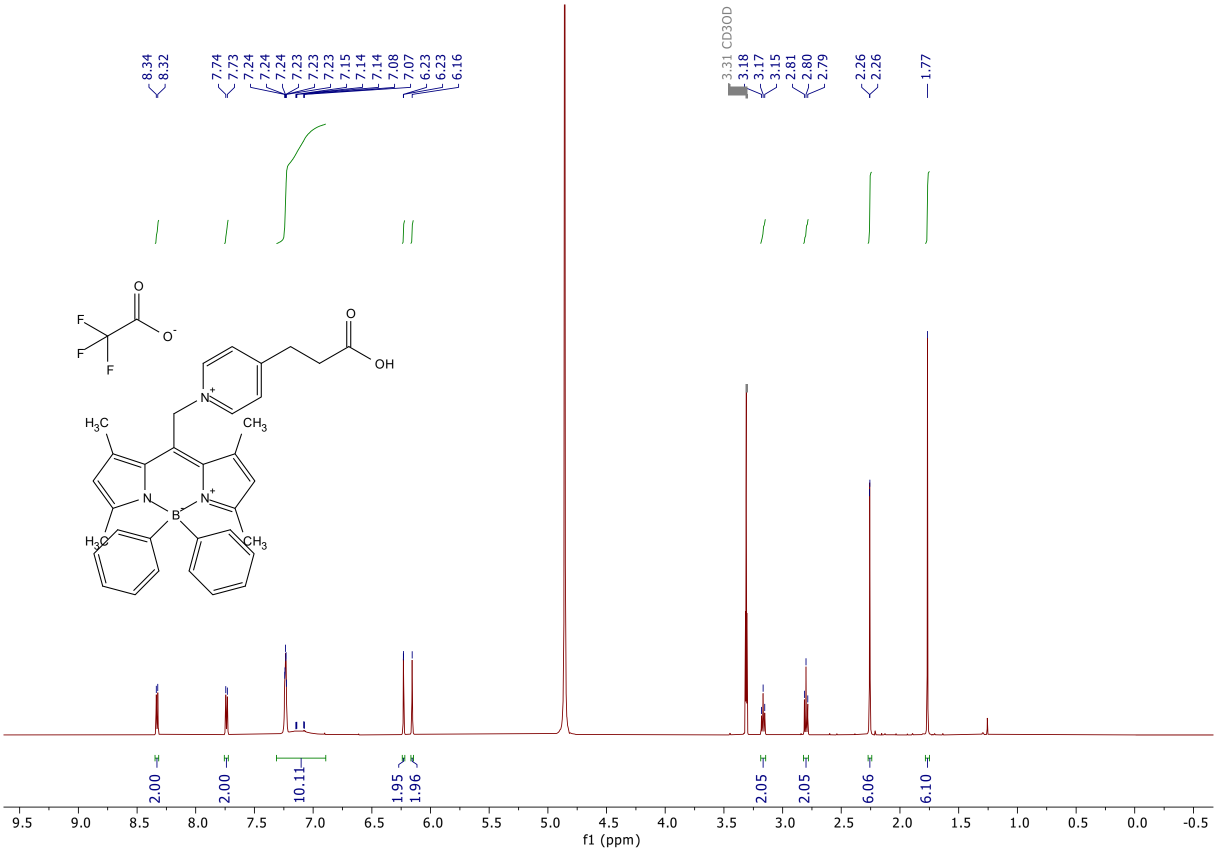

*Figure S41. ^{1}H NMR (600 MHz, CD_3OD) of **3**-OH*

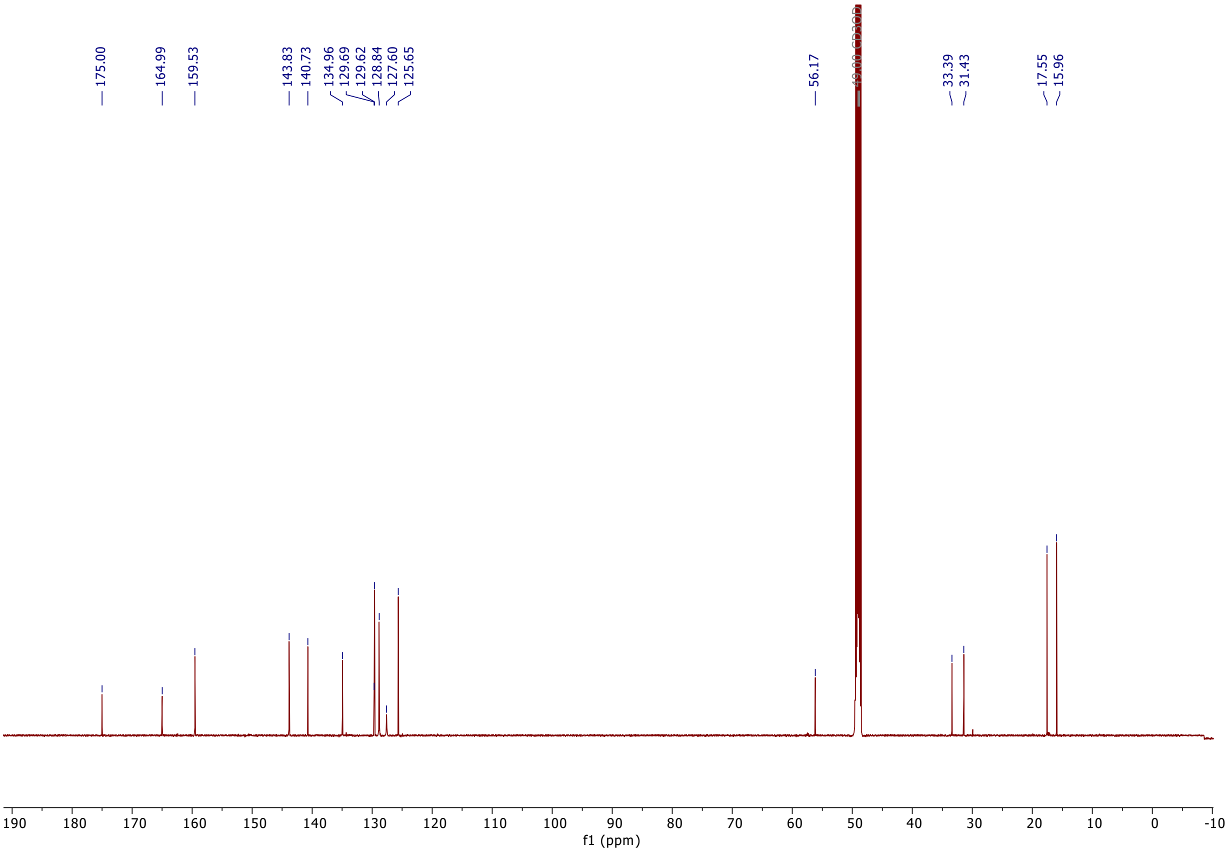

*Figure S42. ^{13}C NMR (151 MHz, CD_3OD) of **3**-OH*

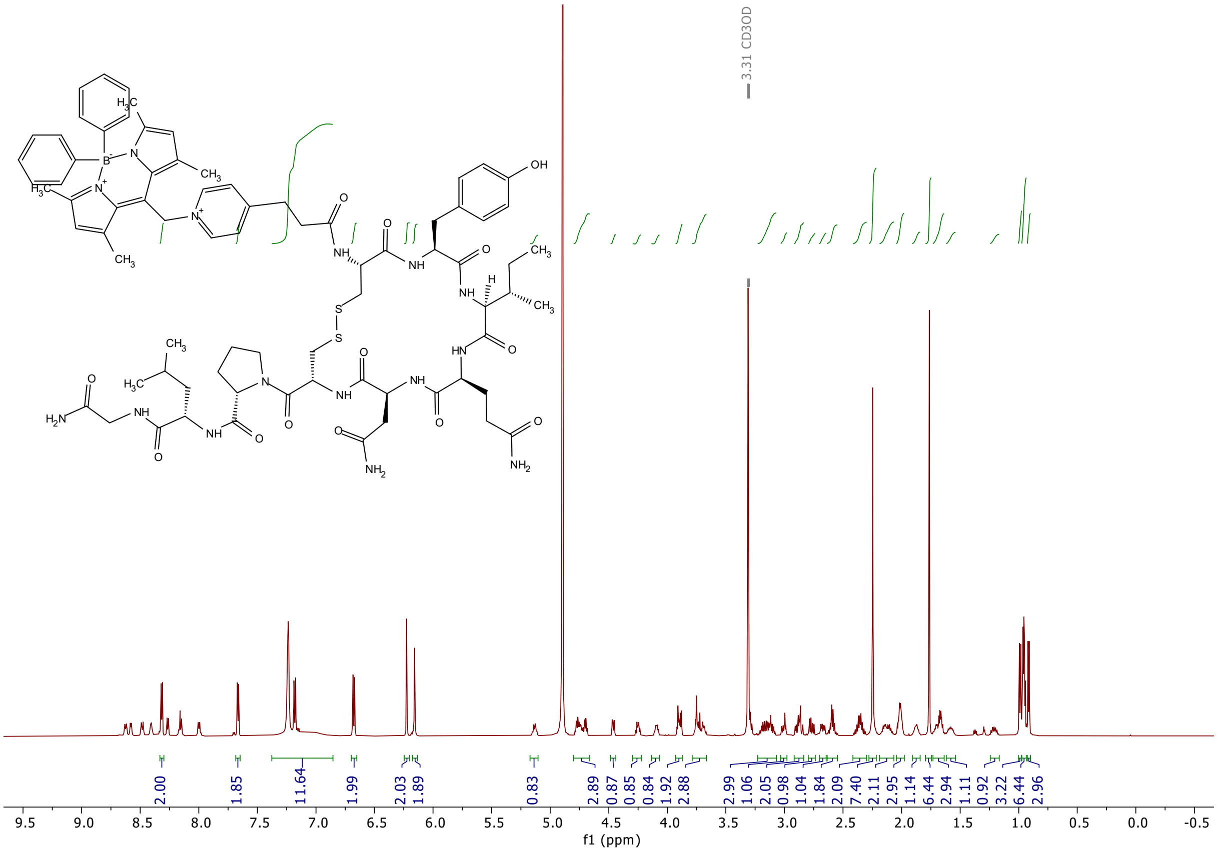

*Figure S43. ^{1}H NMR (600 MHz, CD_3OD) of **3**-oxytocin*

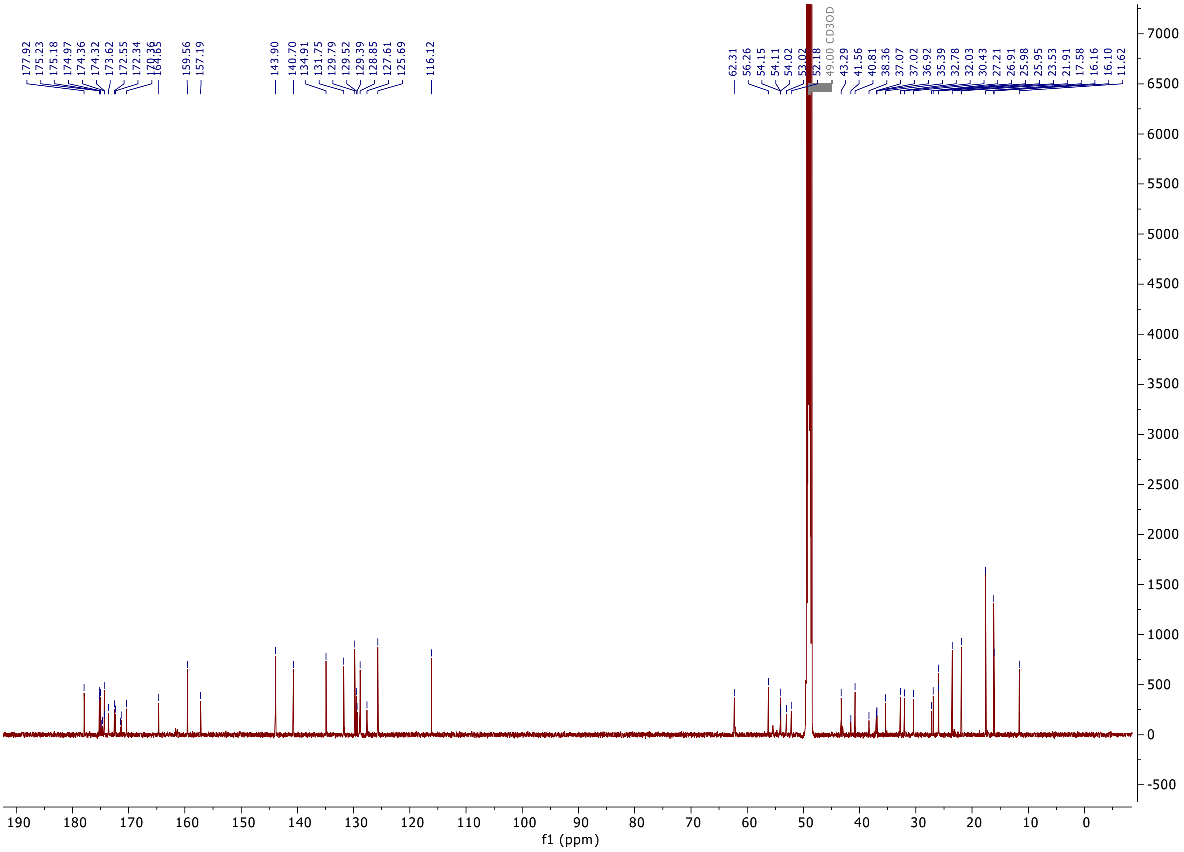

*Figure S44. ^{13}C NMR (151 MHz, CD_3OD) of **3**-oxytocin*

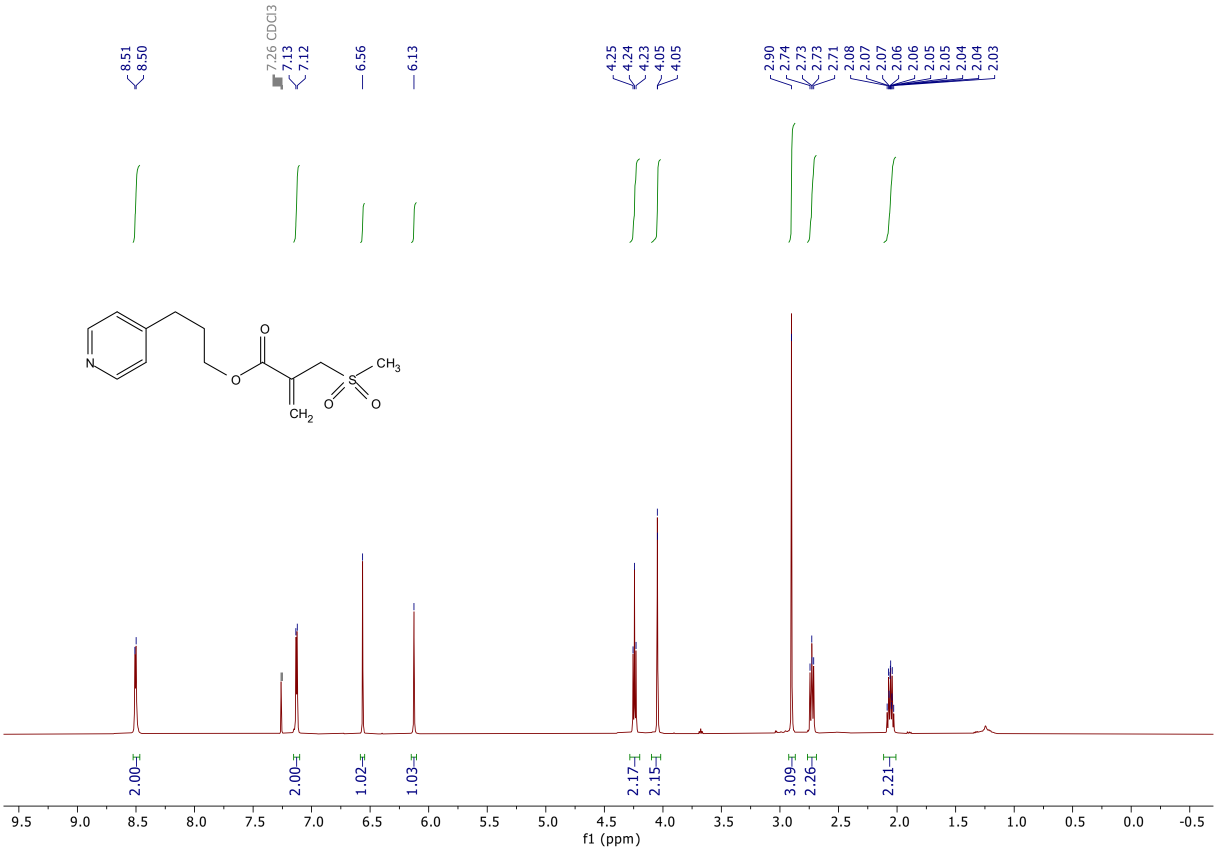

*Figure S45. ^{1}H NMR (500 MHz, $CDCl_3$) of **S6***

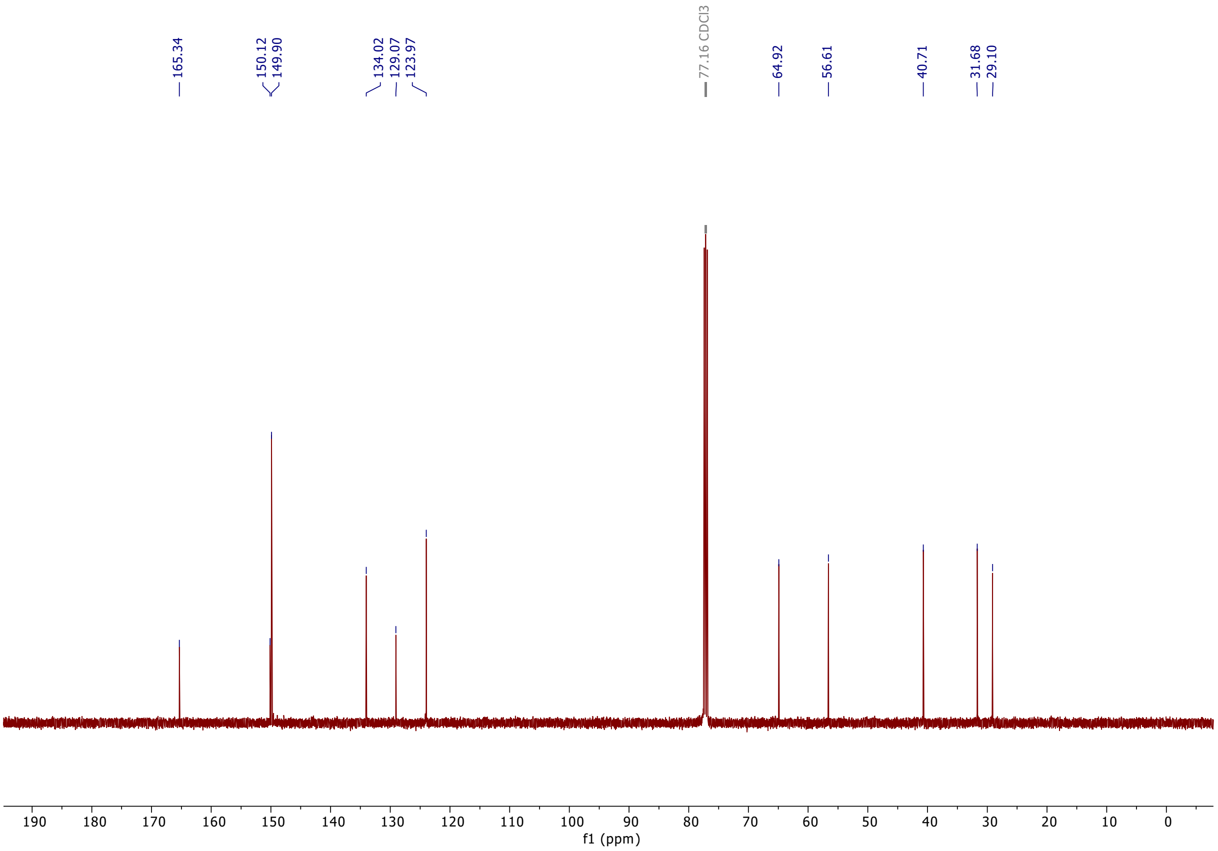

*Figure S46. ^{13}C NMR (126 MHz, CD_3OD) of **S6***

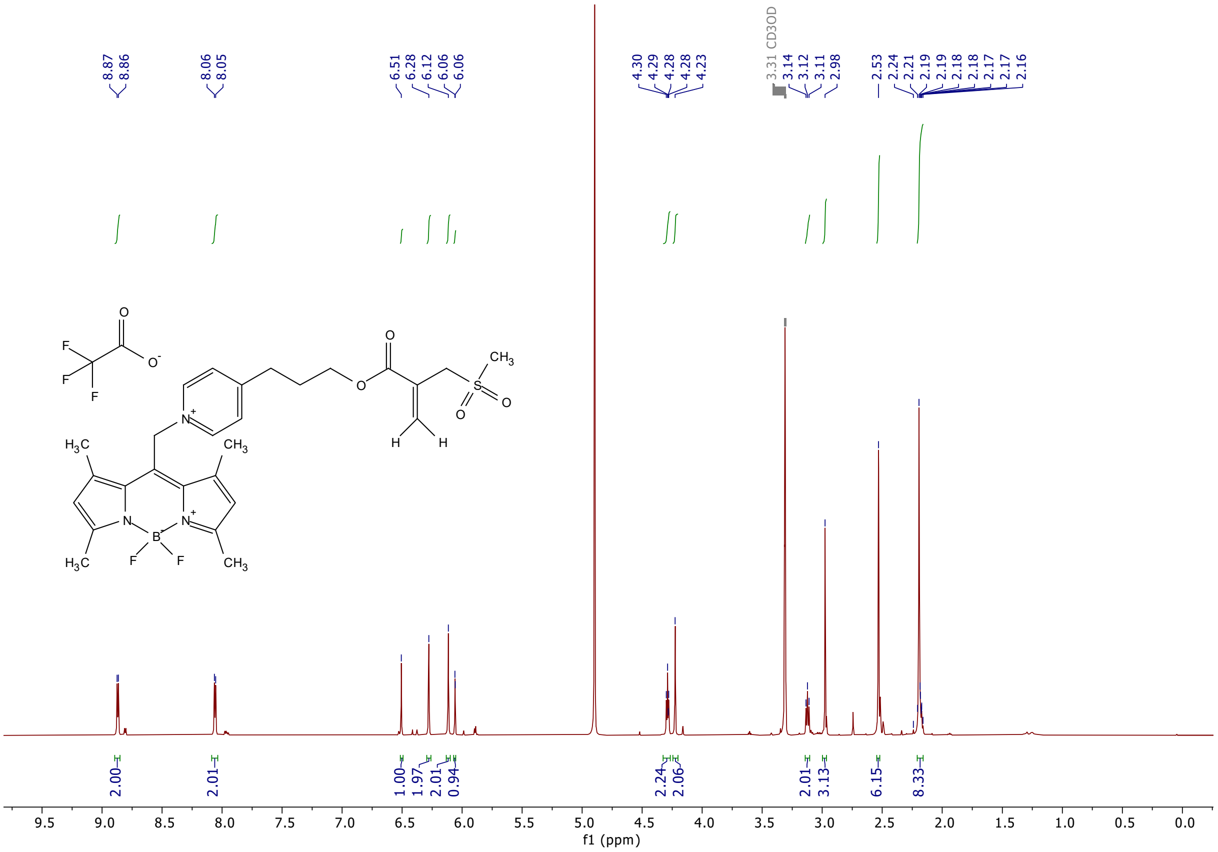

*Figure S47. ^{1}H NMR (600 MHz, CD_3OD) of **S7***

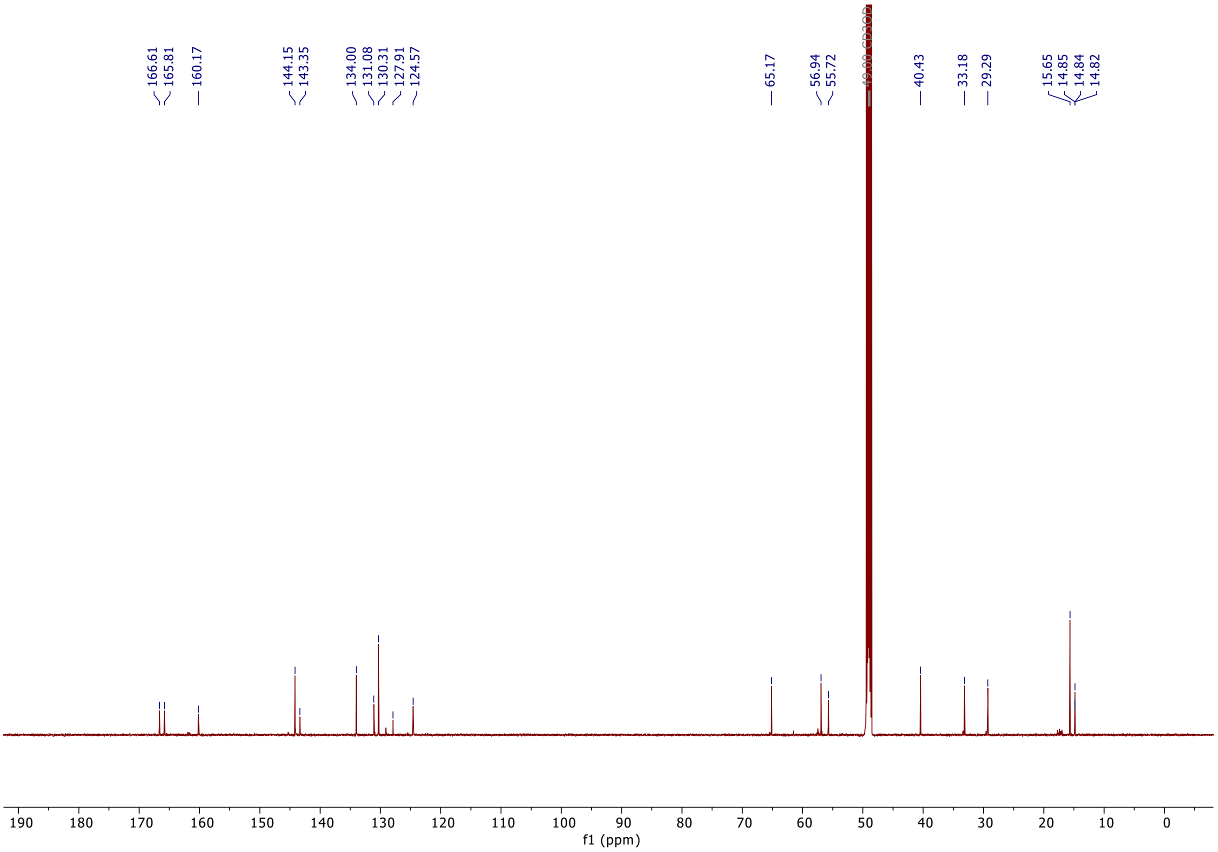

*Figure S48. ^{13}C NMR (151 MHz, CD_3OD) of **S7***

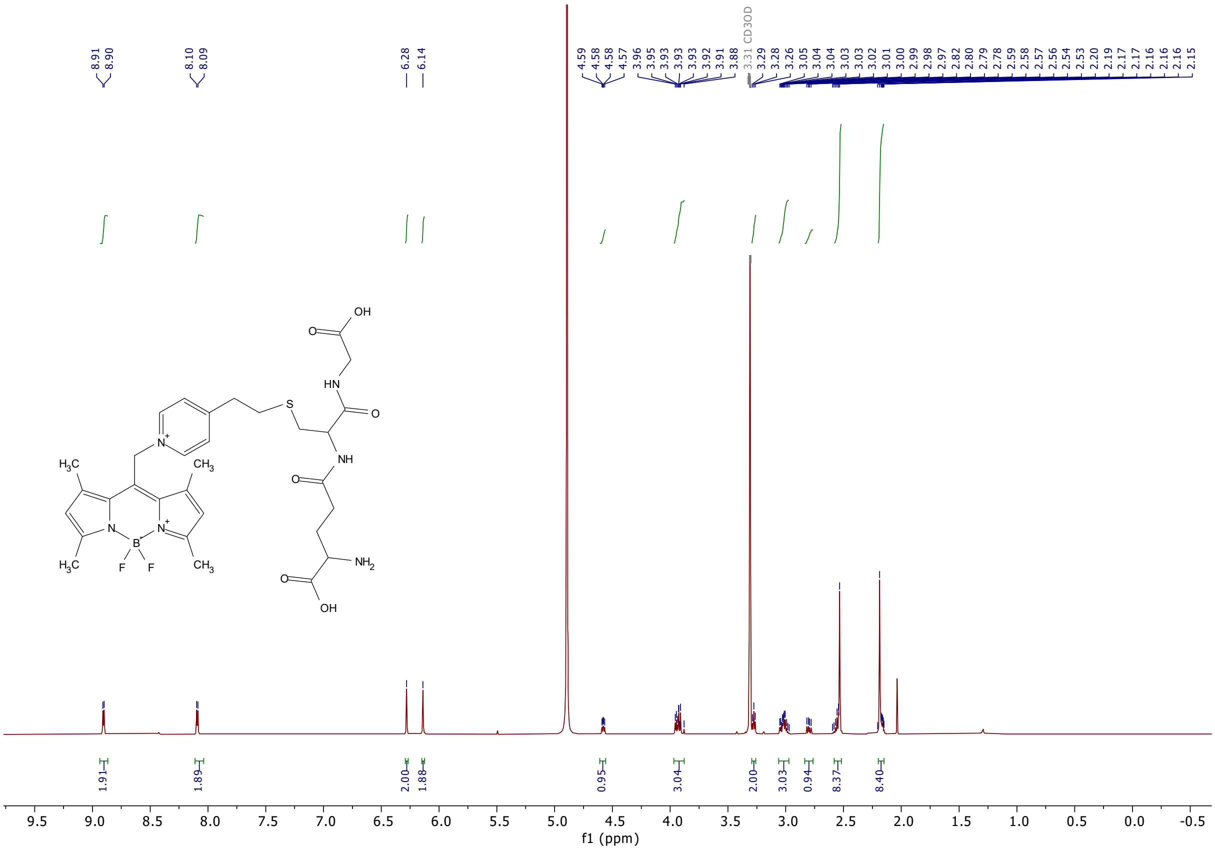

*Figure S49. ^{1}H NMR (600 MHz, CD_3OD) of **5**-GS*

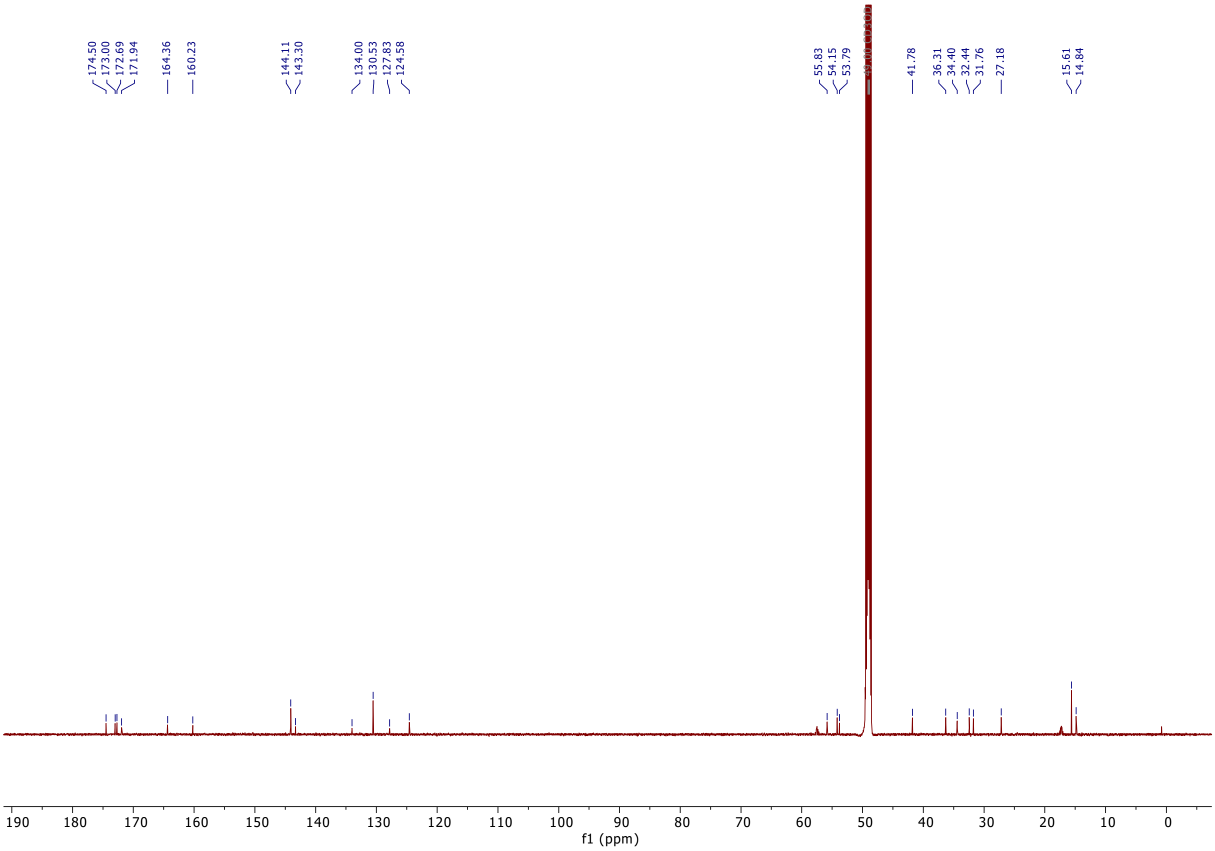

*Figure S50. ^{13}C NMR (151 MHz, CD_3OD) of **5**-GS*

11. References

1. Slanina T*, et al.* In Search of the Perfect Photocage: Structure-Reactivity Relationships in meso-Methyl BODIPY Photoremovable Protecting Groups. *J. Am. Chem. Soc.* **139**, 15168-15175 (2017).

2. Wang W, Zhou Y, Li Y. WO2022258005A1 (2022).

3. Sitkowska K, Feringa BL, Szymański W. Green-Light-Sensitive BODIPY Photoprotecting Groups for Amines. *J. Org. Chem.* **83**, 1819-1827 (2018).

4. Matos MJ*, et al.* Chemo- and Regioselective Lysine Modification on Native Proteins. *J. Am. Chem. Soc.* **140**, 4004-4017 (2018).

5. Matos MJ*, et al.* Quaternization of Vinyl/Alkynyl Pyridine Enables Ultrafast Cysteine-Selective Protein Modification and Charge Modulation. *Ang. Chem. Int. Ed.* **58**, 6640-6644 (2019).

6. Huang H-H*, et al.* Porous shape-persistent rylene imine cages with tunable optoelectronic properties and delayed fluorescence. *Chem. Sci.* **12**, 5275-5285 (2021).

7. Solomek T, Wirz J, Klan P. Searching for Improved Photoreleasing Abilities of Organic Molecules. *Acc. Chem. Res.* **48**, 3064-3072 (2015).

8. Harriman A, Mallon LJ, Ulrich G, Ziessel R. Rapid intersystem crossing in closely-spaced but orthogonal molecular dyads. *Chemphyschem* **8**, 1207-1214 (2007).

9. Frisch MJ*, et al.* Gaussian 16 Rev. C.01. Gaussian Inc. Wallingford, CT (2016).
